# The multiple roles of interferon regulatory factor family in health and disease

**DOI:** 10.1038/s41392-024-01980-4

**Published:** 2024-10-09

**Authors:** Lian Wang, Yanghui Zhu, Nan Zhang, Yali Xian, Yu Tang, Jing Ye, Fekrazad Reza, Gu He, Xiang Wen, Xian Jiang

**Affiliations:** 1 Department of Dermatology & Venerology, West China Hospital, Sichuan University, Chengdu, 610041 China; 2 Laboratory of Dermatology, Clinical Institute of Inflammation and Immunology, Frontiers Science Center for Disease-related Molecular Network, State Key Laboratory of Biotherapy, West China Hospital, Sichuan University, Chengdu, 610041 China; 3 State Key Laboratory of Southwestern Chinese Medicine Resources, School of Pharmacy, Chengdu University of Traditional Chinese Medicine, Chengdu, 611137 China; 4 Radiation Sciences Research Center, Laser Research Center in Medical Sciences, AJA University of Medical Sciences, Tehran, Iran; 5 International Network for Photo Medicine and Photo Dynamic Therapy (INPMPDT), Universal Scientific Education and Research Network (USERN), Tehran, Iran

**Keywords:** Target identification, Molecular medicine, Immunological disorders

## Abstract

Interferon Regulatory Factors (IRFs), a family of transcription factors, profoundly influence the immune system, impacting both physiological and pathological processes. This review explores the diverse functions of nine mammalian IRF members, each featuring conserved domains essential for interactions with other transcription factors and cofactors. These interactions allow IRFs to modulate a broad spectrum of physiological processes, encompassing host defense, immune response, and cell development. Conversely, their pivotal role in immune regulation implicates them in the pathophysiology of various diseases, such as infectious diseases, autoimmune disorders, metabolic diseases, and cancers. In this context, IRFs display a dichotomous nature, functioning as both tumor suppressors and promoters, contingent upon the specific disease milieu. Post-translational modifications of IRFs, including phosphorylation and ubiquitination, play a crucial role in modulating their function, stability, and activation. As prospective biomarkers and therapeutic targets, IRFs present promising opportunities for disease intervention. Further research is needed to elucidate the precise mechanisms governing IRF regulation, potentially pioneering innovative therapeutic strategies, particularly in cancer treatment, where the equilibrium of IRF activities is of paramount importance.

## Introduction

Interferon regulatory factors (IRFs) constitute a comprehensive category of transcription factors initially identified as regulators of type I interferon (IFN-I) and IFN-responsive genes, and their mechanisms have been extensively researched for over the past 30 years. To date, notable progresses have been made in elucidating the multifaceted and pivotal role of IRFs within the homeostatic defense mechanisms of the host and in orchestrating immune responses to both internal and external stimuli; notably, they are instrumental in enhancing antiviral responses, provoking pro-inflammatory reactions, and cell development and differentiation.^1–3^ Furthermore, IRF family members have been recognized to harbor dual roles in immunity, exhibiting both anticancer and cancer-promoting properties.^1^ IRF family members are implicated across a spectrum of human pathologies, encompassing infectious diseases, autoimmune and inflammatory disorders, metabolic dysfunctions, and oncogenesis.^4–9^

In mammals, nine IRF members have been reported,^10^ designated as IRF1 (also known as MAR), IRF2, IRF3, IRF4 (known alternatively as LSIRF/ICSAT/ PIP), IRF5, IRF6, IRF7, IRF8 (also termed as ICSBP), and IRF9 (also named as ISGF3γ or P48), each characterized by conserved multi-domain structures. Furthermore, an additional pair of IRF members, IRF10 and IRF11, have been discovered in avian species (IRF10) and teleost fish (IRF10 and IRF11), but these are notably absent in human and murine genomes.^11,12^ The inaugural member of the IRF family, IRF1, was unveiled by the Taniguchi laboratory in 1988, and it has been documented to facilitate virus-induced transcription by engaging with IFN-β enhancer elements.^13,14^ Later, Taniguchi’s group isolated the cDNA of IRF2 in 1989 by cross-hybridization with IRF1 cDNA.^15^ The IRF2 gene exhibits significant homology with the IRF1 gene within the 5’ portion of the protein-coding region.^15^ Additionally, IRF2 acts as an antagonist to IRF1, competing for the same promoter elements of IFN-I and IFN-II-inducible genes, thus potentially suppressing IRF1 function in specific contexts.^15^ In 1990, Driggers PH et al. characterized the IFN consensus sequence-binding protein (ICSBP), now identified as IRF8, initially cloned as a protein regulated by IFN-γ with a binding affinity for the IFN-inducible enhancer of major histocompatibility complex (MHC) class I genes.^16^ Moreover, Driggers PH and colleagues elucidated that ICSBP serves as a negative regulator, repressing the transcription of target genes activated by IFN or IRF1.^17^ Due to its derivation from a component of the IFNα-stimulated transcription factor 3 (ISGF3) complex, IRF9 was initially designated as ISGF3γ in 1990.^18^ ISGF3 constitutes a multiprotein complex comprised of four discrete polypeptides with molecular weights of 113, 91, 84, and 48 kDa, respectively.^19^ The 48 kD subunit is denoted as ISGF3γ, its expression level being upregulated by IFN-γ.^19,20^ ISGF3γ can associate with ISGF3α subunits, which are activated from a latent cytosolic form by IFN-I, to mediate antiviral activities. In 1995, Pitha group identified a novel IRF family member, IRF3, by mining homologs of IRF1 and IRF2 within an EST database, which associates with the IFN-stimulated response element (ISRE) to activate the expression of IFN-induced genes^21^ Furthermore, the Pitha group initially reported that IRF3 might potentiate transcription by forming complexes with other transcription factors, possibly members of the signal transducer and activator of transcription (STAT) family.^21^ In the same year, Matsuyama T. and colleagues identified a novel member of the IRF family, initially called the lymphoid-specific member of the IRF (LSIRF) and subsequently renamed IRF4.^22,23^ The cDNA for LSIRF was initially cloned from mouse spleen tissue, encoding a protein with 51 kDa molecular weight.^22^ IRF4 has been characterized using various terms in different contexts: as PU.1 interacting partner (PIP), it is a lymphoid-specific protein that binds to the enhancer elements of immunoglobulin light-chain genes, contingent on the presence of PU.1;^24^ as ICSAT, the human homolog of PIP and LSIRF, is isolated from adult T-cell leukemia cells or activated T cells.^25^ Distinctly, IRF4 differs from other IRF members as it is the sole IRF factor that is not regulated by IFNs.^22^ IRF4 induction can occur through a variety of antigen receptor-mediated stimuli, including plant lectins, CD3, and IgM cross-linking. Its function as a transcriptional activator or repressor is dictated by its interactions with an array of transcription factors or specific DNA-binding motifs^22,26^ IRF5 and IRF6, sharing structural homology, were first identified as members of the IRF family through the GeneBank database (accession numbers: human IRF5, U51127; human IRF6, AF027292).^27^ In 2001, Pitha group first demonstrated that IRF5 participated IFN-I gene induction.^28^ Prior research has established that IRF6 is predominantly engaged in developmental processes rather than in IFN gene expression, with its mutations being implicated in genetic disorders such as van der Woude syndrome, characterized by orofacial clefts and skin abnormalities.^29,30^ Likewise, IRF7 was initially identified as a novel constituent of the IRF family through the GeneBank database (accession numbers U73036, U73037) and information on IRF7 can be traced back to 1997^10,31^ The IRF7 cDNA was cloned by a yeast one-hybrid system, encoding proteins that interact with sequences in the Epstein-Barr virus (EBV) BamHI Q promoter.^31^ Exhibiting the greatest amino acid homology with IRF3, IRF7 possesses the ability to bind to the ISRE sequence, thereby inhibiting transcriptional activation mediated by both IFN and IRF1.^10,31^

In recent decades, substantial advancements have been made in elucidating the regulatory mechanisms of IRF family members and their diverse roles in various diseases. A majority of these members are crucial for cellular development, immunity, inflammation, and oncogenesis, serving as in human diseases.^32–34^ However, IRFs may exhibit distinct regulatory effects depending on cell type and environmental context, rendering their roles complex and at times paradoxical and double-edged swords in human health. This review primarily synthesizes the structural characteristics, post-translational modification sites, biological roles, and associated signaling pathways of the IRF family, alongside an exploration of diseases linked to these genes and proteins, with a focus on infections, inflammatory conditions, and a spectrum of cancers, encompassing but not limited to cardiovascular, pulmonary, urinary, reproductive, and skin systems. This review provides a comprehensive insight into the IRF family, underscoring the significance of IRFs as emerging biomarkers and potential therapeutic targets in the realm of human diseases.

## IRF protein family: genes, proteins and structures

**Fig. 1:**
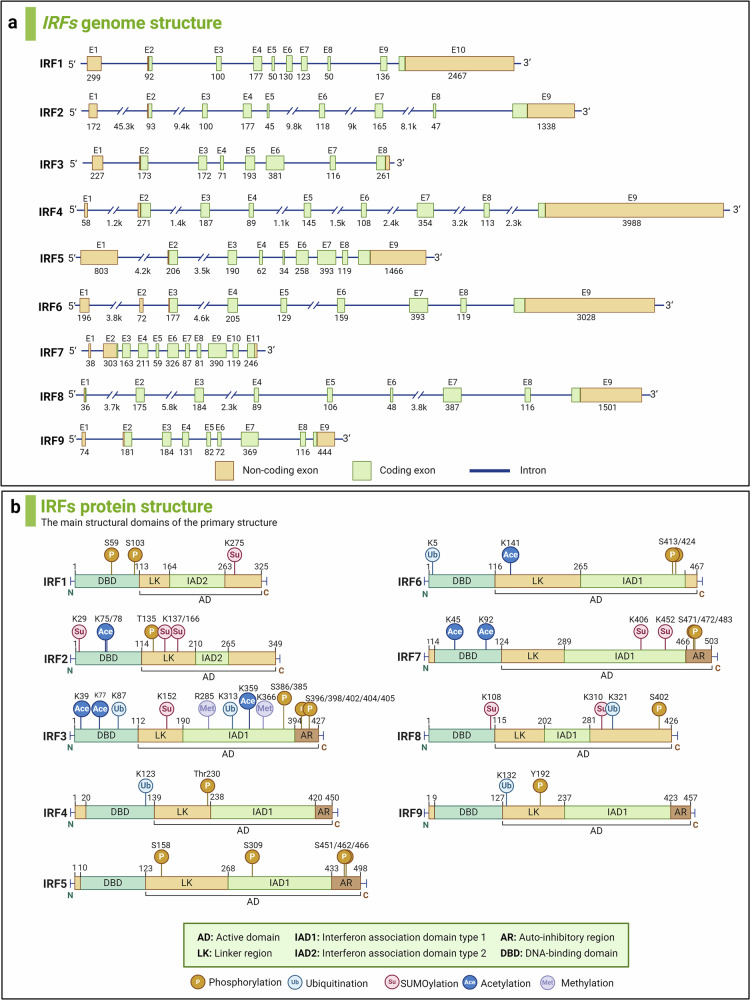
The genes and proteins structures of IRF family members. **a** The genomic structures. The boxes mark the exons, including non-coding exons (orange) and coding exons (green). blue lines mark the introns. Start and stop codons are indicated. The numbers below the genes indicate the sizes of the exons and introns. **b** The structures of IRF family proteins. DBD represents the DNA-binding domain, LK represents the linker region, IAD represents interferon association domain, and AR represents auto-inhibitory region. Active domains (AD) are also marked on the figure. The upper numbers refer to the starting amino acid sites of diferent domains, and the different post-translational modifications of the IRFs are presented, including Phosphorylation, Ubiquitylation, SUMOylation, Methylation, and Acetylation

The detailed genes and proteins of IRF family members are presented in Fig. 1. The IRF family encompasses a cohort of transcription factors integral to the regulation of IFNs and the orchestration of immune response. IRF proteins regulate the expression of target genes through their interaction with ISREs or IFN regulatory elements within the DNA. All IRFs feature a highly conserved N-terminal DNA-binding domain (DBD), comprising around 120 amino acids that constitute a helix-turn-helix motif. This motif is essential for the identification of specific DNA sequence elements (A/GNGAAANNGAAACT), known as ISRE, present in the promoters of genes for IFN-I, IFN-III, and IFN-stimulated genes (ISGs).^27^ The C-terminal regions of IRFs contain a conserved domain known as IFN association domain1 (IAD1)(IRF3, IRF4, IRF5, IRF6, IRF7, IRF8, and IRF9) or IAD2(IRF1 and IRF2) that have low sequence homology and serve as association domains by which IRFs interact with other IRF members or transcription factors and/or cofactors.^1^ Many IRF family proteins also contain a regulatory region between the DBD and IAD, which includes multiple phosphorylation sites.

### IRF1 and IRF2

The human IRF1 gene maps to chromosome 5q31.1, spanning 9,165 base pairs (bp) and comprising 10 exons and 9 introns. Human IRF1 protein has an approximate molecular weight of 45 kDa and is composed of 325 amino acids. The IRF1 gene is constitutively expressed in human cells at low basal levels, but can be triggered by diverse stimuli, such as IFNs, tumor necrosis factor (TNF), and interleukin-1 (IL-1).^35^ The human IRF2 gene is located on chromosome 4q35.1 with 86,822 bp, and contains 9 exons and 8 introns. Through alternative splicing, IRF2 produces two splice variants. Currently, the primary focus of research has been on isoform 1 of IRF2, the full-length variant, which encodes 349 amino acids. IRF2 is constitutively expressed in multiple cell types and its expression can be further induced by viruses and IFN. Despite sharing a similar DBD with IRF1, IRF2 frequently serves an antagonistic role in immune regulation by inhibiting the activation of genes such as IFN-β. This inhibition is achieved through competitive binding to shared sites, thereby functioning predominantly as a suppressor of IRF1-mediated gene activation.

### IRF3 and IRF7

The human IRF3 gene is situated on chromosome 19q13.33, spans 6286 bp, and contains 8 exons and 7 introns. A variety of splicing variants of IRF3 exist, with a minimum of five distinct variants identified thus far. The most extensively investigated variant of IRF3 is isoform 1, which is approximately 50 kDa and encodes 427 amino acids. IRF3 is constitutively expressed in many tissues, and the relative steady-state levels of IRF3 mRNA do not increase by virus infection or IFN treatment in cells.^21^ Situated on chromosome 11p15.5, the human IRF7 gene spans 1923 bp and contains 11 exons and 10 introns. The human IRF7 gene encodes four alternative splicing variants, named IRF7A, IRF7B, IRF7C, and IRF7H. ^31,36^ Among these, the variant that has been most thoroughly investigated is IRF7A, which is composed of 503 amino acids and has a molecular weight of 55kD. The C-terminal region of IRF7 contains multiple regulatory domains for its activation.^37^ IRF7, sharing a close relationship with IRF3, operates as a transcription factor that requires activation. The virus-activated domain in human IRF7A is vital for the activation of IRF7.^38^ IRF7 is inherently present in the cytoplasm, with its expression predominantly observed in B cells, plasmacytoid dendritic cells (pDCs), and monocytes in spleen, thymus, and peripheral blood leukocytes.^31,36^ Various stimuli, including Lipopolysaccharide (LPS), IFN-α, EBV-latent membrane protein 1 (EBV-LMP1), viral infections, and certain chemical agents like phorbol myristate acetate (PMA) and sodium butyrate, can significantly induce the expression of IRF7 in specific cell lines.^36,39,40^ Additionally, DNA damage agents, virus infection, and EBV-LMP1 can activate IRF7 phosphorylation and nuclear translocation.^38,41,42^

### IRF4 and IRF8

The human IRF4 gene maps to chromosome 6p25.3 with 19,692 bp, contains 9 exons and 8 introns, and gives rise to two splicing variants. The predominant isoform of IRF4, the full-length variant, encodes a protein of approximately 51 kDa, containing 451 amino acids. IRF4 is uniquely expressed within immune system cells and is responsive to various mitogenic stimuli, including PMA/Ionomycin and T-cell receptor (TCR) cross-linking.^22,24,25,43^ IRF4’s role as a transcriptional activator or suppressor is determined by its interactions with various transcription factors or the DBD on distinct promoters.^44,45^ The human IRF8 gene is situated on a 23 kb segment of chromosome 16q24.1, and contains 9 exons and 8 introns. IRF8 protein encodes 426 amino acids. IRF8 is predominantly constitutively expressed in lymphoid and myeloid cell lines and induced by IFN-γ, but also in epithelial cells of the intestine, skin, lung, liver, ocular lens, cornea, and heart.^46–48^ IRF8 is highly expressed in both progenitor and mature cells within the B cell, conventional DC1 (cDC1), and pDC lineages, playing a crucial role in their development and functionality.

### IRF5 and IRF6

Situated on chromosome 7q32.1, the human IRF5 gene spans 13,007 bp, and comprises 9 exons and 8 introns. Numerous splice variants of IRF5 have been identified, with at least six distinct variants documented thus far. The size of the encoded IRF5 protein varies depending on the splice variant. One of the most extensively studied isoforms of IRF5 is the V1, which encodes a protein approximately 57 kDa and consists of about 498 amino acids. Initially discovered as a key regulator of IFN-I gene expression.^28^ IRF5 is primarily found in B cells, monocytes, macrophages, and pDCs. During viral-triggered activation of IFN-I genes, there are multiple spliced isoforms of IRF5, each characterized by different patterns of expression specific to cell types, cellular localization, differential regulatory mechanisms, and divergent functional roles.^49^ The human IRF6 gene maps to chromosome 1q32.2 with 20,526 bp and contains 9 exons and 8 introns. Likewise, IRF6 can produce two primary splice variants (variant 1 and variant 2) via alternative splicing. The existence of these splice variants enables IRF6 to perform distinct roles across diverse cell types and under various physiological conditions. The encoded human IRF6 protein is typically around 56 kDa and consists of approximately 467 amino acids. IRF6 exhibits prominent cytoplasmic expression in various cell types and is crucial for embryonic development, particularly in the epidermal and oral mucosa formation.

### IRF9

The human IRF9 gene is situated on chromosome 14q12 with 5301 bp, and contains 9 exons and 8 introns. Human IRF9 protein encodes 393 amino acids. IRF9 is ubiquitously expressed in various cell types and has been identified as playing an essential role in the antiviral defense mechanism mediated by IFN-α/β and IFN-γ.^50^

## Post-translational modifications (PTMs) of the IRFs

PTMs are covalent alterations that modify the properties of a protein through adding a modifying chemical group or peptide moieties to one or several of its amino acid residues.^51^ A singular protein may undergo modifications by multiple PTM types or be recurrently modified by the same PTM at distinct residues. Key PTMs influencing IRFs encompass phosphorylation, ubiquitination, SUMOylation, methylation, and acetylation, each playing a pivotal role in dictating the functional dynamics, proteostasis, and conformational integrity of these transcription factors (Fig. 1b).

### Phosphorylation

IRFs undergo various PTMs, among which phosphorylation stands out as the most critical. Glycogen synthase kinase 3β mediates dual phosphorylation of IRF1 at Thr181 and Ser185, which is required for the regulation of IRF1 turnover by K48-linked polyubiquitination proteasomal degradation.^52^ Moreover, IκB kinaseε (IKKε) mediates the phosphorylation of the C-terminal region of IRF1, significantly diminishing the stability of IRF1, accelerating it, and inhibiting the transcriptional activity of IRF1.^53^ In addition, IRF1 undergoes phosphorylated at Tyr109 within the DBD.^54,55^

Phosphorylation is a crucial PTM of IRF3, as it induces cytoplasm-to-nucleus translocation of phosphorylated IRF3, and stimulates DNA binding and transcriptional activity.^56^ The two kinases, IKKε and TANK -binding kinase 1 (TBK1) are the major phosphorylation kinases of IRF3.^57–59^ IRF3 features two significant phosphorylation sites: site 1 includes Ser385 and Ser386, whereas site 2 includes Ser396, Ser398, Ser402, Thr404, and Ser405 within the C-terminal region.^56,60,61^ Moreover, c-Jun N-terminal kinase (JNK) can stimulate the phosphorylation of IRF3 at Ser173in the N-terminal.^62^ In addition, IFN-I-induced long noncoding RNA-ISIR binds IRF3 at DBD promoting its phosphorylation, dimerization, and nuclear translocation upon infection, consequently facilitating IRF3 activation.^63^ Recently, Wang et al. found that serine/threonine-protein kinase 38-like phosphorylates IRF3 at Ser303, which prevents IRF3 degradation mediated by the proteasome in the rest state.^64^ However, mammalian sterile 20-like kinase 1-mediated IRF3 phosphorylation at Thr75 and Thr253 severely disrupted the ability of activated IRF3 to form homodimerization that impairs its transcriptional responses.^65^

The phosphorylation sites of IRF4 include Tyr121 and Tyr124. LMP1 promotes IRF4 tyrosine phosphorylation and significantly stimulates its transcriptional activity.^66^ Moreover, Rho-associated coiled-coil-containing protein kinase 2 can phosphorylate IRF4 to regulate the production of IL-17 and IL-21.^67^ In microglia, IL-1 receptor associated kinase 4 (IRAK4) phosphorylates both IRF4 and IRF5 by forming a Myddosome with myeloid differentiation primary-response protein 88 (MyD88)/IRF5/IRF4.^68^

The phosphorylation sits of IRF5 include Ser158, Ser309, Ser451, Ser466, and Ser462.^69,70^ In myeloid cells, phosphorylation at Ser462, facilitated by IKKβ, activates IRF5, which triggers IRF5 dimerization of and subsequent nucleus translocation.^71^ However, IKKα-induced phosphorylation of IRF5 has an inhibitory effect on the transcriptional activation of IFN-I and the promoters of inflammatory cytokines.^72^

The phosphorylation of IRF6 at Ser413 and Ser424 primes IRF6 for activation.^73^ In addition, receptor interacting protein kinase 4 (RIPK4) directly regulates the trans-activator activity and nuclear translocation of IRF6 via phosphorylating its C-terminal domain at Ser413 and Ser424.^74^

The kinases implicated in the phosphorylation of IRF7 include IKKε, IKKα, IRAK1, and TBK1.^57,75–77^ In response to viral infection, IRF7 is phosphorylated at Ser477 and Ser479 and activated by TBK1 and IKKε.^38^ Additionally, phosphorylation at Ser471, Ser472, Ser483, Ser484, and Ser487 also contributes to the activation of IRF7. However, the exogenous expression of protein phosphatase 1(PP1) subunits, heat shock protein 70, vaccinia virus E3L protein, and the open reading frame 45 of Kaposi’s sarcoma-associated herpesvirus inhibit IKKε-stimulated IRF7 phosphorylation and significantly reduce IRF7 transcriptional activity.^78–81^

### Ubiquitylation

Ubiquitination is a universal reversible PTM that can either activate or deactivate protein, with IRFs being tightly regulated by ubiquitination in various respects.^82^ Ubiquitination can either positively or negatively influence the stability, activation, and transcriptional activity of IRFs. The fundamental element of ubiquitination is ubiquitin, which is covalently attached to one or more lysine residues in cellular involving three classes of enzymes.^83^ Ubiquitin itself contains seven lysine residues and one N-terminal methionine residue. Each of these can be further conjugated with another ubiquitin to form ubiquitin chains of different linkages.

IRF1 is characterized by a brief, undergoing rapid degradation through the ubiquitin-proteasome pathway.^84^ K63-linked ubiquitination of IRF1 contributes to its activation, while K48-linked ubiquitination contributes to its degradation.^85^ Notably, HIV-1 viruses have developed strategies to exploit this ubiquitin-proteasome pathway to inactivate IRF1 function and evade the host immune defense mediated by IRF1.^86^ IRF1 K63-linked ubiquitination is mediated by TNFR-associated factor 6 (TRAF6) and cellular inhibitor of apoptosis 2 (clAP2).^87^ In response to IL-1 stimulation, clAP2 mediates the K63-linked polyubiquitination of newly synthesized IRF1, leading to its activation.^88^

MGF360-14L, a viral non-structural protein, facilitates the degradation of IRF3 via tripartite motif-containing protein (TRIM) 21-mediated K63-linked ubiquitination of IRF3.^89^ Cellular E3 ligases c-Cbl, RTA-associated ubiquitin ligase (RAUL), RBCC protein interacting with PKC1 (RBCK1), and Midline-1 (MID1) target IRF3 for K48-linked polyubiquitination, leading to its proteasome-dependent degradation. Viral infection leads to the induction of RBCK1, which subsequently catalyzes the ubiquitination and degradation of IRF3.^90^ During viral infection, MID1 inhibits IFN-I production by interacting with IRF3 and negatively regulating IRF3 protein levels.^91^ MID1 induces the ubiquitination of IRF3 at Lys313 playing a role in the cellular antiviral response, which is governed by a negative feedback mechanism. Jumonji domain-containing protein 6 promotes activated IRF3 K48 ubiquitination and degradation by ring finger protein 5.^92^ Sentrin/SUMO-specific protease 2 catalyzes K48-linked ubiquitination of IRF3 at Lys87 and competitively promotes IRF3 deSUMOylation at Lys70.^93^

IRF3 ubiquitination not only leads to its degradation but also contributes to its activation. Specifically, the activation of IRF3 in the RLR-induced IRF-3-mediated pathway of apoptosis (RIPA) requires linear polyubiquitination of two specific lysine residues on IRF3 by the linear polyubiquitinating enzyme complex.^94^ However, this pathway can be inhibited by Otulin, a deubiquitinase, which removes linear polyubiquitin chains, resulting in IRF3 deubiquitinating.^95^

Moreover, E3 ligase ring finger protein 2 (RNF2) promotes the ubiquitination and degradation of IRF4 in colon cancer.^96^ MiR-155-5p contributes to the development of childhood acute lymphoblastic leukemia (ALL) by the Casitas B-lineage lymphoma (CBL)-mediated degradation of IRF4 via ubiquitination.^97^ In addition, CBLs exhibit elevated expression levels in germinal center light zone B cells, where they promote the ubiquitination and degradation of IRF4.^98^ However, the ubiquitin specific peptidase 4 (USP4) interacts with and deubiquitinate IRF4 to stabilize IRF4 protein, thereby promoting IRF4 function to facilitate IL-4 expression in Th2.^99^

K63-linked ubiquitination of IRF5 contributes to its activation and increases its nuclear translocation.^100^ TARF6 and Pellino-1 have been identified as the ubiquitin E3 ligases for IRF5, which target and promote K63-linked ubiquitination of IRF5. In human and mouse M1 macrophages, the interaction between Pellino-1 and IRF5 in the cytoplasm activates IRF5 and increases its nuclear translocation via K63-linked ubiquitination.^101,102^

The RTA immediate-early nuclear transcription factor, which is encoded by Kaposi’s sarcoma-associated herpesvirus, facilitates the ubiquitination and degradation of the IRF7 protein in a proteasome-dependent.^103^ Additionally, RAUL regulates IFN-I via targeting both IRF7 and IRF3 for K48-linked polyubiquitination and proteolysis.^104^ LMP1-induced antiapoptotic factor A20 possesses both deubiquitinase and ubiquitin E3 ligase activities, and negatively regulates LMP1-stimulated IRF7 K63-liked ubiquitination and activity during EBV latency.^105^ N-Myc and STATs interactor is a Sendai virus-inducible protein, which promotes the IRF7 K48-linked ubiquitination and proteasome-dependent degradation.^106^ Similarly, TRIM35 also promotes the K48-linked ubiquitination of IRF7 and induces its degradation via a proteasome-dependent pathway. The interaction of Fas-associated death domain (FADD) with TRIM21 can enhance TRIM21’s ubiquitin ligase activity, both of them can directly ubiquitinate IRF7, affect its phosphorylation status, and interfere with TRAF6 ubiquitin ligase activity.^107^

Additionally, IRF7 can be activated by the EBV-LMP1 via receptor interacting protein (RIP)-dependent K63-linked ubiquitination.^108^ Further studies found that both TRAF6 and its E3 ligase activity are required for LMP1-stimulated IRF7 ubiquitination. IRF7 is ubiquitinated by TRAF6 at multiple sites, but the K63-linked ubiquitination sites are independent of its C-terminal functional phosphorylation sites.^109^ Nevertheless, TAR RNA binding protein 2, an inhibitor of IRF7, inhibits the K63-linked ubiquitination and phosphorylation of IRF7.^110^

CBL also mediates IRF8 ubiquitination, leading to the degradation of IRF8.^111^ Ro52 (also called TRIM21) can interact with and ubiquitinate IRF8 in a non-degradation pathway. This interaction in turn enhances IL-12p40 expression in an IRF8-dependent manner.^112^ Similarly, USP4 also stabilizes IRF8 protein levels in regulatory T cells (Tregs) by interacting with IRF8 via a K48-linked deubiquitinase.^113^ Likewise, IRF9 can be ubiquitinated and degraded by herpes simplex virus (HSV) type 2 ICP22 protein, which functions as a novel E3 ubiquitin protein ligase.^114^

### SUMOylation

SUMOylation, is a critical PTM that is catalyzed by a limited set of modifying enzymes yet dynamically regulates a vast array of target proteins. Small ubiquitin-like modifiers (SUMOs) are members of the ubiquitin-like family of proteins that predominantly target nuclear proteins.^115^ Five SUMO family members have been identified in mammals.^116^ SUMOylation is pivotal in the regulation of nuclear processes, including transcription, nuclear body formation, nucleocytoplasmic transport, RNA processing, cell cycle progression, DNA repair, chromosomal functions, and signal transduction.^117,118^ Similar to ubiquitylation, SUMOylation is reversible, as SUMO proteases can remove SUMOs from their substrates.^118,119^ IRFs are also regulated by SUMOylation

Lys275 is identified as the primary SUMOylation site of IRF1. The protein inhibitor of activated STAT3 serves as both a SUMO-1 ligase and an inhibitor of IRF1’s transcriptional functions.^120^ Additionally, the SUMO-conjugating enzyme Ubc9 suppresses IRF1’s transcriptional activation by inducing IRF1 SUMOylation.^121^ Moreover, some studies have demonstrated that SUMOylated IRF1 may act as an oncogenic protein in tumor cells.^122^ SUMOylated IRF1 is elevated in tumors, which inactivates its tumor suppressor function by repression of its transcriptional activity that facilitates resistance to the immune response.^123^ Furthermore, treatment with alpha-lipoic acid induced IRF1 SUMOylation by increased SUMO-1 in an IL-1β-stimulated chondrocyte model.^124^ The level of SUMOylated IRF1 was significantly elevated in the myocardial infarction (MI) group treated with 5-azacytidine.^125^

IRF2 SUMOylation sites include Lys137, Lys293, and Lys166. SUMOylation of IRF2, catalyzed by SUMO-E3 ligase PIASy, represses its transcriptional activity in an histone deacetylase (HDAC)-dependent manner.^126^ SUMOylation of IRF2 has minimal effects on its nuclear localization and DNA-binding activity. However, it enhances the inhibition of IRF1’s transcriptional activity while reducing the capacity to activate ISRE and H4 promoters.

During vesicular stomatitis virus infection, both IRF3 and IRF7 undergo phosphorylation as well as modification by SUMO1, SUMO2, and SUMO3.^127^ The SUMOylation of IRF3 at Lys152 and IRF7 at Lys 406 is independent of their phosphorylation, and vice versa. However, some studies have found that SUMOylation of IRF3 leads to a reduction in IRF3 phosphorylation and IFN synthesis.^128^ SUMOylation of IRF3 and IRF7 negatively regulates IFN transcription.^127^ The Ebola Zaire virus VP35 protein inhibited IFN transcription in DCs by increasing PIAS1-mediated SUMOylation of IRF7.^129^ The SUMO conjugation sites of IRF7 include Lys406 and Lys452.^127,130^ The EBV-LMP1 can limit the capacity of IRF7 to initiate innate immune responses via promoting IRF7 SUMOylation at Lys452.^130^ The TRIM28 is a specific SUMO E3 ligase and a negative regulator of IRF7.^131^

In addition, Ubc9-mediated IRF4 SUMOylation enhanced its nuclear localization and stability.^132^ Similarly, IRF5 and IRF8 can undergo SUMOylated.^133^ However, the SUMOylation of IRF8, catalyzed by SUMO3 at the Lys310, can be reversed by SUMO-specific protease (SENP) 1 and SENP3.^134,135^

### Methylation and acetylation

Protein methylation refers to the transfer of methyl to a protein residue. Acetylation modification is a reversible and evolutionarily conserved PTM. Some IRFs (IRF1, IRF2, IRF3, IRF7, and IRF9) also undergo methylation or acetylation.

In U937 cells treated with PMA, both IRF1 and IRF2 undergo acetylation facilitated by p300 and p300/ CREB-binding protein (CBP)-associated factor (PCAF).^136^ The p300-mediated IRF1 acetylation sites include the N-terminal Lys39 and Lys78.^137^ In addition, IRF1 can be specifically acetylated by KAT8 at Lys78.^138^ The acetylation sites of IRF2 include N-terminal DBD Lys75 and Lys78. Furthermore, some studies have shown that the acetylation of IRF2 is dependent on cell growth.^139,140^ The monomethylation of IRF3 at Lys366 by nuclear receptor-binding SET domain 3 (NSD3), enhances the transcriptional activity of IRF3 in antiviral innate immunity.^141^ This is because NSD3-mediated IRF3 methylation obstructs IRF3 dephosphorylation by disrupting PP1’s association with IRF3, thereby maintaining IRF3 phosphorylation.^142^ Furthermore, LPS induces the arginine methylation of IRF3 at Arg285, leading to its dimerization and promoting its translocation from the cytoplasm to the nucleus.^143^

KAT8 mediates IRF3 acetylation at Lys359 through its MYST domain, which leads to the inhibition of IRF3 recruitment to IFN-I gene promoters and a decrease in its transcriptional activity.^144^ In vivo, IRF7 undergoes acetylation at Lys92 located in the DBD by the histone acetyltransferases PCAF and GCN5, resulting in impaired DNA binding capability.^145^ A subsequent study has shown that sirtuin1 (SIRT1) -mediated DBD deacetylation is a pivotal mechanism in the activation of IRF3 and IRF7.^146^ Upon viral stimulation, viral interferon regulatory factor 3 engaged liquid-liquid phase separation with ISRE DNA and compartmentalized IRF7 in the nucleus, thus stimulating the expression of IFN-I. In addition, IRF5 and IRF9 also undergo lysine acetylation.^147,148^

## Biological functions of IRFs

### Regulation of immune cell development

**Fig. 2:**
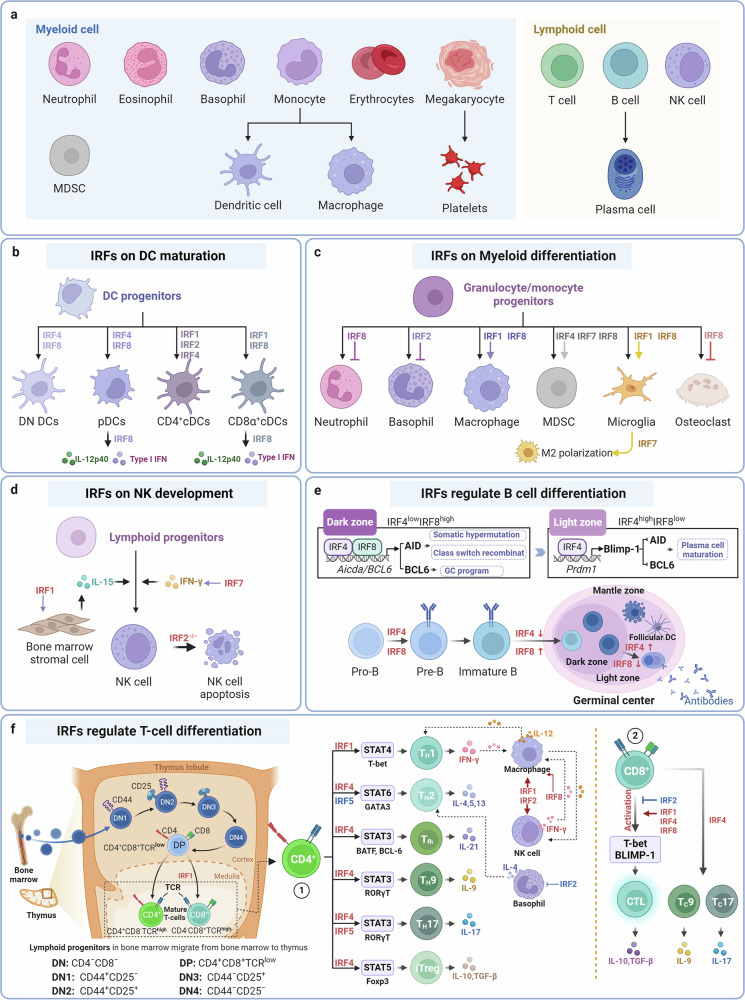
The regulatory effects and molecular mechanisms of IRFs on immune cell development. **a** The subtypes of myeloid cells and lymphoid cells. **b** The induction effect of IRFs on DC maturation and cytokine production. **c** The role of IRFs in myeloid differentiation and MDSC aggregation. **d** Regulation of IRFs in Natural Killer Cells development. **e** Multistep regulation of B cells and plasma cells by IRFs. **f** IRFs regulate T cell development and differentiation

Diverse studies have shown that the IRF family can regulate immune cell differentiation. Herein, IRF family members with their functions and molecular mechanisms in immune cells are discussed in detail. The subtypes of myeloid cells and lymphoid cells are pesented in Fig. 2a.

#### The induction effect of IRFs on DC maturation and cytokines production

DCs constitute a specialized subset of hematopoietic cells endowed with the capacity to secrete IFN-I and a plethora of other cytokines. As the quintessential antigen-presenting cells (APCs), DCs are instrumental in initiating both innate and adaptive immune responses.^149^ Characterized by their high expression of MHC molecules, DCs, upon detecting invading pathogens via pattern recognition receptors (PRRs), secrete various cytokines and present antigen-MHC complexes to T cells, thereby eliciting helper T cell (Th) responses or inducing immunological tolerance.^150^

DCs constitute a heterogeneous population, comprising subpopulations with distinct functionalities, which can be categorized into conventional DCs (cDCs) and pDCs based on unique surface markers. In mice, spleen DCs are further subdivided into at least four subgroups: CD4^+^ DCs, CD8α^+^ DCs, CD4^-^CD8α^-^ (double negative, DN) DCs, and pDCs.^150^ In humans, cDCs are bifurcated into two subgroups: CD141^+^cDC1 and CD1c^+^DC2 cells, with their murine counterparts being CD11b^-^CD103^+^ (CD8α^+^) cDC1 and CD11b^+^cDC2. These cell populations, characterized by different gene expression profiles, perform distinct functions. pDCs are prolific producers of IFN-I, whereas cDCs generate both pro-inflammatory and anti-inflammatory cytokines, such as IL-10 and IL-12. cDC1 cells are pivotal for eliciting CD8^+^T cell responses under viral or tumoral challenges, while cDC2 cells exhibit a robust capacity to initiate CD4^+^T cell responses.^151^

The IRF family proteins governs the differentiation and functional activity of DCs (Fig. 2b). IRF4 and IRF8 are selectively instrumental in the development of specific DC subgroups.^152,153^ These two IRFs share overlapping roles in driving the general process of DC development, yet they also possess distinct activities that stimulate the expression of subgroup-specific genes, resulting in the emergence of functionally diverse DC subpopulations. During DC development, DN DCs appear to represent the prototypical DC subset, differentiating into various DC subtypes and functions under the differential regulation of IRFs. IRF4 is crucial for producing CD4^+^DCs, while CD8α^+^ DCs require IRF8. Both of the two IRFs are instrumental in the development of CD4-CD8α- DCs and pDCs.^154^ These cells express both IRF4 and IRF8, with CD8α^+^ DCs expressing high levels of IRF8 and CD4^+^DCs predominantly expressing IRF4.^154^ In *Irf8*^-/-^ mice, there is a notable absence of CD8α^+^ DCs and pDCs, whereas in *Irf4*^-/-^ mice, the numbers of CD4^+^ DCs are significantly diminished.^152,155,156^ Mice deficient in both IRF8 and IRF4 retain only a minor population of CD4^-^CD8α^-^ DCs, completely lacking other DC subtypes in the spleen.^154^

Without IRF8, committed cDC1 cells acquire transcriptional, functional, and chromatin accessibility characteristics reminiscent of cDC2 cells. While IRF8 is not essential for the survival of committed cDC1 cells, it is critical for preventing their transdifferentiation into cDC2-like cells.^157^ Furthermore, IRF1 and IRF2 also modulate the development of DC subgroups.^158,159^ In *Irf*^*1*-/-^ mice, pDCs predominate, while the cDC population, particularly the CD8α^+^ subset, is selectively diminished.^158^ The capacity of IRF1-deficient spleen DCs to produce pro-inflammatory cytokines such as IL-12 is significantly compromised. In *Irf2*^-/-^ mice, spleen CD4^+^CD11b^+^DCs exhibit pronounced selective deficits.^159^

IRF proteins also regulate the induction of IFN-I in DCs.^160^ Among them, IRF3 and IRF7 are widely recognized as crucial transcription factors for IFN induction.^32,161^ IRF7, activated under toll like receptor (TLR) signaling, promotes IFN induction in pDCs, cDCs, and non-DC cells.^160,162–164^ IRF3 primarily facilitates IFN induction in fibroblasts and is not necessary for IFN induction in DCs.^151^ IRF3 is the primary early regulatory factor that induces IFN-I during intracellular viral infection.^165^ While IRF5 is not required for IFN induction, it can enhance the production of non-IFN pro-inflammatory cytokines through distinct mechanisms.^160,163,166^ However, surprising findings from in vivo studies suggest the existence of an alternative IFN induction pathway that operates independently of IRF3, IRF5, and IRF7. It was expected that IRF3-5-7 triple knockout mice would exhibit impaired IFN-I production, yet IFN-I activity was still detected in their serum.^167^ The most likely candidate responsible for this induction appears to be IRF1. IRF1 is expressed widely and can enhance early IFN production by modulating the phosphorylation and localization of IRF3.^168^ It may also compensate for the role of IRF7 as a positive regulator of IFN, establishing a positive feedback mechanism to sustain IFN production.^32^ The generation of IFN-I in DCs is heavily reliant on IRF8, particularly during the feedback phase of IFN gene induction. Exogenous expression of IRF8 can rescue the development of pDCs and CD8α^+^ DCs in vitro, triggering IFN-I and IL-12p40 production, whereas IRF4 does not exert the same effect.^169^ Conversely, the introduction of IRF4 can restore the expression of selectively expressed genes in CD4^+^DCs. The activation of IFN-I in pDCs is mediated by a MyD88-dependent signaling pathway, reliant on TLR3 and retinoic acid inducible gene I (RIG-I)-like receptors(RLRs).^160^ The cytokines produced by activated DCs, including IFN, subsequently promote DC maturation and alter DC phenotypes and functions, with IRF family proteins potentially involved in regulating these processes.

Beyond IFN-I, IRF family proteins are also pivotal in the induction of other pro-inflammatory cytokines. IL-12p70, produced by APCs and B cells, is a heterodimeric pro-inflammatory cytokine secreted into the extracellular milieu.^170^ IL-12 can induce IFN-γ production. IL-12p70 comprises two subunits, p40 and p35. IL-12p40 is essential for inducing IFN in Natural Killer (NK) and T cells, with its production in DC cells being dependent on IRF8.^171^ IRF8^’^s role in inducing IL-12 extends beyond regulating DC cell development, it also directly participates as a transcriptional activator in the gene transcription regulation of these cytokines.^1^ IRF1 is involved in the induction of IL-12p40 in DC cells and is necessary for macrophages to fully induce IL-12p40.^27,160,172^ Additionally, IRF5 is involved in activities that include the induction of pro-inflammatory factors such as IL-12p40, TNF-α, and IL-6. This process is mediated by TLR signaling, which stimulates IRF5’s translocation to the nucleus, interaction with MyD88 and TRAF6, and initiation of IL-12p40 expression.^151^

In conclusion, the IRF protein family is integral to the development of DCs, and the induction of IFN-I within these cells. The production of IFN in response to pathogen infection is crucial for activating effective innate immunity to combat infection before the establishment of adaptive immunity. IRFs endow DCs with the necessary versatility for the optimal regulation of immune responses.

### The role of IRFs in myeloid differentiation and myeloid derived inhibitory cells (MDSCs) aggregation

#### The differentiation of macrophages and granulocytes is regulated by IRFs

The Fig. 2c reveals the role of IRFs in macrophages and granulocytes differentiation. IRF8 is predominantly localized within hematopoietic cells and is variably expressed throughout the differentiation, proliferation, and apoptotic processes during myeloid cell development, where it orchestrates their core functions.^169^ The differentiation of myeloid progenitor cells yields granulocytes and macrophages,^173^ with studies on *Irf8*^-/-^ myeloid progenitors revealing that IRF8 directs the trajectory of differentiation, favoring macrophage formation while suppressing granulocyte differentiation. *Irf8*^-/-^ mice manifest symptoms akin to human chronic myeloid leukemia (CML).^174^ Irf8 gene deletion leads to an increased progenitor cell population that is hypersensitive to granulocyte colony-stimulating factor (G-CSF) and granulocyte-macrophage colony-stimulating factor (GM-CSF). These progenitors exhibit a diminished response to macrophage colony-stimulating factor (M-CSF) but retain the capacity to differentiate into granulocytes in the presence of M-CSF.^175^ IRF8 expression is sustained in macrophages but reduced in granulocytes. It governs several pivotal genes implicated in myeloid cell proliferation and apoptosis, inhibiting cell growth and promoting apoptosis.^175,176^ The absence of IRF8 disrupts the myeloid differentiation program, skewing differentiation towards granulocytes and culminating in a CML-like syndrome. Notably, a marked reduction in IRF8 transcription levels has also been noted in cells derived from human CML patients.^177^

Mammalian rapamycin target (mTOR) affects M-CSF receptor CD115 expression by regulating the STAT5-IRF8 axis, controlling the development of monocytes/macrophages in the early stages.^178^ Upon pathogenic stimulation of human blood monocytes, a transcription factor complex comprising IRF8 and PU.1 associate with IFN-β to initiate Ets-IRF complex element (EICE) binding in subregions, recruit IRF3, and swiftly induce IFN-β expression, quickly initiate innate immune response to pathogens.^179^ IRF8 also upregulates multiple genes essential to macrophage function, including those linked to endosomes and lysosomal enzymes (e.g., cathepsin C, lysozyme, cystatin C), as well as genes that stimulate macrophage adhesion and migration (such as the Dab2 gene).^180,181^ IRF8 can form heterogeneous complexes with other transcription factors, such as the ETS family member PU.1 and IRF1, as a co-activator of various IFN-induced genes in macrophages, indirectly modulating the transcription of IFN-γ-responsive genes at the gamma-activated site (GAS), including those encoding IL-12p40, IL-12p35, gp91phox, p67phox, TLR4, TLR9, inducible nitric oxide synthase (iNOS), Fcγ receptor I (FcγRI), IRF8 itself, IL-18, chemokine ligand 5 (CCL5)/RANTES, and the phagolysosomal natural resistance-associated macrophage protein 1 (NRAMP1), thereby bolstering host defenses against pathogens.^27,169,182,183^ IRF8 regulates the nucleotide-binding domain, leucine-rich repeat-containing receptor (NLR) family of apoptosis inhibitory proteins, and nucleotide-binding oligomerization domain-containing (NOD)-like receptor family caspase activation and recruitment domains (CARD) containing 4 (NLRC4) inflammasome activation, eliciting cellular responses to bacterial proteins that lead to caspase-1 activation and subsequent secretion of pro-inflammatory cytokines IL-1β and IL-18, which promote pyroptosis and intracellular pathogen resistance within macrophages.^184^ Additionally, IRF8 can bind to the promoters of autophagy-related genes, encompassing all stages of autophagy in macrophages under conditions such as IFN-γ/TLR stimulation or pathogen infection.^185^ Macrophages possess intrinsic mechanisms to fine-tune IRF8 transcriptional activity, including the deSUMOylation of IRF8 by SUMOs, ubiquitination by ubiquitin ligase TRIM21, and nucleosome remodeling by IRF8, all of which influence the transcriptional output of IRF8 target genes.^135,186^

IRF1 and IRF2 play critical roles in the regulation of myeloid cell differentiation. Myeloid cells deficient in IRF1 exhibit impaired maturation. In macrophages stimulated by IFN, the IFN-inducible transcriptional activator IRF1 targets and modulates the expression of a multitude of genes that contain ISREs. These genes include those encoding guanylate-binding proteins, iNOS, caspase-1, cyclooxygenase-2 (Cox-2), class II transactivator (CIITA), and gp91phox.^1,187–189^ Under inflammatory conditions, IRF1 can also form complexes with IRF8, co-activating IFN-induced gene expression. Conversely, a deficiency in IRF2 results in the expansion of eosinophil populations, leading to elevated IL-4 expression and the promotion of Th2 cell polarization. Macrophages lacking IRF2 (Irf2^-/-^) show increased expression of caspase-1, suggesting that IRF1 and IRF2 have opposing regulatory effects on caspase-1 expression. Despite this, IRF1 and IRF2 demonstrate synergistic effects in the regulation of IL-12p40 and Cox-2 expression.^1^

#### Regulatory functions of IRFs in microglia and osteoclasts

Macrophages are distributed across various tissues and organs, with their classification—including microglia, osteoclasts, and alveolar macrophages (AMs)-based on location and function.^190,191^ Microglia, the resident macrophages of the central nervous system, are integral to the glial system, contributing to neural homeostasis and protection.^192,193^ The Fig. 2c reveals the role of IRFs in microglia differentiation. Within microglia, IRF8 establishes a positive feedback loop with the transcription factor PU.1, sustaining IRF8 expression.^194^ Following neural injury, IRF8 activates target genes that transition microglia to reactive phenotypes, which can have protective or neurotoxic effects on the nervous system.^195,196^ In reactive microglia, IRF1 collaborates with IRF8 to initiate transcription of IL-1β.^197^ The absence of Irf8 in microglia can lead to an increase in TNF-α, mediate an increase in hippocampal nerve excitability, and drive fatal epilepsy.^198^

Studies have identified IRF7 as a factor that promotes glioblastoma multiforme progression. IRF7 regulates the expression of inflammatory cytokines, polarizes microglia towards an M2 phenotype, fosters an immunosuppressive tumor microenvironment, and facilitates tumor growth, invasion, and immune evasion.^164^

Osteoclasts, specialized terminal differentiation cells within the monocyte-macrophage lineage, fuse with monocytes to form multinucleated giant cells involved in bone resorption.^199^ The Fig. 2c presents the role of IRFs in osteoclast differentiation. IRF8 acts as a negative regulator in osteoclast differentiation and maturation.^200^ The absence of IRF8 leads to an increase in osteoclasts, contributing to bone metabolic disorders and playing a pivotal role in osteoclast-related inflammatory diseases such as multiple root resorption.^201–204^

#### The regulatory role of IRFs in MDSCs

MDSCs represent a heterogeneous set of myeloid progenitor cells, including precursors unable to differentiate into macrophages, granulocytes, and DCs.^205^ These progenitor cells can become arrested at various stages of maturation due to influences such as tumors, inflammation, trauma, or certain autoimmune diseases, resulting in a population with potent immunosuppressive capabilities.^206^ MDSCs are a hallmark of malignancy and a promising target for cancer treatment.^207^

The regulatory role of IRFs in MDSCs is shown in the Fig. 2c. IRF8 is a critical factor in myeloid cell development and acts as a negative regulator of MDSCs.^208^ Within the tumor microenvironment, tumor cells and infiltrating stromal cells release cytokines like G-CSF and GM-CSF, which activate the Janus kinase (JAK)/STAT signaling pathway and suppress IRF8 expression in MDSCs. This suppression of IRF8 hinders T cell activation and infiltration, thereby diminishing the anti-tumor immune response.^209^ In murine models, overexpression of IRF8 has been shown to restrict MDSC accumulation, mitigate the immunosuppression exerted by MDSCs, counteract their tumor-promoting effects, and improve the effectiveness of immunotherapies.^208^

IRF8 also interacts with the promoter of the Bax gene, influencing the Bax-mediated apoptotic pathway and promoting apoptosis of MDSCs.^210^ Additionally, the expression of IRF4 within MDSCs is reduced,^211^ and IRF4 deficiency contributes to MDSC proliferation in the tumor microenvironment,^212^ Enhancing IRF4 expression can decrease MDSC numbers and attenuate their immunosuppressive function by inducing differentiation.^213^ Besides, as a tumor suppressor, IRF7 is known to downregulate the expression of S100A9, a protein that inhibits the expansion and aggregation of granulocytic MDSCs, thereby curtailing tumor cell metastasis and dissemination.^214^

#### Regulation of NK Cells development by IRFs

IRF1 exerts a selective impact on the stromal cells within the developmental niche of NK cells (Fig. 2d).^215^ IRF1 is implicated in regulating the expression of IL-15 in these stromal cells, a cytokine that is essential for NK cell development.^1^ Consequently, in IRF1 knockout (Irf1^-/-^) mice, a significant reduction in NK cell populations is observed, accompanied by the abrogation of NK cell functions, including cytotoxicity and IFN-γ secretion, particularly following IL-12 stimulation.

Similarly, the absence of IRF2 also impairs the development and functionality of NK cells (Fig. 2d). However, the underlying mechanism appears to differ from that of IRF1, as it may involve the promotion of accelerated apoptosis in NK cells.^216^ This suggests that while both IRF1 and IRF2 are integral to NK cell biology, they contribute through distinct pathways, with IRF1 primarily influencing the cytokine milieu necessary for NK cell maturation, and IRF2 affecting NK cell survival. In a study on prostate cancer, researchers found that overexpression of IRF7 can increase IFN-β, significantly enhance NK cell activity, and limit bone metastasis of prostate cancer cells (Fig. 2d).^164^

#### Multistep regulation of B cells and plasma cells by IRFs

IRF4 is pivotal in B cell ontogeny, encompassing pre-B cell differentiation, receptor editing, germinal center reactions, and plasma cell formation.^217^ IRF4 expression is characteristic of immature B cells and is subsequently downregulated as antigen-activated B cells transition into the germinal center. Within the germinal center, IRF4 protein is scarcely detectable in the majority of B cells, including both centripetal and most centroblasts.^218^ The Fig. 2e shows the regulation of B cell differentiation by IRFs.

Both IRF4 and IRF8 are co-expressed in immature B cells, where they collaboratively modulate B cell differentiation.^1^ Although their regulatory roles appear to be overlapping, compelling evidence indicates that a block in B cell development occurs exclusively in mice lacking both IRF4 and IRF8, manifesting as a pre-B cell stage arrest.^219^ In mice deficient in either Irf4 or Irf8 alone, B cell maturation arrest can be compensated for by the normal expression of the other factor. The enhancer of the conventional immunoglobulin light chain gene contains an EICE, where IRF4 and IRF8 interact with ETS transcription factors such as PU.1 or SpiB.^217^

Upon antigen stimulation, B cells migrate to the germinal center within lymphoid tissues, where IRF4 expression is suppressed and IRF8 expression is augmented in the germinal center’s dark zone.^220,221^ In the light zone, centrocytes undergo affinity maturation and isotype switching to evade apoptosis and differentiate into plasma cells that secrete high-affinity antibodies. Here, IRF8 expression diminishes while IRF4 expression escalates.^222,223^ In the dark zone germinal center response, IRF8 critically regulates genes such as activation-induced cytidine deaminase (AICDA or AID) and B cell leukemia/lymphoma 6 (BCL6).^224^ The AICDA gene encodes AID protein, a crucial enzyme in the processes of DNA strand unwinding and recombination, governing somatic hypermutation and class-switch recombination, leading to diverse and highly specific antibody generation. BCL6 is a transcriptional repressor that orchestrates the germinal center response. IRF4 can regulate the Aicda and PR/SET domain 1 (Prdm1) genes, where low levels of IRF4 can stimulate Aicda, facilitating AID function, followed by an upsurge in IRF4 expression. Elevated levels of IRF4 induce Prdm1 expression, which in turn represses Bcl6 and Aicda through transcriptional inhibition, culminating in the maturation and differentiation of plasma cells.^222^

#### Thymocyte development and Th1/Th2 differentiation regulated by IRFs

T cells orchestrate cellular immune responses within the body, possessing the ability to directly eliminate target cells or secrete cytokines to amplify immune responses. Additionally, they play a role in modulating B cell-mediated humoral immunity. T cells constitute a diverse and heterogeneous group, with various functional subsets at different stages of development. Based on their roles in immune responses, T cells can be categorized into Th, effector T cells (Te), cytotoxic T lymphocytes (CTLs), Tregs, memory T cells, and inhibitory T cells, among others.^225^ Dysregulation of T cell subsets is commonly observed in immune pathologies. IRF family members have been implicated in the regulation of T cell development and differentiation (Fig. 2f). Activated naive CD8^+^ T cells can differentiate into cytotoxic cells, secreting various cytokines and categorized into Tc1 (also known as CTL), Tc2, Tc17, Tc9, and Tc22 subsets.^226^ Most CD8^+^ T cells differentiate into Tc1, which is stimulated by IL-12 and IL-2 to secrete cytokines such as IFN-γ and TNF-α, directly targeting and killing cells. Tc9 and Tc17 secrete IL-9 and IL-17, respectively.

IRF4 is significant for the differentiation identity of these CD8^+^ T cell subsets. Although not necessary for CTL activation and proliferation, IRF4 is crucial for maintaining and functioning their phenotype. *Irf4*^-/-^ mice display functional defects in effector CTLs, with IRF4 influencing the upstream effector functions of transcription factors T-bet, BLIMP-1 (also known as PRDM1), and the formation of memory CTLs. Tc9 cells, akin to Th9 cells, produce IL-9 and IL-10. The molecular mechanism by which IRF4 affects Tc9 development parallels that in Th9, regulating IL-9 expression via binding to its promoter.^227^ Tc17 cells express IL-17, and *Irf4*^-/-^ mice also exhibit defects in Tc17 differentiation.^228,229^

IRF1 is pivotal in CD8^+^ T cell development, regulating the expression of genes essential for lineage selection in developing thymic CD8^+^ T cells (Fig. 2f). It also influences the activation of CTLs.^230^ Moreover, the activation of CTLs is positively regulated by both IRF4 and IRF8.^212^ While IRF2 also contributes to CTL activity, its absence does not significantly impair CTL function. Unique among its family, IRF2 is a negative regulator that modulates immune responses, maintaining balance in the IFN-I signaling pathway and preventing CD8^+^ T cells from overreacting to antigenic stimulation, which could otherwise lead to detrimental effects on the body.^231^

IRF1 is not necessary for Th1 differentiation of CD4^+^ T cells; however, its absence can lead to developmental defects in various cell types, ultimately constraining Th1 differentiation.^232^ Mice deficient in IRF1 are devoid of NK cells, which are critical for secreting IFN-γ that in turn stimulates macrophages to produce IL-12, a cytokine indispensable for Th1 differentiation. Additionally, CD4^+^ T cells with IRF1 deficiency exhibit a delayed response to IL-12.^232^ Mice lacking IRF2 also present with differentiation impairments in both NK and Th1 cells, with IRF1 and IRF2 acting together to enhance the expression of IL-12p40. Furthermore, IRF2 serves to restrict the proliferation of eosinophils that secrete the Th2 cytokine IL-4, thereby attenuating Th2 cell polarization.^233^

IRF4 expression in mature T cells does not influence thymocyte development; however, it does affect cytokine production and the cytotoxic capabilities of T cells, potentially impacting their apoptotic and proliferative capacities.^23^ IRF4 is indispensable for the differentiation of CD4^+^ T cells into Th2 cells, while IRF8 exerts an antagonistic regulatory effect.^45^ The absence of IRF8 leads to a deficiency of macrophages and, as well as a compromised Th1 response due to its essential role in IL-12 gene expression. Together, IRF4 and IRF8 orchestrate the Th1/Th2 balance, influencing the generation of plasma cells that secrete highly specific antibodies.^154^ They are integral to the induction of both cellular and humoral immunity, underscoring their significance in the immune response.

Th1 cells are integral to the host defense mechanism against viruses, bacteria, and intracellular pathogens, producing cytokines such as GM-CSF, IL-2, TNF-β, and IFN-γ.^234^ IRF5 is implicated in directing T cell differentiation towards the Th1 lineage, with a reduction in the Th1 subset observed in mice with systemic Irf5 knockout.^235^ IFN-γ stimulation of CD4^+^ T cells activates STAT1, leading to its nuclear translocation and then production of the transcription factor T-bet. IRF5 enhances T-bet’s IFN-γ production at the encoding gene locus, mediates chromatin remodeling, and together with STAT4, induces IFN-γ expression, driving Th1 polarization. A deficiency in IRF5 results in decreased efficiency of Th1 polarization initiated by T-bet.^236^

Th2 cells are involved in the immune response against parasitic infections, allergies, and chronic inflammation.^237^ The differentiation mechanism of Th2 cells is not completely understood; however, IRF4 is a crucial factor in the development of the Th2 subset and acts as an inhibitor of IRF5 transcription.^238,239^ IL-4 is a critical cytokine for Th2 polarization. Antigen stimulation leads to the upregulation of IRF4, while IL-4 induces the phosphorylation and nuclear translocation of STAT6. Phosphorylated STAT6, in conjunction with IRF4, activates the transcription factor GATA binding protein 3 (GATA3). Transcription factors IKAROS family zinc finger 1 (Ikaros or IKZF1) and GATA3 can promote the transcription of Th2 polarization cytokines IL-4, IL-5, and IL-13.^240–242^ Within these regulatory mechanisms, IRF5 negatively regulates Ikaros transcription, whereas IRF4 antagonizes the MyD88-IRF5 interaction, inhibiting IRF5 activation.^243^ A positive feedback loop exists between IRF4 and GATA3, and together with Ikaros, they can suppress the Th1 transcriptional network, ultimately reinforcing the Th2 phenotype.

Th9 cells, characterized by their secretion of IL-9 and IL-10, play a significant role in combating extracellular parasites.^244^ IL-9 production by Th9 cells is highly dependent on IRF4, which activates the IL-9 gene promoter and regulates IL-9 expression.^245,246^ IRF4-deficient T cells are hindered in their differentiation into Th9 cells. IRF4 collaborates with various interacting proteins, such as Basic Leucine Zipper ATF-Like Transcription Factor (BATF), EICEs, and SMAD2/3, to regulate Th9 cell differentiation.^244^

The dysregulation of Th17 cells is closely associated with muptiple diseases, such as psoriasis, inflammatory bowel disease (IBD), and cancers, making them therapeutic targets for regulating immune dysfunctions.^247^ Th17 and Treg cells both originate from CD4^+^ naive T cells. Transforming growth factor β (TGF-β) induces naive T cells to differentiate into immunosuppressive Treg cells, while in combination with pro-inflammatory factors like IL-6 and IL-21, it drives the differentiation into immune-promoting Th17 cells.^248^ An imbalance between Th17 and Treg cells can lead to autoimmune and/or inflammatory diseases. The interplay between IRF4, Th17, and Treg cells is complex. IRF4 deficiency can confer resistance to Th17-related autoimmune diseases due to defects in Th17 cell differentiation.^247,249,250^ While IRF4 is not essential for Treg production, it is associated with Treg effector functions.^244^ Treg cells express high levels of IRF4, which can inhibit Th2 cell activity by regulating specific transcriptional programs within Treg cells.^251^ IRF5 appears to act as a positive regulator in Th17 differentiation. IL-6 stimulates the phosphorylation and nuclear translocation of STAT3 in naive CD4^+^ T cells, inducing the key transcription factor RAR-related orphan receptor γt expression, which in turn induces IL-17A transcription and drives the Th17-mediated inflammatory response.^247,251^ IRF5 upregulates IL-6 and STAT3, and inhibits Ikaros and IL-10, promoting the Th17 inflammatory response, while IRF4 negatively regulates this effect of IRF5.^236^

Follicular helper T cells (Tfh) are a subset of CD4^+^ Th that are involved in the humoral response, present in secondary lymphoid tissues such as the tonsils, spleen, and lymph nodes.^252^ Tfh cells are distinguished by their expression of C-X-C chemokine receptor 5 (CXCR5), and programmed cell death protein 1 (PD-1), and they facilitate germinal center B cells differentiation into antibody-secreting plasma cells and memory B cells.^253^ A deficiency in IRF4 can result in diminished Tfh cell differentiation. IL-21 is a pivotal cytokine in Tfh development, with IRF4 regulating its production and synergizing with the transcription factor STAT3 to control IL-21 signaling.^254^ IRF4 also cooperates with BATF and BCL6 to regulate Tfh development.^255,256^

### IRFs regulate cell cycle and apoptosis

IRFs are not only integral to the differentiation and development of immune cells but also exert a key role in modulating cell cycle progression and apoptosis. The majority of studies concerning the influence of IRFs on cell cycle and apoptosis have been centered on their role in modulating tumorigenesis. For instance, IRF1 is known to induce the expression of cell cycle inhibitors such as p21 and growth arrest and DNA damage-inducible 45 (GADD45), effectively halting cell cycle progression. In contrast, IRF2 has been shown to facilitate cell cycle processes, acting in opposition to the effects of IRF1. Additionally, IRF3 can directly bind to and activate genes associated with the cell cycle, often in concert with IRF4, thus contributing to the proliferation of myeloma cells.^257^ IRF5 promotes apoptosis upon signaling via TNF-related apoptosis-inducing ligand (TRAIL) death receptors. This is due to TRAIL-induced IRF5 phosphorylation and nuclear localization, resulting in the transactivation of key death receptor signaling components.^258^ Multiple studies have demonstrated that IRF5 negatively regulates cell growth and oncogenesis. However, in thyroid malignancies, IRF5 promotes cancer cell proliferation.^259^ IRF6, as a tumor suppressor gene and transcription factor, functions to suppress tumorigenesis in nasopharyngeal carcinoma (NPC), squamous cell carcinomas (SCCs),and renal clear cell carcinoma.^260–262^ In glioma, IRF6 impaired cell proliferation and induced apoptosis by inhibiting pyruvate kinase isozyme type M2 (PKM2) and glucose transporters 1 (GLUT1) transcription.^263^ The regulation of tumor development by IRF family members will be extensively reviewed in later chapters.

Furthermore, certain members of the IRF family are crucial to the apoptosis of non-tumor cells. Research has demonstrated that IFN-γ induces apoptosis in primary cultured hepatocytes, and that the presence of IRF1 is necessary for Fas/CD95-mediated cellular apoptosis. Caspase-1/7/8 and TRAIL are potential target genes of IRF1 in the regulation of apoptosis.

**Fig. 3:**
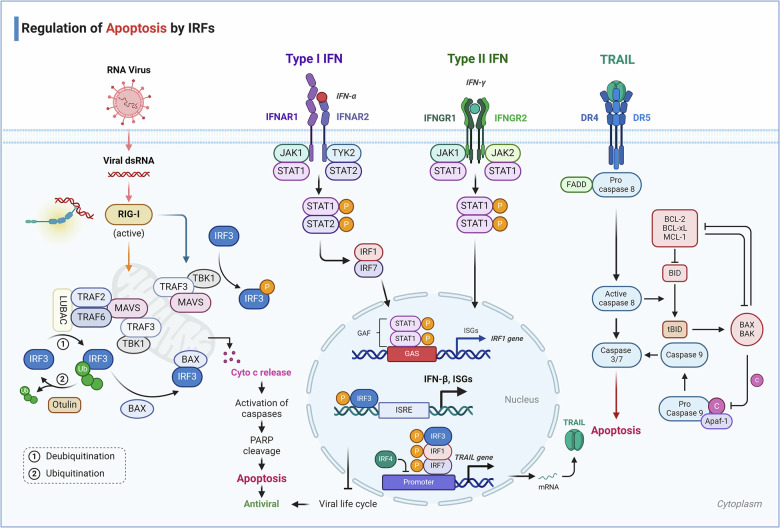
Regulation of apoptosis by IRFs. IRF-1 is a tumor suppressor and a regulatory factor of the IFN-γ system, with IFN-γ promoting the expression of IRF-1. IFN-α also induces the rapid phosphorylation and DNA-binding activity of STAT-1, followed by the accumulation of IRF-1 and IRF7. These two transcription factors bind to the TRAIL promoter and induce TRAIL expression. TRAIL is a key participant in the apoptotic pathway and plays a significant role in IFN-induced cell killing. IRF-3 is also involved in the transcriptional induction of TRAIL, where it transactivates the TRAIL promoter upon viral infection, upregulating TRAIL transcription. Conversely, IRF4 actively inhibits the transactivation mediated by IRF1. IRF3 triggers apoptosis through the RIPA pathway, which depends on the linear ubiquitination of specific lysine residues of IRF3. Within the RIPA signaling pathway, IRF3 interacts with the pro-apoptotic protein Bax to form the IRF3-Bax complex and translocates to the mitochondria, catalyzing the release of cytochrome C into the cytoplasm, subsequently activating Caspase, and ultimately leading to apoptosis. Otulin, a deubiquitinase that removes linear ubiquitin chains, inhibits RIPA by deubiquitinating IRF3 in virus-infected cells

Beyond directly impairing virus replication, another critical host defense strategy against viral propagation involves inducing apoptosis in virus-infected cells. IRF3 has dual functions in such cells: it not only activates antiviral genes but also initiates apoptotic cell death through the RIPA pathway.^264,265^ The activation process of IRF3 in the RIPA pathway is different from its transcriptional activation mechanism, necessitating linear polyubiquitination at particular lysine residues on IRF3 (Fig. 3). In the RIPA signaling pathway, IRF3, upon activation, engages in a critical interaction with the pro-apoptotic protein Bax via the BH3 domain proximal to its C-terminal region. The ensuing IRF3-Bax complex undergoes translocation to the mitochondria, acting as a catalyst for the release of cytochrome C into the cytoplasm.^266^ This event precipitates the subsequent activation of caspases, culminating in apoptosis. However, Otulin, a deubiquitinase that excises linear multiubiquitin chains, suppresses RIPA via deubiquitinating IRF3 in virus-infected cells. To counteract the inhibitory effect of Otulin RIPA facilitates the targeted degradation of Otulin through the continuous action of activated caspase-3 and proteasomes.^95^ Caspase-3 cleaves Otulin at D31, and the fragment was totally degraded by proteasomes before K48-linked ubiquitination occurs.

Moreover, in a mouse model of alcoholic hepatitis, the non-transcriptional activity of IRF3 regulates the live’s innate immune milieu via increasing immune cells’ apoptotic cell death, thereby promoting the resolution of injury.^267^

### IRF1 and IRF3 regulate PANoptosis

**Fig. 4:**
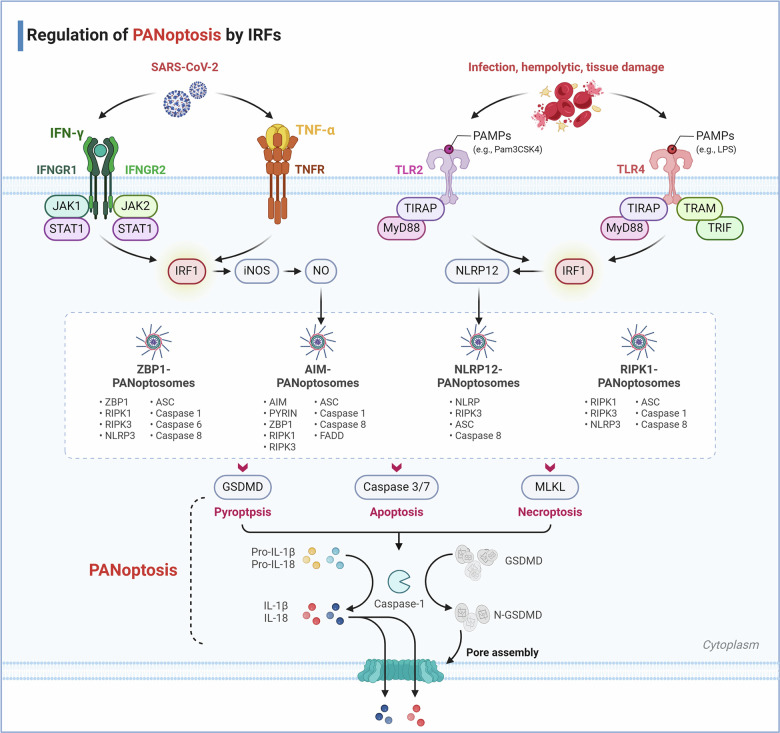
Regulation of PANoptosis by IRFs. IRF1 has been identified as an upstream regulator of PANoptosis, facilitating the activation of PANoptosome and PANoptosis. To date, molecular characterizations have been conducted on four PANoptosome complexes: ZBP1-PANoptosome, AIM2-PANoptosome, RIPK1-PANoptosome, and NLRP12-PANoptosome. During SARS-CoV-2 infection, innate immune cells produce a variety of inflammatory cytokines, among which the combination of TNF-α and IFN-γ induces PANoptosis. Co-treatment with TNF-α and IFN-γ activates the JAK/STAT1/IRF1 axis, inducing the production of NO and driving caspase-8/FADD-mediated PANoptosis, thereby driving cytokine storms and inflammatory pathology. In the context of hemolytic diseases, heme and PAMPs bind to TLR2 and TLR4, inducing signal transduction through IRF1 and upregulating NLRP12 expression. NLRP12, an innate immune cytosolic sensor, is responsible for inflammasome and PANoptosome activation, inflammatory cell death, and the inflammatory response to heme plus PAMPs, driving inflammatory cell death and pathology under hemolytic conditions. The NLRP12-PANoptosome induces the maturation of IL-1β and IL-18 through caspase-8/RIPK3, driving PANoptosis

PANoptosis is a novel and unique inflammatory programmed cell death pathway, uniquely regulated by multifaceted PANoptosome complexes.^268,269^ Previous studies have found that IRF1 is an upstream modulator of PANoptosis, helping to induce the activation of Z-DNA binding protein 1 (ZBP1), NOD-like receptor family, pyrin domain–containing 3 (NLRP3), absent in melanoma-2 (AIM2), RIPK1, NLRP12 associated PANoptosis (Fig. 4).^268,270–272^

IRF1 is involved in the regulation of PANoptosis not only in inflammatory diseases but also in tumors, especially colorectal cancer. During influenza A virus (IAV) infection, ZBP1 protein expression, NLRP3 inflammasome, caspase-1, caspase-8, caspase-3, and mixed lineage kinase domain like pseudokinase (MLKL) activation were decreased in IRF1-defective cells. Further studies demonstrated that IRF1 is a transcriptional regulator of ZBP1.^271^ Genes encoding IRF1, IRF5, and IRF7 are highly upregulated in patients with severe coronavirus disease-2019 (COVID-19), as well as in TNF-α and IFN-γ-treated bone-marrow-derived myeloid cells. Notably, IRF1-deficient cells exhibit resistance to cell death following TNF-α and IFN-γ exposure, a protective effect not observed in cells lacking IRF5 or IRF7.^273^ Concurrent treatment with TNF-α and IFN-γ triggers the activation of the JAK/STAT1/IRF1 signaling pathway, which in turn stimulates nitric oxide synthesis and promotes caspase-8/FADD-mediated PANoptosis.^273^ The recent study has uncovered that NLRP12 is a cytoplasmic sensor of heme plus pathogen-associated molecular patterns (PAMPs)-triggered PANoptosis, driving inflammasome and PANoptosome activation, cell death, and inflammation.^274^ IRF1 is involved in the TLR2/4-mediating signaling pathway to induce Nlrp12 expression, leading to inflammasome assembly and the maturation of pro-inflammatory cytokines IL-1β and IL-18.^274^ However, IRF1 does not influence inflammation and inflammasome activity but instead acts as an upstream regulator of PANoptosis, inducing cell death during colitis-related tumorigenesis.^275^ Recently, Zhuang et al. reported that bile acid-induced phosphorylation of IRF3 mediates cell apoptosis through the regulation of ZBP1 gene expression in cholestasis-induced liver and kidney injury.^276^

### IRF6 regulates Keratinocyte development

IRF6 is essential for normal epidermal development and differentiation. Human mutations in the Irf6 gene underlie two genetic conditions: Van der Woude syndrome and popliteal pterygium syndrome.^277^ Studies have found that mice with null or homozygous missense mutations in the Irf6 gene display a hyperproliferative epidermis incapable of terminal differentiation, leading to abnormal skin, limb, and craniofacial development.^278,279^ Subsequent research has shown that IRF6 is a key target of Notch in keratinocytes and induces differentiation through a Notch-dependent mechanism.^280^ Moreover, IRF6 deficiency results in impairing the expression of genes critical for lipids metabolism and formation of tight junctions.^73^

## Signaling pathways IRFs involved

### IRFs regulate IFN-I via TLR signaling

**Fig. 5:**
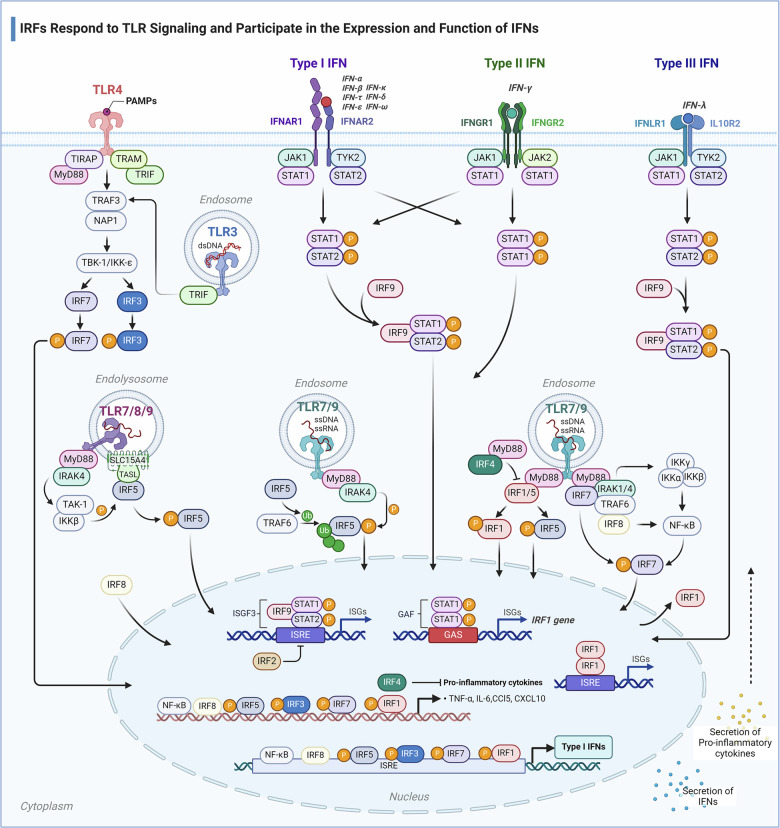
IRFs Respond to TLR Signaling and Participate in the Expression and Function of IFNs. The basal expression of IRF1 maintains the expression of ISGs, thereby conferring antiviral activity. Following viral infection, higher expression of IRF1 is triggered, along with potential cytokines, inducing the expression of IFN and ISGs. Additionally, the secretion of IFN can activate the STAT1-STAT2-IRF9 complex, inducing IRF1 and IRF7 as part of a positive feedback loop. The binding of PAMPs with TLR activates the IRFs signaling pathway, promoting the expression of IFN, pro-inflammatory cytokines, and chemokines, thereby activating the host’s innate immune defense

TLRs are membrane-bound signal receptors identified in the innate system.^281^ Structurally, TLRs consist of an extracellular region, a transmembrane region, and an intracellular region.^282^ So far, 12 functional TLRs have been found in mice and 10 in human.^283^ TLRs are situated on cell membranes or embedded within endosomes. Engagement with their respective homologous ligands prompts TLRs to recruit adaptors, including MyD88 and/or Toll/IL-1 receptor (TIR) domain-containing adaptor protein-including IFN-β (TRIF) to activate IRF proteins alongside other transcription factors (Fig. 5).^160^

Specifically, IRF1 is implicated in the TLR7/9-MyD88 signaling cascade, and both IRF3 and IRF7 are involved in the TLR3-MyD88/TLR4-TRIF/MyD88-TBK1 signaling pathways. Additionally, IRF7 is also recruited to the TLR7/9-MyD88 signaling pathway, but IRF3 is not.^284,285^ IRF5 is involved in TLR7/8/9-MyD88 signaling pathways that lead to the induction of IFN-I genes.

IRF3 and IRF7 can be activated via TLR3/TLR4-TRIF/MyD88-TBK1 signaling pathways. Following ligand recognition, TRIF undergoes phosphorylation and subsequently recruits downstream signaling molecules, including TRAF3, Nucleosome Assembly Protein 1 (NAP1), and TBK1. This cascade facilitates the phosphorylation, dimerization, and nuclear translocation of IRF3 and IRF7 (Fig. 5). Finally induced IFN-I gene expression. Additionally, MyD88/TRAF6-dependent K63-linked ubiquitination of IRF7 is essential for the induction of the IFN-I gene in pDCs by TLR signaling.^285^

IRF5 is also involved in TLR-dependent (TLR7/8/9) induction of IFNs-I.^286,287^ The induction of the IFN-I gene via TLR7 and TLR9 is contingent upon the MyD88 adaptor protein. Activation downstream of the TLRs involves TRAF6-mediated K63-linked poly-ubiquitination as well as phosphorylation in the IAD, which is crucial for both IRF5 nuclear translocation and to dislocate a C-terminal autoinhibitory domain, thereby facilitating the interaction of activated IRF5 with transcriptional coactivators such as CBP/p300.^100,288–291^ Recently studies showed that IRF5 was recruited to endolysosomes by “TLR adaptor interacting with solute carrier family 15 member 4 (SLC15A4) on the lysosome” (TASL) and then phosphorylated by IKKβ or TBK1/IKKε, ultimately inducing IFN-I gene expression(Fig. 5).^292,293^ This mechanism is an analogy with the IRF3 adaptors stimulator of IFN genes (STING), mitochondrial antiviral signaling protein (MAVS) and TRIF.

IRF1 interaction with MyD88 to control the production of TLR9-dependent IFN-β in mouse DCs.^294^ IRF1 also be efficiently activated by the TLR-MyD88 pathway, in turn inducing immunity-related GTPase B10 (IRGB10) expression. Moreover, IRF8 engages in the induction of IFN-I genes in TLR-stimulated pDCs but does not interact with MyD88.^295^ IRF8 in conjunction with TRAF6, modulates TLR signaling, potentially facilitating the interaction between IFN-γ and TLR signal pathways.^296^ In addition, IRF8 is essential for TLR7- and IFN-α-induced bone marrow differentiation into MDSCs in vitro.^297^ IRF8 also directly regulates the expression of TLR9 in NK cells. IRF8 regulates hematopoietic stem cells, at least in part, by controlling TLR9 signaling in various innate immune cells.^298^

### IRFs induce IFNs on innate recognition of cytosolic RNA and DNA

RLRs are RNA sensors localized in the cytosol.^299^ In innate antiviral immunity, except TLR7 and TLR9, the majority of cell types detect viral nucleic acids predominantly via RLRs, thereby initiating antiviral immune responses.^300,301^ RIG-I and melanoma differentiation-associated gene 5 (MDA5) are two RNA helicase enzymes and essential members of the RLR family. RIG-I and MDA5 both contain two CARD at the N-terminus.^300^ The MAVS, which contains one CARD domain, is the adaptor molecule linking the sensing of viral RNA by RIG-I or MDA5 to downstream signaling.^300^ MAVS transmits downstream signaling via CARD-CARD interaction.^160^

**Fig. 6:**
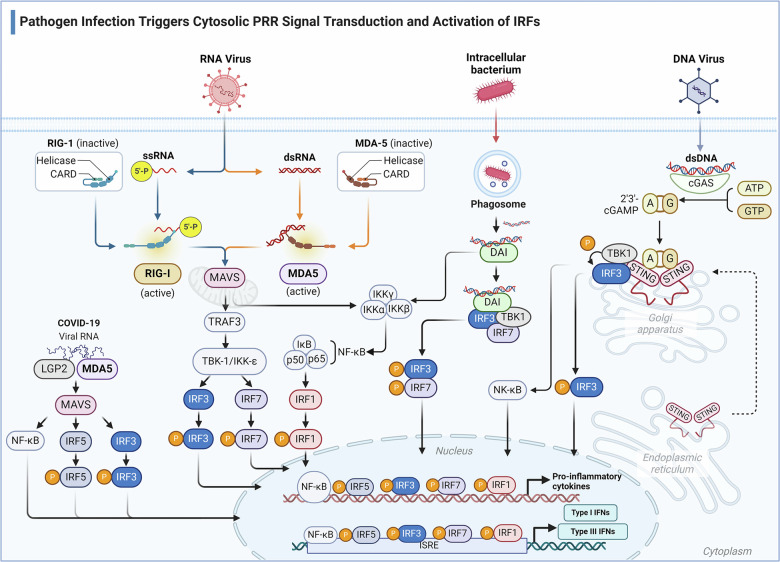
PRRs Detect Cytosolic DNA/RNA and Activation of IRFs.Cytosolic RNA or DNA triggers host responses through specific cellular PRRs. The binding of ssRNA or dsRNA to the helicase domain of RIG-I/MDA5 induces the activation of RIG-I/MDA5’s CARD and the interaction between the CARD-like domain of the adaptor MAVS located on the mitochondrial membrane. This receptor-adaptor interaction leads to the activation of TRAF, TBK1, and IKKε, inducing the phosphorylation of specific serine residues on IRF3 and IRF7. These IRFs then translocate into the nucleus and activate IFN-I genes. NF-κB is also activated and participates in the induction of IFN-I genes. dsDNA is recognized by cGAS, DAI, etc. The adaptor protein STING on the endoplasmic reticulum membrane signals downstream to these DNA receptors, recruiting TBK1 to phosphorylate IRF3, leading to the activation of IFN-I gene expression

IRF3 and IRF7 are critical for the RIG-I/MDA5-mediated IFN-I gene-induction pathway (Fig. 6). Under basal conditions, IRF3 and IRF7 exist in inactive states within the cytoplasm of uninfected cells. After virus infection, TBK1, activated by RIG-I- or MDA5, phosphorylates IRF3 at multiple residues including Ser396, Ser398, Ser402, Ser404, and Ser405 within site 2, which alleviates autoinhibition and allows IRF3 nuclear translocation and interaction with the coactivator CBP.^302–304^ Then, CBP promotes phosphorylation at Ser385 or Ser386 at site 1 within the regulatory region, permitting IRF3 dimerization.^303^ Presumably, a similar mechanism occurs in IRF7, leading to a holocomplex containing dimerized IRF3 and IRF7, either as a homodimer or heterodimer, and coactivators such as CBP or p300 are formed in the nucleus. These holocomplexes bind to target ISRE DNA sequences within the promoters of IFN-I and IFN-III genes.

Additionally, IRF5 participates in the RIG-I/MAVS signaling pathway.^167^ Upon infection with vesicular stomatitis virus (VSV) or NDV, IRF5 undergoes translocation from the cytoplasm to the nucleus. However, detection of severe acute respiratory syndrome coronavirus 2 (SARS-CoV-2) by lung epithelial cells requires MDA5, laboratory of genetics and physiology 2 (LGP2), and NOD1, but not RIG-I (Fig. 6).^305^ Subsequently, IRF3, IRF5, and nuclear factor kappaB (NF-κB) are phosphorylated and translocate into the nucleus to trigger IFN induction. During SARS-CoV-2 infection, IRF3, IRF5, and NF-κB/p65 are the key transcription factors regulating the IFN response.^305^

IRF8 is also needed for IFN-I induction in virus-stimulated DCs, which helps to prolong the recruitment of basal transcription machinery to the IFN promoters, a role not shared by IRF7 or IRF3. This supports characteristically high IFN transcription in DCs.^295^ Moreover, IRF8 and PU.1 collaborate to establish a scaffold complex at the IFN-β promoter, thereby enhancing the recruitment of IRF3 and enabling rapid IFN-β transcription in monocytes.^179^

Although IRF1 is not critical for the induction of the IFN-I gene by viruses, its expression is usually typically upregulated swiftly after viral infection or poly(I: C) stimulation.^306^ However, human IRF1 is vital for IFN-γ and STAT1-dependent immunity to mycobacteria.^307^

Beyond mechanisms for sensing cytoplasmic RNA-, cytoplasmic DNA-sensing systems have also been mentioned (Fig. 6). Known DNA sensors include cyclic CMP-AMP synthase (cGAS), IFN inducible protein 16 (IFI16)/IFN-activated gene box polypeptide 41 (DDX41), IFI204, and (Asp-Glu-Ala-Asp) (DEAD). IRF3 is involved in an antiviral response triggered by double-stranded B-form DNA (B-DNA), and this signaling pathway requires the kinases TBK1 and IKKi.^308^ ZBP1 is a candidate cytosolic DNA sensor. ZBP1 binds to double-stranded DNA (dsDNA) and, by doing so, enhances its association with IRF3 and TBK1.^309^ A more recent study indicates that ZBP1 stabilizes Z-form mitochondrial DNA (mtDNA) and facilitates the formation of a cytosolic complex comprising cGAS, RIPK1, and RIPK3 to engage robust IFN-I signaling.^310^

### IRFs involved in the JAK/STAT signaling pathway

The JAK/STAT pathway is a critical cascade in cellular signaling that transmits information from chemical signals outside of a cell to the cell nucleus, resulting in DNA transcription and subsequent gene expression. The interaction between IRFs and the JAK/STAT pathway is not unidirectional. For instance, STAT1 can enhance the expression of IRF1, creating a positive feedback loop that amplifies the immune response.^307^ Upon viral infection or recognition of PAMPs, IRF3, IRF5, and IRF7 are activated. These IRFs then promote the transcription of IFNs-I (IFN-α and IFN-β) and IFNs-III (Fig. 5). The secreted IFNs engage with their corresponding receptors on cell surfaces, leading to the activation of JAKs. Activated JAKs then phosphorylate the STAT1 and STAT2 leading to the formulation of ISGF3, or IFN-γ-activated factor (GAF). Subsequently, ISGF3 or GAF translocate into the nucleus, where they initiate the transcription of ISGs or GAS respectively and induce IRF1 expression in a positive feedback mechanism, orchestrating a robust antiviral response.^307^ The IRF9, as part of the ISGF3 complex (together with STAT1 and STAT2), directly binds to the ISREs in the promoters of ISGs, enhancing their transcription.

IFN-II (IFN-γ) binds to cell-surface receptors composed of IFN-γ receptor 1 (IFNGR1) and IFNGR2 subunits, which bind to JAK1 and JAK2, respectively. Activation of these kinases leads to STAT1 homodimerization to formulate GAF, which translocates into nucleus to target GAS DNA sequences and induce IRF1 transcription (Fig. 5).^160^ In humans, IRF1 is required for the IFN-γ-dependent immunity of macrophage against mycobacteria, whereas its role in IFN-α/β-dependent antiviral immunity is comparatively less critical.^307^

### IRFs involved in the NF-κB signaling pathway

The NF-κB pathway is a key signaling mechanism that controls the transcription of genes involved in immune and inflammatory responses, cell proliferation, and survival. Activation of NF-κB is tightly regulated and can be triggered by various stimuli, including pro-inflammatory cytokines, bacterial or viral infections, and other stress signals. Some IRF members have been shown to interact with the NF-κB pathway, influencing its activity in several ways.

IRF3, IRF5, and IRF7 can function cooperatively with NF-κB to enhance the transcription of certain genes implicated in the immune response. For example, upon viral infection, IRF3, IRF5, and NF-κB can be simultaneously activated and work together to promote the transcription of IFNs-I and other pro-inflammatory cytokines, amplifying the immune response (Fig. 5).^305^ IRFs can also modulate the activation of NF-κB. For instance, IRF4 has been reported to interact with the NF-κB subunit RelA/p65, affecting its ability to bind DNA and activate transcription. This interaction can either enhance or suppress NF-κB target gene expression, depending on the cellular environment and the specific stimuli.

## The role of IRF proteins family in health and diseases

### IRFs and infections

**Fig. 7:**
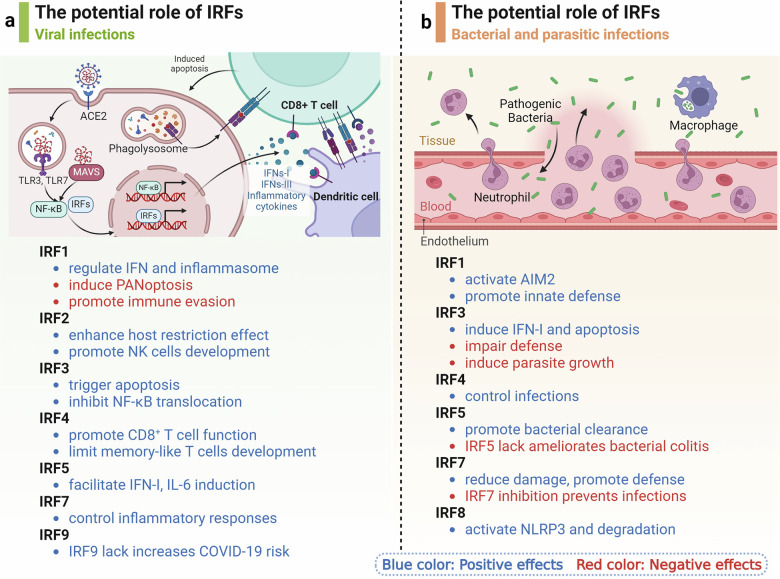
The potential role of IRFs in infectious diseases. **a** The roles of IRFs in viral infections. IRF1 can regulate IFN and inflammasome to fight viral infections, while it also induces cell PANoptosis and virus immune evasion. IRF2, IRF3, IRF4,IRF5, IRF7, and IRF9 play protective roles to prevent viral infections. **b** The roles of IRFs in bacterial and parasitic infections. IRF1, IRF4, and IRF8 prevent host aginst bacterial infections. IRF3, IRF5, and IRF7 are double-edged swords in bacterial and parasitic infections

Almost all IRFs have been reported to affect pathogenic processes (Fig. 7). Generally, IRF1, IRF3, IRF5, and IRF7 are the main members involved in various microbial infections and microbial-induced human diseases, playing strong roles in IFN-I gene regulation at the early phase of antiviral innate immunity and antibacterial pathogens in most cells.^32,63,311,312^ Some studies indicate that activation of certain IRFs can also cause excessive inflammatory reactions and body damage,^313–315^ and inhibition of IRFs may offer protection against microbial infections in some certain specific situations.^316^

#### Viral infections

Following viral infection, IRFs like IRF1 and IRF3 are dramatically upregulated and activated, inducing endogenous IFN genes such as IFN-α and IFN-β1 production, and/or protein degradation in various cell types, which drive antiviral immunity to multiple clinically important viruses, including hepatitis C virus (HCV), West Nile virus,yellow fever virus, human immunodeficiency virus type 1(HIV1), gamma herpes virus, and human metapneumovirus, and VSV infection.^32,165,168,317–320^ IRF1 basal expression provides cellular intrinsic antiviral protection against diverse pathogenic RNA viruses, such as HAV, HCV, dengue virus, and zika virus (ZIKV).^321^ IRF1 can regulate ZBP1 and NLRP3 inflammasome, mediating inflammatory and cell death responses in IAV infection.^271^ In addition, IRF1 is required for optimal early activation of IRF3, targeting and augmenting IRF3 phosphorylation, which enhances innate immunity to viral infection.^168,322^ IRF2 is also identified to inhibit ZIKV and orthopoxvirus replication by enhancing FAM111 trypsin like peptidase A (FAM111A) expression to promote host restriction effect of replication factor C subunit 3 (RFC3).^323,324^ Moreover, IRF2 is critical for the development and functional maturation of human NK cells to defend against virus-infected cells.^325^ IRF3 has been reported to trigger apoptosis of virus-infected cells, restricting virus spread within the host, in a RIPA pathway,^95,264,326^ and IRF3 inhibits NF-κB nuclear translocation to prevent viral inflammation.^327^ IRF3 and IRF7 are vital for inflammatory responses control during HSV-induced encephalitis (HSE).^328^ Studies presented that IRF3 and IRF7 deficiency might increase susceptibility to HSE and other respiratory viruses in humans,^329,330^ and IRF7/9 variants might predispose humans to recurrent, severe HSV or IAV infection.^331–333^ Besides, IRF3 agonist could alleviate Enterovirus A71-induced inflammatory response in mice, indicating a potential therapeutic target in Enterovirus A71-induced hand-foot-and-mouth disease.^334^ IRF4 is able to promote CD8^+^ T cell function and exhaustion, limiting memory-like T cells development during lymphocytic choriomeningitis virus (LCMV)-induced infection.^335,336^ IRF5 is critical for IFN-I and IL-6 induction followed by viral infections,^337^ and it suppresses HCV replication and induces immune responses in the draining lymph node to defeat West Nile virus infection.^312,338^ However, in some conditionsmany viruses, including human cytomegalovirus, influenza virus, rhinovirus, EBV, HCV, HIV, and SARS-CoV, can exploit host RNA-binding protein to inhibit expression and functions of IRF1/3/5, thereby preventing a cellular antiviral response.^326,339–341^ Moreover, virus-induced damage and inflammation can activate TLR-IRF pathways, resulting in excessive inflammatory cytokines production and aggravating body damage.^313,314^

COVID-19, caused by SARS-CoV-2, mainly infects human epithelial cells in the respiratory tract and intestine. Research indicates that patients with SARS-CoV-2 infection have high progesterone levels to facilitate IRF3 phosphorylation, which is associated with decreased severity of COVID-19.^342^ Nonetheless, SARS-CoV-2 can suppress the IFN-mediated STAT1/2 signaling, diminishing NLRC5, IRF1 and IRF3 gene expression.^343–345^ Furthermore, SARS-CoV-2 papain-like protease and nonstructural proteins (NSP) such as NSP5, NSP12, and NSP13 can cleave IRF3, suppress IRF3 phosphorylation and nuclear translocation to blunt IFN response, which further antagonizes host antiviral innate immunity.^346–351^ On the other hand, a study presented that TNF-α and IFN-γ co-treatment caused the JAK/STAT1/IRF1 pathway activation, leading to caspase-8/FADD-mediated PANoptosis, which resulted in a lethal cytokine shock in mice that mirrored inflammation and tissue damage of COVID-19.^273^ Shin et al. uncovered that pathological and/or genetic reasons-mediated higher basal IRF1 expression in multiple organs might contribute to the synergistic upregulation of IRF1 under SARS-CoV-2 infection, potentially making individuals more vulnerable to COVID-19.^352^ Furthermore, Huang et al. illustrated that SARS-CoV-2 activated NF-κB/IRF1 axis to further promote programmed death-ligand 1 (PD-L1) expression and then might facilitate immune evasion, inducing sepsis and fatality.^353^ Zhang et al. reported that inherent defects in the TLR3- and IRF7-dependent IFN-I immunity may encounter with life-threatening COVID-19 pneumonia in patients without a history of severe infection.^354^ Besides, Lévy et al. reported an unvaccinated child with life-threatening COVID-19 risk because of an inherited IRF9 deficiency.^355^ Moreover, it has been found that SARS-CoV-2 spike transfected cells release a large number of microRNAs-loaded exosomes, which are internalized by human microglia and inhibit USP33 expression and IRF9 levels, mediating central nervous system damage through the hyperactivation of human microglia.^356^

#### Bacterial and parasitic infections

IRF1 is important for IFN-γ-dependent and TNF-α and IL-6 auto-paracrine signaling loop-mediated macrophagic immunity to *mycobacteria*.^307,357^ and IRF1 inhibits mTOR/p70 S6K signaling in macrophages, which facilitates an anti-mycobacterial effect against *tuberculosis* (TB).^358^ Furthermore, IRF1 is required for the AIM2 inflammasome activation in response to *Francisella novicida* (*F. novicida*) infection,^272^ and for intestinal group 3 innate lymphoid cells (ILC3s) to produce lots of the protective effector cytokine IL-22 early in the course of *Citrobacter rodentium*-induced intestinal infection.^359^ IRF1 also contributes to the innate defense of the cornea against *Pseudomonas aeruginosa* (*P. aeruginosa*) infection,^360^ indicating it is crucial for anatomical containment and prevention of pathogen dissemination. Additionally, the STING/TBK1/IRF3 pathway activation is instrumental in stimulating IFN-I production and inducing apoptosis, thereby offering protection against TB during *Mycobacterium bovis* (*B. tuberculosis*) infection.^361,362^ Furthermore, IRF3, IRF5, and IRF7 are key regulators in the IFN-I-mediated response to *mycobacterium tuberculosis* (*M. tuberculosis*).^363,364^ Specifically, IRF7 facilitates the expression of miRNA-31, which significantly reduces lung pathology and bacilli burdens during *M. tuberculosis* infection.^365^ Skjesol et al. found that the Rab11 family interacting protein 2 guided TLR4 sorting adapter translocating chain-associated membrane protein (TRAM) recruitment for orchestrates actin remodeling and IRF3 activation for macrophage phagocytosis of Gram-negative bacteria.^366^ On the other hand, infections caused by bacteria like *Helicobacter pylori* (*H. pylori*) can downregulate IRF3 activation to interrupt STING and RIG-I signaling and then hamper their functions in eliminating bacteria and mobilizing adaptive immune responses.^367^ A separate study revealed that airway acidification activated the IRF3/IFN-β signaling, subsequently compromising the host’s defensive mechanisms against pulmonary infection by *Pseudomonas aeruginosa* (*P. aeruginosa*).^315^ Besides, mice with IRF3 deficiency were protected from the lethal malaria *plasmodium yoelii* infection.^368^ Inflammasome-mediated IRF3 signaling upregulated SOCS1, further inhibiting MyD88-IRF7-mediated IFN-I response in pDCs, inducing parasite fast growth and host death.^368,369^ Studies have shown that IRF4 expression in T cells is necessary for the effective control of *M. tuberculosis* and *Listeria monocytogenes*.^370,371^ IRF5 has been reported to promote intracellular bacterial clearance and the induction of antimicrobial pathways.^372,373^ However, IRF5 deficiency ameliorated immunopathology during *Salmonella Typhimurium* (*S. Typhimurium*) and *Helicobacter hepaticus*-induced colitis in vivo.^372,374^ Moreover, Puthia et al. have illustrated that IRF7 inhibition can be used to prevent and treat bacterial infections other than acute pyelonephritis.^316^ However, Mathy et al. showed that long non-coding RNA (lncRNA) Nostrill might function as a “guide” to promote recruitment of NF-κB p65 to the IRF7 gene promoter, thereby promoting IRF7 mRNA expression and contributing to intestinal epithelial defense against *Cryptosporidium parvum* (*C. parvum*).^375^ IRF8 activated autophagy genes in macrophages and induced subsequent autophagic capturing and degradation of Listeria antigens to clear *Listeria monocytogenes*.^185^ IRF8 also mediated IRF3 phosphorylation and promoted NLRP3 inflammasome activation during infection with Gram-negative bacteria.^376^

#### Fungal infections

There are a small number of studies focused on the possible relationships between IRFs and fungal infections. Both *A.fumigatus* (*Aspergillus fumigatus*) and *C.albicans* (*Candida albicans*) are two major human fungal pathogens. IRF1 induces IRGB10 expression to target the fungal cell wall and cause hyphae damage, modify the *A.fumigatus* surface and inhibit fungal growth.^294^ In addition, IRF1 is a fungal recognition pathway downstream and mice lacking IRF1 are hypersusceptible to systemic *C.albicans* infection.^377^ Besides, *C.albicans* infection triggers the cGAS/STING pathway by promoting TBK1 recruitment and activation to phosphorylate IRF3 and mediate IFN-I responses, which further regulate antifungal innate immune responses.^378,379^ IRF5 is required for Dectin-1-mediated IFN-β production in DCs to defense against *C.albicans* infection.^286^ Moreover, IRF5 induces IL-12 production and subsequently generates IFN-γto resist *C.albicans*, and IRF5 activation blockade significantly increases the susceptibility of Clec2dknockout mice to bloodstream infection with *C.albicans*.^380^ Conversely, IRF7 enhances *C.albicans* infection by regulating CD209 expression and p53-AMPK-mTOR-induced autophagy to inhibit macrophages phagocytosis and killing capacity.^381^

There are also several researches on the relationships between IRFs and *F. monophora* (*Fonsecaea monophora*) infections. For example, *F. monophora* can triggers Dectin-1 and then activates IRF1 to mediate IL-12 production, which is crucial for antifungal defenses.^382^ On the other hand, *F. monophora* has been reported to induce the loss of nuclear IRF1 activity, further blocking IL-12A transcription.^382^ IRF4 is critical for Treg cell localization and IL-17 expression in Th17 cell to protect against the fungal opportunistic pathogens *C.neoformans* (*Cryptococcus neoformans*),^383,384^ and prostaglandin E2 produced by *C.neoformans* can inhibit IRF4 function as well as IL-17 production to impede antifungal immunity in T cells.^384^

### IRFs and cancers

**Fig. 8:**
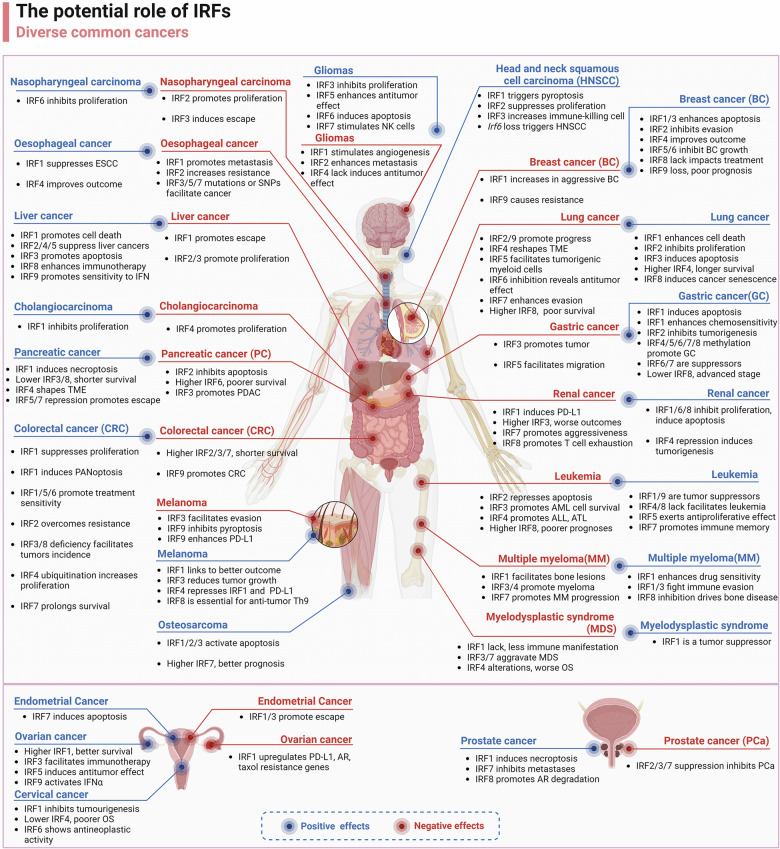
The potential role of IRFs in diverse common cancers. IRF family is involved in almost all human cancers, with protective roles or negative roles, and some IRF members are double-edged swords in cancers development

Numerous studies have illustrated that the IRF family is important for pathogenesis and therapy of various human cancers (Fig. 8). Traditionally, due to their role in inducing IFN-I to mediate tumors immunosurveillance, several IRFs, such as IRF1, IRF3, and IRF7 are regarded as anti-tumorigenic factors. Conversely, IRF2, which is ascribed as a negative regulator of IRF1, has been recognized as a pro-tumorigenic factor and it drives IFN-dependent CD8^+^ T cell exhaustion to suppress anti-tumor immunity.^8,385^ IRF1 and IRF7 knockout mice were shown to have the higher metastasis score than wild-type.^386^ Besides, activation of the IRF1-X-linked inhibitor of apoptosis-associated factor 1 (XAF1) loop significantly promotes stress-induced apoptosis and restricts the invasive capability of tumor cells.^387^ However, both pro-tumor and anti-tumor functions have also been illustrated for different IRF members in human cancers.^388^ It has been reported that IRF1 deficiency in tumor cell presented decreased tumor progression and IRF1 is necessary for PD-L1 upregulation in tumor cells and tumor progression in vivo.^389^ Conversely, IRF2 is a repressor of PD-L1, and loss of IRF2 in cancers can lead to tumor immune evasion.^390^ These diverse findings show a complex role for IRFs members in the inflammatory tumor microenvironment.

#### Oesophageal cancer

Oesophageal or esophageal cancer primarily comprises esophageal SCC (ESCC) and esophageal adenocarcinoma (EA/EAC).^391^ Several IRF family members have been reported to be implicated in oesophageal cancers. IRF1 expression level was downregulated while IRF2 was upregulated in ESCC compared with normal esophageal tissues.^392^ IRF1 suppresses ESCC cell proliferation, while IRF2 acts as an oncoprotein to block the function of IRF1 by preventing its translocation into the nucleus.^392^ Additionally, IRF2 decreased endogenous IFNGR1 levels and the loss of IFNGR1 turned to increase the resistance of esophageal cancer cells to antitumor IFN-γ.^393^ Huang et al. revealed that lncRNA IRF1 antisense RNA (lncRNA IRF1-AS) was downregulated in ESCC tissues, and it could repress ESCC proliferation and promote apoptosis. Furthermore, IRF1 directly binds to IRF1-AS promoter to activate IRF1-AS transcription. Therefore, IRF1-AS inhibits ESCC progression by facilitating IFN response via a positive regulatory loop with IRF1.^394^ Similarly, Watson et al found that IRF1 overexpression inhibited tumor growth in an EA murine model.^395^ However, a recent investigation described that high levels of forkhead box M1 (FOXM1c) and IRF1 were positively associated with low survival rate and indicated a poor prognosis of oesophageal cancer patients.^396^ FOXM1c functions as a proliferation-associated transcription factor that is pivotal in driving tumorigenesis and progression of cancer, and is overexpressed in oesophageal cancer patients.^396,397^ IRF1 is a transcriptional target of FOXM1c and can mediate FOXM1c-related ESCC cell invasion and migration to promote oesophageal cancer metastasis.^396^ IRF3 single nucleotide polymorphisms (SNPs) may relate to esophageal cancer susceptibility among Chinese patients.^398^ In addition, the whole-exome sequencing revealed metastatic ESCCs exhibit frequent mutations in multiple genes, including IRF5.^399^ Additionally, genes like IRF7 may contribute to the susceptibility and/or development of esophageal cancer.^400^ Sun et al. have identified a positive correlation between the IRF4 protein overexpression and improved outcomes within ESCC.^401^

In conclusion, IRF1 serves a dual role in esophageal cancer. IRF1 acts as a tumor suppressor to inhibit esophageal cancer cell proliferation. On the other hand, it functions as a transcriptional target of FOXM1c to mediate FOXM1c-related ESCC cell invasion and migration. In addition, overexpression of IRF2 promotes esophageal cancer cell proliferation and mutations of SNPs in genes such as IRF3, IRF5, and IRF7 may facilitate the progress of esophageal cancers, while IRF4 may be regarded as a protective factor in ESCC.

#### Gastric cancer (GC)

Both IRF1 and IRF2 can restrict GC cell growth and act as protective factors in GC patients. Mutations of the IRF1 gene, inducing the production of functionally impaired IRF1, are critical for GC development.^402^ In fact, IRF1 can induce apoptosis by upregulation of p53 upregulated modulator of apoptosis (PUMA) in GC cells.^403^ In addition, IRF1 overexpression can enhance chemosensitivity to 5-fluorouracil in GC cells.^404^ Recent studies illustrated that IRF1 could suppress DNA damage response (DDR) and reverse chemoresistance by downregulating P-glycoprotein and DNA repair protein RAD51 homolog 1 (RAD51) in GC.^405,406^ Moreover, IRF1 can inhibit GC metastasis via downregulating microRNA-18a (miR-18a)/miR-19a and Wingless-related integration site (Wnt)/β-catenin signaling.^407^ Similarly, IRF2 is also regarded as a tumor suppressor in GC, and its downregulation by miR-18a can enhance GC cell proliferation, migration, and invasion.^408^ Additionally, IRF2 downregulates the expression level of matrix metalloproteinases 1 (MMP1), which promotes GC tumorigenesis, invasion, and metastasis.^409^ Moreover, overexpressed IRF2 positively affects the prognoses of GC patients and inhibits cancer proliferation by directly promoting the tumor suppressor adenomatous polyposis coli membrane recruitment 1 (AMER1) transcription and inhibiting the Wnt/β-catenin signaling pathway.^410^ On the other hand, a study showed that IRF3 was a risk factor for GC, and increased IRF3 level was negatively associated with survival in GC patients.^411^ IRF3 facilitates *H. pylori* and gastric tumor formation through activating Yes-associated protein (YAP), an important downstream transcription coactivator of Hippo pathway to exacerbate tumor progression.^411,412^ IRF3 inhibition by amlexanox impedes gastric tumor growth in a YAP-dependent manner.^411^ Besides, miR-365 inhibited the TLR4/IRF3 axis to reduce IRF3 phosphorylation and YAP expression to restrict gastric precancerous lesions progression.^413^

DNA methylation of IRF4, IRF5, IRF7, and/or IRF8 frequently occurs in GC cell lines and IRF4 methylation is often observed in GC specimens, which may be a useful biomarker for diagnosing the recurrence of GC.^414,415^ Furthermore, treatment with demethylating agents restores the expression of IRF4, IRF5, and IRF8, which may result in antitumor activity.^414^ IRF6 also acts as a tumor suppressor in GC, and its downregulation is clinically correlated with poor prognosis of GC.^416^ Bioinformatics analysis indicates that IRF6 expression is negatively associated with its promoter methylation in GC cohorts from The Cancer Genome Atlas (TCGA).^416^ Although Yamashita et al. illustrated IRF5 may exhibit antitumor activity in GC,^414^ Du et al. indicated that IRF5 was cytoplasm-enriched in GC cells, which promoted GC cell migration by inhibiting expression and inducing degradation of Wnt5a and E-cadherin proteins.^417^ IRF7 is an indirect target of circular RNA circ0007360 to exert tumor-suppressive role and attenuate GC progression.^418^ A single-cell RNA sequencing revealed that IRF8 was decreased in CD8^+^ tumor-infiltrating lymphocytes in GC tissues, and patients with lower IRF8 levels in blood CD8^+^ T cells likely to be at a more advanced GC stage.^419^

Overall, IRF1 expression is beneficial for inhibiting the growth of GC cells through inducing tumor cell apoptosis, enhancing sensitivity to chemotherapy drugs, and suppressing the miR-18a/19a and Wnt/β-catenin pathways. In addition, IRF2 can downregulate MMP1 and Wnt/β-catenin pathway, and promote AMER1 transcription to suppress GC proliferation, invasion, and metastasis. Methylation of IRF4, IRF5, IRF6, IRF7, and IRF8 may be associated with GC progression, and demethylation of these factors may increase antitumor activity. However, cytoplasmic IRF5 also can inhibit Wnt/E-catenin and then facilitate GC migration. Furthermore, IRF3 acts as a tumor promoter by activating YAP to exacerbate GC progression.

#### Liver cancer

Primary liver cancer can mainly be subdivided into hepatocellular carcinoma (HCC), intrahepatic cholangiocarcinoma, and other rare types.^391^ IRF1 in HCC pathogenesis is bidirectional, serving as both a tumor promoter and suppressor. On the one hand, IRF1 upregulates PD-L1 expression, which is beneficial for HCC cells to escape from anti-tumor immunity.^420^ IRF1 can activate mTOR/STAT3/ AKT serine/threonine kinase (AKT) signaling, upregulating Slug, Snail, and Twist to promote HCC cell invasion, migration, and epithelial-mesenchymal transition (EMT).^421^ IRF1 also can activate human endogenous retrovirus-H long terminal repeat-associating 2 (HHLA2) by binding to the promoter region of HHLA2, which induces macrophages M2 polarization and chemotactic migration, leading to HCC immune escape and development.^422^ Studies on the suppression of IRF1 activity may target HCC treatments. For example, the activity of IRF1 can be reversed by IL-33 SUMOylation, resulting in impaired antitumor immunity in HCC cells.^423^ The overexpression of miR-130b and miR-345, which induces the suppression of IRF1, can markedly inhibit HCC cell migration and invasion.^421,424^ However, IRF1 also can promote autophagy, cell death, and inhibit growth in HCC cells and mice.^425,426^ It has been reported that the repression of IRF1 may facilitate the progression from chronic HCV to cirrhosis and HCC.^427^ IRF1 has the tumor-suppressor effect and higher expression of IRF1 is associated with better outcomes in HCC patients.^428,429^ In addition, Yan et al. revealed that IRF1 induces miR-195 to impede checkpoint kinase 1 (CHK1) protein expression, and subsequently upregulates HCC cellular apoptosis, while IRF1 expression or CHK1 inhibition facilitates PD-L1 expression by increased STAT3 phosphorylation.^430^ Furthermore, a recent study discovered that IRF1 positively regulates C-X-C motif chemokine ligand 10 (CXCL10) and CXCR3 to induce CD8^+^ T cells, NK, and NK T cells enrichment and migration, and IFN-γ secretion, causing HCC cells apoptosis, thus overcoming proliferative effects from IRF1-induced PD-L1 expression.^431^ Besides, Studies reported that miR-31, miR-23a, and miR-301a may induce HCC progression via the downregulation of IRF1.^432–434^ Nuclear receptor subfamily 4 group A member 1 (NR4A1) mediates NK cells dysfunction in HCC by inhibiting the IFN-γ/p-STAT1/IRF1 signaling, and NR4A1 deficiency restores the cytotoxicity of NK cells and improves anti-PD-1 therapy efficacy.^435^

Although Guichard et al. once identified that IRF2 might be a tumor suppressor gene in HCC and IRF2 inactivation impaired TP53 function,^436^ other studies revealed that high expressed of IRF2 was relevant to increased recurrence probability in HCC patients and IRF2 downregulation resulted in significantly decreased invasion ability, combined with decreased STAT3, p-STAT3, and MMP9 in HCC cell lines.^428^ IRF2 was found to promote proliferation and inhibit apoptosis of HCC, and increase lenvatinib resistance by upregulating β-catenin, indicating that inhibiting IRF2 is a potential strategy to enhance the therapeutic effect of lenvatinib on HCC.^437^ Moreover, major vault protein induces human double minute 2 to liberate from IRF2, which causes P53 ubiquitination and then promotes HCC development.^438^ In addition, as a competitive antagonist for IRF1, overexpressed IRF2 can downregulate CXCL10 expression via the inhibition of IRF1/CXCL10/CXCR3 axis and decrease PD-L1 expression via the inhibition of IFN-γ/IRF1 to partially inhibit anti-tumor activity of IRF1 in HCC.^420,431^

The role of IRF3 in HCC has also been reported with varying outcomes. Yuan et al. showed that the survival rates in HCC patients improved significantly as a function of increased TLR3 and IRF3 expression levels.^439^ Moreover, IRF3 was positively associated with TLR3 expression in HCC tissues, which may present a synergistic effect on apoptosis and restrain HCC cells proliferation, MMP2 expression, and angiogenesis.^439^ On the other hand, other studies have illustrated that IRF3 has the potential to promote HCC progression. Overexpressed IRF3 was markedly correlated with clinical stages and pathological grades in HCC.^440^ HBV-mediated N6 methyladenosine epitranscriptomic regulation of phosphatase and tensin homolog (PTEN), a well-known tumor suppressor, affects innate immunity by inhibiting IRF3 nuclear import and facilitates HCC development by activating the phosphatidylinositol 3-kinase (PI3K)/AKT pathway.^441^ Ionizing radiation, the main reason for the failure of radiotherapy, can activate the TBK1/IRF3 pathway, upregulating PD-L1 in HCC cells and restricting CTLs activity and protecting HCC cells from immune-mediated eradication.^442^ Disruption of the BRCA1 DNA repair associated-partner and localizer of BRCA2 (PALB2) interaction causes persistent DNA damage in HCC cells, activating the cGAS/STING signaling, which induces PD-L1 expression via the STING/IRF3/STAT1 pathway and then suppresses immune to facilitate HCC tumorigenesis and progression.^443^

Yuan et al. revealed that overexpressed IRF4 restricted HCC cell proliferation and migration capacity by inhibiting the JAK2/STAT3 signaling pathway and EMT.^444^ Methylation arrays revealed that IRF5 and IRF7 were frequently hypermethylated in HCC tissues.^445,446^ Furthermore, IRF5 was downregulated in livers of HCV-positive compared to HCV-negative HCC patients or healthy controls, and IRF5 suppressed HCV replication and HCC pathogenesis.^312^ The expression of IRF8 was also decreased in HCC samples, and IRF8 overexpression in HCC cells significantly improved antitumor effects in immune-competent mice via regulating the recruitment of tumor-associated macrophages (TAMs), inhibiting CCL20 expression, and promoting anti-PD-1 therapy response.^447^ Besides, it has been reported that IRF9 overexpression promoted IFN-induced activity of dsRNA-dependent protein kinase (PKR) in human hepatoma cells.^448^ Reduction in IRF9 levels caused resistance to IFN-α2a treatment in HCC cells,^449^ and another study showed that disease-free survival (DFS) and overall survival (OS) were better in IRF9-positive versus IRF9-negative patients with HBV-related HCC who received postoperative IFN-α treatment, indicating that IRF9 can act as a predictive marker of outcome after postoperative IFN-α therapy in HBV-related HCC patients.^450^

In a word, the roles of IRF1, IRF2, and IRF3 in HCC are complex and bidirectional, exhibiting both tumor-promoting and anti-tumor effects. IRF1 upregulates PD-L1, and HHLA2, and activates the mTOR/STAT3/AKT pathway to facilitate HCC immune escape, invasion, and migration; meanwhile, IRF1 also enhances HCC autophagy and induces miR-195, CXCL10, and CXCR expression to cause HCC cell apoptosis and death. Although IRF2 positively regulates TP53 function to inhibit HCC growth, it also induces β-catenin expression and inhibits IFN-γ/IRF1 and IRF1/CXCL10/CXCR3 axis to suppress HCC apoptosis, enhance HCC proliferation and resistance to therapeutic drugs (Lenvatinib). IRF3 contributes to HCC apoptosis and inhibits HCC proliferation, yet it also causes HCC progression via upregulating PD-L1 expression. IRF4, IRF5, IRF8, and IRF9 act as protective factors to suppress HCC progress.

#### Pancreatic cancer (PC)

PC is mainly classified into exocrine and neuroendocrine neoplasms, of which pancreatic ductal adenocarcinoma (PDAC) accounts for >85% of pancreatic tumors.^451^ IRF1 and IRF2 can regulate PC progression by functioning as an anti-oncoprotein and oncoprotein, respectively.^452^ Additionally, IRF1 facilitates 2'3’-cGAMP/BV6/zVAD.fmk-induced necroptosis in PC.^453^ Furthermore, the zinc finger protein ZBED2, essential for PDAC progression, can antagonize IRF1-induced transcriptional activation and suppress the pancreatic progenitor transcriptional program, promotemotility, and enhance invasion in PDAC cells.^454^ These studies illustrate that IRF1 is a tumor suppressor in PC. Instead, IRF2 promotes PC tumorigenesis, and Cui et al. revealed that IRF2 was upregulated in primary PC tissues and was associated with tumor differentiation, size, tumor-node-metastasis stage, and survival of PC patients.^455^ IRF2 inhibits apoptosis effectors to facilitate PC cell growth.^455^ PC patients with high IRF2 and IRF6, and low IRF3 levels in tumors had significantly poorer OS.^456^ Similarly, a study based on bioinformatics database and proteomics reported that PDAC patients with lower IRF3 expression had shorter OS.^457^ However, another study described that IRF3 promoted circRNA ubiquitin-like with PHD and RING finger domain 1 (circUHRF1) expression, which can regulate ADP ribosylation factor like GTPase 4 C (ARL4C) expression and then induce PDAC progression by sponging miR-1306-5p.^458^ The role of IRF6 in PC is currently unclear, Xie et al. revealed that *IRF6* might be one of the risk genes in pyroptosis-related genes (PRG), and high PRG prognostic index had a lower probability of survival in PC patients based on the TCGA dataset.^459^

A recent study showed that IRF4 played a critical role in shaping the immune cell composition within the tumor microenvironment (TME) of murine PC and deficiency of IRF4 accelerated tumor growth and reduced survival, combined with a dense tumor infiltration with polymorphonuclear myeloid-MDSC (PMN-MDSC) and reduced numbers of CD8^+^ T cells.^460^ Similarly, the repression of IRF5 and IRF7 promotes MYC proto-oncogene, bHLH transcription factor (MYC)/ KRAS proto-oncogene, GTPase (KRAS)-dependent evasion of B cells and NK cells in PDAC, and the de-repression of IRF5 and IRF7 results in increased survival.^461^ Furthermore, Meyer et al. revealed that reduced IRF8 level in circulating pre-DCs was correlated with decreased OS and relapse-free survival (RFS) in PC patients.^462^

In conclusion, IRF1 can suppress mitochondrial respiration and fatty acid synthesis in PC cells and induce PC cell necroptosis. In addition, the inhibition of IRF4, IRF5, IRF7, and IRF8 decreases the survival of PC patients, indicating these IRFs probably act as anti-tumor factors in PC. On the other hand, IRF2 and IRF6 may function as oncogenic proteins due to they are negatively related to PC patients’ survival, and IRF2 can inhibit PC cell apoptosis. Although lower IRF3 is associated with shorter OS for PDAC patients, it upregulates circUHRF1 to induce PDAC progression.

#### Colorectal cancer (CRC)

IRF1 is a protective factor and functions as a tumor suppressor to prevent CRC progress. Hong et al. revealed that IRF1 expression was reduced in CRC and IRF1 level was inversely associated with CRC metastasis.^463^ A study based on bioinformatics methods indicates that CRC patients with higher IRF1 expression presented better RFS time, and the DNA methylation in the IRF1 gene region is indicated to be negatively correlated with IRF1 expression and positively correlated with CRC recurrence.^464^ Moreover, IRF1 can promote Ras association domain-containing protein 5 (RASSF5) expression, and then suppress CRC proliferation and metastasis, possibly through downregulating the RAS-Rac family small GTPase 1(RAC1) pathway.^463^ IRF1 positively regulates PANoptosis to restrict tumorigenesis in CRC.^275^ More recently, Yuan et al. found that IRF1 reduces autophagy related 13 (ATG13) expression to inhibit autophagy and induce apoptosis, which further prevents CRC progression.^465^ Besides, upregulated IRF1 can promote the radiosensitivity of CRC both in vitro and in vivo.^91^

Sun et al. reported that CRC patients with high exosomal IRF2 content had a poorer OS rate compared to patients with lower exosomal IRF2 content.^466^ Exosomal IRF2 can induce vascular endothelial growth factor C (VEGFC) release by macrophages, and inhibition of IRF2 attenuates the lymphatic network remodeling in the sentinel lymph node (SLN) and suppresses the SLN metastasis in CRC.^466^ However, Liao et al. revealed that CRC patients with low IRF2 expression may be non-responders to anti-PD-1, while patients with high IRF2 expressional most presented a complete response, partial response, or stable disease.^467^ Furthermore, IRF2 suppresses the migration and infiltration of MDSCs by repressing the CXCL3/CXCR2 axis, and IRF2 overexpression in microsatellite instability-high (MSI-H) CRC overcomes the resistance of tumors, expressing KRAS to anti-PD-1 therapy.^467^

IRF3 acts as both a tumor promoter and suppressor in different studies. A bioinformatic investigation reported that IRF3 was upregulated in CRC and suggested a shorter survival time in CRC patients.^468^ Besides, another study revealed that drugs like wogonin exhibit anti-tumor activity against CRC by downregulating IRF3 expression.^469^ However, Tian et al. identified IRF3 as a tumor suppressor and a prognosis marker for CRC patients.^470^ IRF3-deficient mice are more susceptible to the development of intestinal tumors and IRF3 negatively regulates the Wnt/β-catenin pathway to prevent tumorigenesis in CRC.^470^ In fact, β-catenin may exert oncogene activity in part through impeding the nuclear translocation of IRF3 and then inhibiting the IRF3-mediated immune signaling pathway in CRC cells.^471,472^ Similar to IRF3, the role of IRF7 in CRC development is also controversial. Chen et al. reported that IRF7 was also upregulated in CRC and suggested a shorter survival time in CRC patients.^468^ On the other hand, evidence showed that tumor-infiltrating pDCs with positive IRF7 expression were associated with prolonged survival of CRC patients,^473^ and 5-aza-2-deoxycytidine exhibited anti-tumor efficacy via activating MDA5/MAVS/IRF7 pathway in CRC-initiating cells,^474^ suggesting that IRF7 may have an anti-tumor effect in CRC.

There are a few researches focused on the role of IRF4, IRF5, IRF6, IRF8, and IRF9 in CRC. Wang et al. revealed that IRF4 was downregulated in colon cancer tissues and that overexpression of RNF2 could increase the ability of proliferation, migration, and invasion in colon cancer cells (SW480 cells) by promoting the ubiquitination and degradation of IRF4.^96^ Moreover, Udden et al. showed that NOD2 plays a vital role in the suppression of inflammation and tumorigenesis in the colon via inducing IRF4 and then downregulating the TLR signaling pathways.^475^ Integrated multi-omics analyses identified that patients who carried concurrent *IRF5* and *NEFH* mutations had worse survival outcomes.^476^ Hu et al. presented that IRF5 probably was a significant mediator of DNA damage signaling and the induction/activation of IRF5 signaling pathway might enhance CRC cell death with chemotherapeutic agents.^477^ In addition, Arnold et al. revealed that GM-CSF induced IRF5 activation in eosinophils and then promoted antitumor immunity in CRC.^478^ Tan et al. found that IRF6 expression was reduced in CRC and liver metastasis, and IRF6 enhanced CRC cell sensitivity to cisplatin to inhibit cell proliferation, migration, and invasion along with aggravating cell apoptosis.^479^ Ibrahim et al. described that human colorectal carcinomas had significantly lower IRF8 expression and higher IRF8 promoter DNA methylation compared to normal colon, and mice with Irf8 deleted in colonic epithelial cells showed higher inflammation-induced tumor incidence.^480^ IRF8 can repress osteopontin expression in colon epithelial cells, and IRF8 expression is silenced during the transformation of colon epithelial cells into colon tumor cells, indicating that tumor cells may inhibit IRF8 to upregulate osteopontin, which functions as an immune checkpoint to restrict CTL activation and promote host tumor immune tolerance and evasion.^481^ On the other hand, IRF9 may promote CRC development by activating IL-6/STAT3 pathway in CRC models.^482^

These evidences illustrate that IRF1, IRF4, IRF5, IRF6, and IRF8 may be candidate tumor-suppressors in CRC. IRF1 enhances RASSF5 expression and CRC cells PANoptosis as well as radiosensitivity to suppress CRC tumorigenesis and progression. IRF5 induces CRC cell DNA damage, and cell death, and enhances antitumor immunity. IRF6 contributes to CRC cell apoptosis and enhances sensitivity to cisplatin chemotherapy. However, the roles of IRF2, IRF3, and IRF7 in CRC are controversial. Although exosomal IRF2 induces VEGFC release and the inhibition of IRF2 suppresses SLN metastasis in CRC, IRF2 also inhibits the CXCL3/CXCR2 axis to prevent CRC, and its overexpression reverses CRC cells’ resistance to anti-PD-1 treatment. Similarly, high expression of IRF3 and IRF7 led to poor survival time in CRC patients. On the other hand, IRF3 suppresses Wnt/β-catenin to inhibit CRC tumorigenesis and IRF7 activation induces anti-tumor activity in CRC cells.

#### Lung cancer

Lung cancer is divided into two broad categories: the common non-small cell lung cancer (NSCLC) and the relatively rare SCLC.^483^ NSCLC can be further divided into lung adenocarcinoma (LUAD), lung squamous cell carcinoma (LUSC), and large cell carcinoma (LCC).^484^ Decreased IRF1 has been reported in NSCLC tissues,^485,486^ and inhibition of the IRF1/IFN-γ pathway by phosphoserine aminotransferase 1 promotes LUAD metastasis.^487^ Frequent genomic aberrations of IRF1 were also found in LUAD metastatic samples by regulator network analysis based on TCGA.^488^ IRF1 acts as a prognostic factor for NSCLC and acts as a potential tumor suppressor by suppressing oncogenic protein Karyopherin alpha 2 (KPNA2) gene expression in LUAD cells.^485^ Zhang et al. revealed that the first-line NSCLC chemotherapy drug cisplatin, could induce IRF1 activation in vitro, which further enhanced mitochondrial depolarization, oxidative stress, apoptotic cell death, and suppressed autophagy in NSCLC A549 cells,^486^ indicating that IRF1 is a possible target for promoting the sensitivity to cisplatin therapy in NSCLC.

The possible function of IRF2 in lung cancer is unclear. A few studies demonstrated that both miR-18a-5p and miR-1290 promote NSCLC proliferation and invasion by suppressing IRF2.^489,490^ Moreover, lncRNA GAS5 and CASC2 can inhibit the progression of NSCLC and repress cisplatin resistance of NSCLC in vitro and in vivo through inhibiting miR-221-3p and miR-18a, thus increasing IRF2 expression in NSCLC cells, respectively.^491,492^ However, another study reported that miR-450 could inhibit lung cancer progress by downregulating IRF2 in vitro and in vivo.^493^ These researches indirectly indicate that IRF2 acts as both a protective and risk factor in NSCLC.

Yi et al. revealed that overexpressed IRF3 induced the apoptosis of NSCLC cells and phosphorylated IRF3 (p-IRF3) was decreased in NSCLC samples.^494^ Gong et al. presented that inhibiting mutant epidermal growth factor receptor (EGFR) activated the RIG‑I/TBK1/IRF3 pathway and then resulted in a tumor-suppressive role in NSCLC with EGFR-activating mutations.^495^ Zhang et al. found that Niraparib and radiation synergistically inhibited tumor immunity in EGFR-mutated NSCLC both in vitro and in vivo, accompanied by increased CD8^+^ T lymphocytes and the STING/TBK1/IRF3 pathway activation.^496^ Similarly, other studies described that inhibition of WEE1 G2 checkpoint kinase (WEE1) or use of carboplatin or other DDR inhibitors could activate the STING/TBK1/IRF3 pathway, promoting the antitumor immune response to PD-L1 blockade in lung cancer models.^497–499^ In addition, Long et al. illustrated that G protein-coupled receptor 162 could act as a novel radiotherapy sensitizer by interacting with STING, which then promoted DDR and activated IRF3 to inhibit the occurrence and development of tumors in lung cell lines.^500^

Some studies reported that IRF4 in peripheral mononuclear cells was a protective prognostic factor in NSCLC patients and LUAD patients with high IRF4 expression correlated with significantly longer OS.^501,502^ Another study pointed out that IRF4 in tumor tissues was associated with poorer survival of NSCLC patients.^503^ In fact, IRF4 facilitates macrophages M2 polarization, and the E74 like ETS transcription factor 4 (ELF4)/IRF4 pathway activation by exosomal circZNF451 in LUAD patients reshapes the immunosuppressive TME and impedes the effect of PD-1 blockade treatment.^504^ IRF4 downregulation by liver X receptors (LXR) contributes to the anti-tumoral effects of LXR activation in lung carcinoma models.^505^ Besides, thigh-dimensional single-cell profiling of T cells from chemotherapy-naive patients with NSCLC revealed that IRF4^+^ CD4^+^ effector Tregs suppress antitumor immunity through expressing molecules related to enhanced immunosuppression.^506^

Feng et al. found reduced IRF5 expression in human NSCLC tissues,^507^ while Guo et al. revealed that IRF5 level was significantly higher in the peripheral blood of NSCLC patients.^508^ Yamashina et al. identified IRF5 as a specific factor for cancer stem-like cells from chemoresistant tumors and it was important for M-CSF and tumorigenic myeloid cellsgeneration. IRF5/M-CSF pathway activation in tumor cells was correlated with the number of tumor-associated CSF1 receptor^+^ M2 macrophages in NSCLC patients.^509^ IRF6 level was also reported to be upregulated in both LUAD and LUSC tissues, and inhibition of IRF6 revealed the antitumor effects in lung cancer cells.^510^ Huang et al. demonstrated that higher IRF7 levels in LUAD tumors were associated with higher microvessel density in LUAD tissues, potentially responsible for the hemorrhage outcomes following bevacizumab treatment.^511^ Besides, IRF7 can enhance constitutive PD-L1 expression though directly promoting transcription of PD-L1 and tumor cell immune evasion, and methylated IRF7 is negatively correlated to PD-L1 expression in NSCLC.^512^

Suzuki et al. revealed that the IRF8 methylation level was significantly higher in NSCLC specimens compared to non-malignant lung tissues, and that IRF8 methylation may act as a prognostic marker for NSCLC recurrence.^513^ In addition, Liang et al. showed that IRF8 expression was frequently diminished in lung tumoral tissues, and IRF8 induced lung cancer cell senescence through inhibiting AKT signaling and promoting P27 protein accumulation.^514^ However, survival analyses based on the TCGA database reported that LUAD patients with higher expression levels of IRF8 were associated with poor survival.^515^ IRF9 has been reported to be overexpressed in lung cancer and is associated with poor survival, promoting lung cancer progression via the upregulated expression of oncogene versican.^516^

In conclusion, IRF1 and IRF3 present inhibitory functions in lung cancer. IRF1 activation induces lung cancer cell death and suppresses KPNA2 expression to inhibit LUAD growth. IRF1/IFN-γ inhibition facilitates LUAD metastasis. IRF3 contributes to NSCLC apoptosis and the activation of RIG-I/STING/TBK1/IRF3 pathway inhibits NSCLC growth and increases antitumor immunity. Conversely, IRF5, IRF6, IRF7, and IRF9 may induce lung cancer progression. IRF5/CSF activation facilitates tumor-associated M2 expression, and IRF7 induces PD-L1 production. IRF9 increases versican expression,which is associated with poor survival in lung cancer patients. Decreased IRF6 showed an antitumor effect. The detailed functions of IRF2, IRF4, and IRF8 in lung cancer are complex and controversial. On the one hand, IRF2 inhibition by miR-18a-5p and miR-129 induces NSCLC proliferation and invasion. However, IRF2 inhibition by miR-450 suppresses lung cancer cells’ progress. IRF4 overexpression causes poor survival in NSCLC patients, and ELF4/IRF4 axis activation causes M2 polarization to promote LUAD. Inhibition of IRF4 facilitates the LXR anti-tumor effect; while other studies have shown that IRF4 expression contributes to long OS in lung cancer patients. IRF8 has been reported to suppress ATK and induce P27 expression to cause lung cancer cell senescence, and IRF8 methylation leads to NSCLC recurrence. Another report showed that IRF8 upregulation was associated with poor survival of LUAD patients.

#### Ovarian cancer (OC)

Transcriptome analysis identified IRF1 was associated with platinum sensitivity, and its overexpression was correlated with increased OS and progression-free survival in high-grade serous OC (HGSOC).^517^ However, another study revealed that IFN-γ induced IRF1 expression, which was followed by increased PD-L1 expression in OC cells, and IRF1 inhibition attenuated the IFN-γ-induced gene and PD-L1 protein levels. Moreover, IFN-γ-induced PD-L1 expression is modulated by the JAK1/STAT1/IRF1 pathway in OC.^518^ Additionally, activation of the TLR4/IL-6/IRF1 signaling causes androgen receptor (AR) upregulation and taxol resistance genes transactivation in OC.^519^ Furthermore, the chemotherapeutic drug cisplatin upregulates IRF1 expression, which in turn limits the cell response to cisplatin in OC cells,^520^ indicating that IRF1 is a potential target in OC chemoresistance and progression.

Zhang et al. showed that deubiquitinase USP35 was significantly elevated in cisplatin-resistant OC cells, partially inhibiting the STING/TBK1/IRF3 pathway and then resisting the antitumor effect of IFN-I.^521^ On the other hand, POL I inhibitor CX-5461 activates cGAS/STING/TBK1/IRF3 pathway, increasing immunotherapy efficacy in OC.^522^ The immunostaining analysis showed that IRF4 was expressed in most HGSOC patients;^523^ however, the possible function of IRF4 on OC remains unclear. Zhang et al. illustrated IRF5 overexpression in combination with its activating kinase IKKβ reprogramed TAMs to a phenotype that induced anti-tumor immunity and promote tumor regression in OC model.^524^ In addition, IRF9 was reported to be the critical upstream regulator to mediate growth-inhibitory effects of IFN-α on OC cells, and the anti-tumoral effect of chemerin on OC cells in vitro was regulated by the activation of IFN-α response genes via IRF9.^525,526^ Therefore, IRF3, IRF5, and IRF9 may be protective and function as anti-tumor factors in OC.

#### Prostate cancer (PCa)

Adenocarcinoma is the major type of PCa.^527^ Bioinformatics analysis revealed that IRF1 was a tumor suppressor within this core transcriptional regulatory network in modulating PCa cell proliferation.^528^ IRF1 was also associated with necroptosis in the prostate adenocarcinoma cell line PC-3 cells,^529^ and oncogenic factor the Deltex-3-like E3 ubiquitin ligase (DTX3L) can induce proliferation and survival of metastatic PCa cells via the inhibition of IRF1.^530^ Wu et al. identified that IRF8 expression was increased in primary PCa but reduced in castration-resistant prostate cancer compared with normal prostate tissue.^531^ IRF8 promoted the degradation of the AR and decreased IRF8 facilitated AR-induced PCa progression and enzalutamide resistance, indicating that IRF8 may act as a tumor suppressor in PCa pathogenesis and represent a potential therapeutic option to defeat enzalutamide resistance.^531^

Although the detailed function of IRF2, IRF3, and IRF7 in PCa remain unclear, studies demonstrated that miR-221 could inhibit cell growth and invasiveness, and induce cell apoptosis, in part by suppressing IRF2 and SOCS3 in PCa cells, and anti-cancer drug nobiletin inhibits PCa cell growth through suppressing TLR4/TRIF/IRF3 and TLR9/IRF7 pathways.^532,533^ On the contrary, Zhao et al. illustrated that IRF7 overexpression in PCa cells had a marked effect on inhibiting bone metastases in xenograft nude mice.^534^

#### Renal cancer

IL-4 was reported to increase IRF1 expression, which then inhibited the proliferation of human renal cell carcinoma (RCC) cell lines.^535^ Tomita et al. demonstrated that IFN-γ induced STAT1/IRF1 activation, followed by caspase-7-mediated apoptosis in RCCs cell line ACHN cells.^536^ However, with IFN-γ stimulation, activation of JAK2/STAT1/IRF1 signaling also induced PD-L1 expression in both WT- and Mut-VHL clear cell RCC (ccRCC) cells.^537^ IRF3 was overexpressed in ccRCC and significantly associated with worse clinical outcomes and adverse OS in ccRCC patients based on databases.^538^ The multi-omics analysis on ccRCC samples extracted from Gene set enrichment (GSE) and TCGA data sets identified IRF4 as a protective factor in ccRCC.^539^ Moreover, Wang et al. illustrated that Zinc finger protein 692 induced tumorigenesis in ccRCC via the transcriptional repression of IRF4 and other genes.^540^ IRF6 expression was reduced in ccRCC tissues and cell lines, and downregulated IRF6 was associated with worse clinicopathological features and poorer prognosis.^541^ IRF6 inhibited ccRCC cell proliferation, invasion, and migration at both the cellular and animal levels which may be through the inhibition of kinesin family member 20 A (KIF20A).^262^ Studies illustrated that IRF7 was one of upregulated differentially expressed genes in ccRCC samples and patients with high IRF7 expression presented a relatively worse survival rate.^542,543^ CircEGLN3 sponged miR-1299 to enhance the IRF7 level, which is responsible for RCC cell proliferation and aggressiveness in vitro.^543^ It was reported that ccRCC patients with high IRF8 expression within metastatic sites had longer OS than those with low IRF8 expression.^544^ IRF8 was identified to inhibit RCC cell colony formation and migration through promoting cell cycle G2/M arrest and apoptosis, upregulating tumor suppressor genes like caspase-1, p21, and PTEN expression, and inhibiting oncogenes YAP1 and survivin expression.^545^ However, Nixon et al. revealed that TAMs expressed IRF8 to promote T cell exhaustion in RCC and other cancers, and ccRCC patients with abundant CD8^+^ T cell infiltration might have worse survival if they expressed higher IRF8-TAM gene signature.^546^

In conclusion, IRF1 and IRF8 have dual effects on renal cancers. IRF1 can inhibit RCC proliferation, and STAT1/IRF1 activates caspase-7-mediated apoptosis in RCC cells; meanwhile, the JAK2/ STAT1/IRF1 pathway also induces PD-L1 expression in ccRCC cells. Similarly, IRF8 induces G2/M arrest and cell apoptosis, upregulates tumor suppressors p21, and PTEN and inhibits oncogenic YAP1 and surviving, thus suppressing RCC cell colony formation and migration; but IRF8^+^TAMs promote T cell exhaustion, which is associated with worse survival in ccRCC patients. IRF3 and IRF7 may be oncogenic factors due to they are associated with worse outcomes and survival in ccRCC patients, and IRF7 facilitates RCC cell proliferation and aggressiveness. On the other hand, IRF4 and IRF6 are tumor suppressors. ZNF692 represses IRF4 to induce ccRCC tumorigenesis and IRF6 inhibits cRCC cell proliferation, invasion, and migration.

#### Leukemia

The most common leukemias are acute myeloid leukemia (AML), acute lymphocytic leukemia/lymphoma (ALL), chronic lymphocytic leukemia/small lymphocytic lymphoma (CLL/SLL), and CML. There are also some relatively rare leukemias, such as chronic myelomonocytic leukemia (CMML) and adult T-cell leukemia/lymphoma (ATL). IRF1 plays a vital role in myeloid differentiation by acting as a tumor suppressor gene in leukemia, and mutations in IRF1 are likely to influence IRF1 and its DNA binding affinity, leading to the inhibition of cancer suppression.^547^ Semmes et al. identified *IRF1* SNP as one risk locus in ALL.^548^. Massimino et al. showed that IRF5 exerted antiproliferative effects, inhibited B-cell receptor (BCR)-ABL signaling, and increased the cytotoxicity of imatinib mesylate in both immortalized and primary CML cells.^549^ Mathew et al. presented that with sorafenib treatment, active IRF7 (p-IRF7/t-IRF7) levels were increased in both mouse and human leukemia cells, which then facilitated IL-15 production. This, in turn, promoted immune memory against tumor cells, inducing an immune-mediated cure of AML^FLT3-ITD^-relapse.^550^ Moreover, IRF7 overexpression decreased AML cell proliferation and leukemia stem cell levels, while IRF7 knockout accelerated AML progression and induced vascular cell adhesion molecule 1 (VCAM1)- very late antigen 4 (VLA-4) mediated intracerebral invasion in AML mouse models.^551^ In addition, IRF7/stress-activated protein kinase (SAPK)/JNK pathway activation resulted in more M1 characteristics and contributed to prolonged survival in leukemia mice.^552^ Tian et al. revealed that both IRF9 mRNA and protein levels were decreased in human AML samples.^553^ IRF9 is a negative regulator for AML, which binds with the SIRT1 promoter, represses SIRT1 expression, and promotes p53 expression to inhibit AML cells proliferation, colony formation, and survival.^553^ These evidences suggest that IRF1, IRF5, IRF7, and IRF9 are tumor suppressors in leukemia.

However, studies revealed that IRF2 and IRF3 act as oncoproteins in leukemia. IRF2 promotes inositol polyphosphate-4-phosphatase, type-II (INPP4B) expression, which then facilitates AML cell growth and colony formation, and IRF2/INPP4B pathway activation represses apoptosis through induction of autophagy combined with the inhibition of Th1 cell differentiation and promotion of Th2 cell differentiation in AML cells.^554–556^ In addition, the inhibition of IRF2 and the IRF2/INPP4B axis by miR-222-3p exposed to human bone marrow mesenchymal stem cells (hBM-MSCs-Exo) suppressed proliferation and promoted apoptosis of AML cells; while overexpressed either IRF2 or INPP4B resisted the proliferation-inhibitory and pro-apoptotic effects mediated by BM-MSCs-Exo.^557^ Tian et al. showed that both IRF3 mRNA and protein levels were significantly increased in AML samples than in those from healthy controls.^558^ The inhibition of IRF3 restricted AML cell proliferation and colony formation, and promoted AML cell apoptosis, whereas overexpression of IRF3 promoted AML cell survival, which was positively correlated with the oncogenic presence of miR-155 in AML.^558^

A genome-wide association study (GWAS) study revealed that IRF4 was a major susceptibility gene for CLL and AML.^559,560^ Higher IRF4 levels attenuated BCR signaling by negatively regulating the expression of the spleen tyrosine kinase (SYK) and IKAROS in CLL cells.^561^ Additionally, murine CLL with low B cell-specific IRF4 expression showed a poorer prognosis due to enhanced tumor immune evasion.^562^ Furthermore, IRF4 expression in CD3^+^ T cells from CML patients was significantly reduced.^563^ CML patients who achieved an early molecular response had higher IRF4 values at both diagnoses and after 3 months of therapy compared to those who did not achieve early molecular response.^564^ In addition, PU.1 can collaborate with IRF4 and IRF8 to repress pre-B-cell leukemia.^565^ It was found that repression of IRF4 by miR-125b induced both myeloid and B-cell leukemia in vitro.^566^ However, Sun et al. demonstrated that miR-155-5p inhibited IRF4 protein degradation, thereby promoting cyclin dependent kinase 6 (CDK6) expression and facilitating childhood ALL development.^97^ IRF4 is also an oncogenic transcription factor in adult ATL. *IRF4* gene was expressed higher in ATL cells than in normal T cells, and overexpression of IRF4 induced the upregulation of oncogenes such as MYC and baculoviral IAP repeat-containing protein 3 (BIRC3).^567^ Moreover, the CDK9 inhibitor alvocidib could downregulate super-enhancers-mediated IRF4, thereby inhibiting ATL cell proliferation.^568^

Watanabe et al. demonstrated that IRF8 expression was downregulated in CML patients, and Irf8^-/-^ mice developed a CML-like disease.^569^ IRF8 partially suppressed CML development in vivo through a Fas-dependent apoptosis mechanism.^570^ IRF8 acts as a roadblock for β-catenin-driven leukemia. The deletion of IRF8, combined with constitutive β-catenin activation, resulted in the progression of CML into a fatal blast crisis and imatinib resistance.^571^ Additionally, the loss of IRF8 facilitates the initiation of acute promyelocytic leukemia (APL).^572^ Furthermore, IRF8 has been shown to suppress T-cell ALL proliferation and invasion by suppressing the PI3K/AKT pathway.^573^ However, some studies have found that high IRF8 expression was associated with poorer prognoses in AML patients, and IRF8 loss inhibited AML cell growth.^574,575^ Pingul et al. uncovered that the transcriptional circuit of myocyte enhancer factor 2D (MEF2D)/IRF8 was required for AML maintenance.^576,577^

The single-cell RNA sequencing study demonstrated that CMML-2 stem cells from CMML patients were characterized by highly expressed regulome associated with IRF1, IRF7, and IRF8, factors that are highly expressed in monocytic lineage differentiation.^578^ However, the detailed functions of these IRF members in CMML require further exploration.

#### Multiple myeloma (MM)

The roles of IRF1 and IRF3 in MM are complex. Wang et al. demonstrated that all-trans retinoic acid activated the retinoic acid receptor γ and IFN-β response pathway, which induced IRF1 overexpression to initiate 2’-5’-oligoadenylate synthetase 1 (OAS1) transcription. This caused cellular RNA degradation and cell death, enhancing MM sensitivity to carfilzomib (Cfz)-induced cytotoxicity and resensitizing Cfz-resistant MM cells to Cfz in vitro.^579^ Additionally, drug MEDI2228 activated the cGAS/STING/TBK1/IRF3 and STAT1/IRF1-induced IFN-I pathway to fight MM cell immune evasion.^580^ Moreover, macrophage inflammatory protein 1α (MIP-1α) induced osteoclast formation through activating the MEK/ERK/c-Fos pathway and suppressing the p38 map kinase (p38MAPK)/IRF3 pathway and IFN-β expression in MM.^581^ However, Liu et al. found that myeloma cell-secreted 2-deoxy-D-ribose induced the STAT1/IRF1 pathway activation and then upregulated CIITA expression in osteocytes to facilitate myeloma-induced bone lesions.^582^ In myeloma plasma cells, the interaction of ZBP1 with TBK1 and IRF3 causes IRF3 phosphorylation, which allows IRF3 to directly bind to and activate cell cycle genes, in part through cooperation with IRF4, promoting myeloma cell proliferation in MM.^257^

IRF4 is highly expressed in MM cells and is strictly required for MM cell survival, downregulating pro-apoptotic BCL2-modifying factor (BMF) and BCL2L11.^583^ Moreover, the oncogenic MAF bZIP transcription factor (MAF) activated enhancers and super-enhancers in B cells and plasma cells and cooperated with the plasma cell IRF4 to endow myeloma plasma cells with migratory and proliferative transcriptional potential.^584^ Overexpression of IRF4 and the oncogene c-Myc is a major mechanism of lenalidomide resistance in MM. Inhibition of SUMOylation enzymes suppressed IRF4 gene transcription and reduced IRF4 protein level by enhancing IRF4 degradation to enhance lenalidomide sensitivity.^585^ In addition, lenalidomide selectively degraded IKZF1, leading to IRF4 repression and IRF5 increase, which then promotes macrophage polarization from an anti-inflammatory M2-like phenotype toward a tumoricidal M1 phenotype.^586^ IRF4 down-regulation inhibited tumor formation and myeloma dissemination, eradicated myeloma progenitors, and improved survival and sensitivity to myeloma drugs.^587,588^ The bioinformatics analysis revealed that IRF7 was an important immune-related gene in MM patients, and it could promote tumor progression in vitro.^589^ An *IRF8* mutation was reported in MM patients by the GWAS.^590^ In Myeloma cells, heterogeneous nuclear ribonucleoprotein A2/B1 (hnRNPA2B1) upregulated miR-92a-2-5p and miR-373-3p expression, activating osteoclastogenesis and suppressing osteoblastogenesis through inhibiting IRF8 or RUNX family transcription factor 2 (RUNX2) to drive MM osteolytic bone disease.^591^ These studies reveal that IRF4 and IRF7 are oncogenic, while IRF5 and IRF8 may act as tumoricidal factors in MM.

#### Myelodysplastic syndrome (MDS)

Although IRF1 is regarded as a tumor suppressor in pre-leukemia myelodysplasia,^547^ it has been reported that myelodysplasia patients without IRF1 expression had a decreased incidence of autoimmune manifestations, indicating that IRF1 may increase the probability of autoimmune phenomena in MDS, companied by a decline in quality of life.^592,593^ Compared to controls, MDS patients displayed upregulated expression of IRF2, IRF3, and IRF7.^594^ TLR3 hyperactivation can induce IRF3, IRF7, and NF-κB translocating to the nucleus, which then activates IFN-α/β transcription to promote the IFN response and result in aggressive MDS.^595^ The GWAS and bioinformatic analysis showed that IRF4 was a major susceptibility gene for MDS, and patients with IRF4 alterations presented worse OS than those without alterations.^560,596^

#### Head and neck SCC (HNSCC)

Wang et al. demonstrated that the inhibition of CTLA4 activated CD8^+^ T cells and increased IFN-γ and TNF-α levels, which in turn induced the STAT1/IRF1 axis activation to trigger tumor cell pyroptosis with tumor cell death in HNSCC cell lines.^597^ IRF2-induced claudin-7 upregulation to suppress oral SCC cell lines proliferation, invasion, and migration.^598^ The m6A demethylase alkB homolog 5, RNA demethylase (ALKBH5) overexpression inhibited RIG-I protein, resulting in the downregulation of IFN-α secretion mediated by the IKKε/TBK1/IRF3 axis. This reduction leads to reduced immune-killing cell infiltration and facilitates HNSCC progression and immune evasion.^599^ Yan et al. displayed that Irf6 loss triggered rapid HNSCC development in mouse models, while Notch/Ripk4/Irf6 axis activation suppressed tumor growth in vitro.^600^ These findings illustrate that IRF1, IRF2, IRF3, and IRF6 play an inhibitory effect on HNSCC.

#### Nasopharyngeal carcinoma (NPC)

IRF1 has been identified as one of the motifs enriched in the NPC unique cluster;^601^ however, the deeper function of IRF1 in NPC remains unclear. The IRF2 motif is also enriched in the NPC unique cluster,^601^ and IRF2 promotes centromere protein N (CENP-N) expression in NPC cells, further facilitates NPC cell proliferation, cell cycling, and apoptosis resistance, alongside increased aerobic glycolysis.^602^ Ge et al. demonstrated that circBART2.2 promoted PD-L1 expression via activating IRF3 and NF-κB in NPC, thereby inducing tumor immune escape.^603^ These studies suggest that IRF2 and IRF3 likely act as tumor promoters in NPC. On the other hand, Xu et al. demonstrated that IRF6 was reduced in highly metastatic NPC cells, cancer stem-like NPC cells, and NPC animal models.^261^ IRF6 directly restricts ATP-binding cassette sub-family G member 2 (ABCG2) expression in NPC cell lines and NPC tissues, resulting in the inhibition of NPC cancer cell proliferation, colony formation, and self-renewal.^261^

#### Cholangiocarcinoma (CCA)

IRF1 was found to be low expressed in CCA, and IRF1 acted as a tumor suppressor in regulation of CCA cell proliferation, cell cycle, migration, and invasion.^604^ Moreover, miR-383 could enhance proliferation, migration, and invasion of CCA cells via negatively regulating IRF1.^605^ Instead, IRF4 could upregulate lncRNA SOX2-OT, which further upregulated SOX2 and activated PI3K/AKT pathway to promote CCA cell proliferation and metastasis.^606^

#### Breast cancer (BC)

The potential roles of IRF1 and IRF9 in BC are controversial. IRF1 was found to be overexpressed in patients with highly aggressive BC subtypes, facilitating EMT, migration, dissemination, and metastasis formation.^607^ Moreover, Wu et al. showed that ubiquitin ligase E3 component N-recognition protein 5 (UBR5) enhanced PD-L1 transactivation by upregulating PKR, STAT1 and IRF1 to induce tumorigenesis in triple-negative breast cancer (TNBC).^608^ On the other hand, IRF1 can upregulate autophagy and apoptosis, and inhibit NF-κB activity to inhibit BC cell growth.^609,610^ IKKε showed its oncogenic potential by accelerating IRF1 degradation and inhibiting IRF1 transcriptional activity in a BC cell line.^53^ Luker et al. reported that IRF9 overexpression caused resistance to antimicrotubule agents in BC cells.^611^ However, Brockwell et al. displayed that IRF9 was a marker of reduced risk of distant relapse, and IRF9 loss was a poor prognostic biomarker for pre-chemotherapy in TNBC.^612^

Studies have revealed that IRF2, IRF3, IRF4, IRF5, IRF6, and IRF8 are protective factors in BC progression. Kriegsman et al. revealed that IRF2 negatively regulated PD-L1 expression to inhibit BC immune evasion.^390^ IRF3 was shown to facilitate retinoic acid (RA)/polyinosinic-polycytidylic acid (poly(I: C))-induced apoptosis in BC cells, driving the death of BC cells.^613^ Activated cGAS-STING-IRF3 pathway by cGAMP reversed the EMT and PI3K/AKT pathways and prevented TNBC metastasis.^614^ STING/TBK1/IRF3 pathway activation to recruit CD8^+^ T cells is a critical determinant of the therapeutic efficacy of PARP inhibition in TNBC.^615^ IRF4 was regarded as a tumor suppressor and higher IRF4 expression was associated with improved outcome in node-negative BC.^616,617^ Overexpression of IRF5 in BC cells inhibited in vitro and in vivo cell growth, and migration, and sensitized them to DNA damage. Additionally, IRF5 downregulation was correlated with increased invasiveness in human ductal carcinoma.^618,619^ IRF6 was downregulated in highly invasive BC cell lines, but increased in poorly aggressive ones, and drugs like bortezomib and lapatinib upregulated IRF6 expression to inhibit BC growth.^620–622^ BC-produced GSF decreased IRF8 in cDC progenitors, which was followed by reduced cDC1 and subsequently impeded immune surveillance in BC, impairing anti-tumor CD8^+^T-cell responses and causing poor outcomes in BC patients.^462^ Additionally, IRF8 deficiency negatively impacted BC therapeutic efficacy.^623^

#### Cervical cancer (CC)

The most common histological subtype of cervical cancer (CC) is SCC, followed by adenocarcinoma.^624^ The human papillomavirus 16 (HPV16) E6 oncoprotein facilitates CC growth and angiogenesis, while IRF1 inhibits tumourigenesis and the angiogenic activity of HPV16 E6 in CC.^625^ In addition, patients with overexpressed IRF1 in pretreatment CC biopsy cells had a significantly better response to neoadjuvant radio/chemotherapy, and STAT3/IRF1 pathway activation sensitizes CC cells to chemotherapeutic drugs.^626^ IFN-γ/IRF1 signaling also upregulates P27, which inhibits the expression and telomerase activity of human telomerase reverse transcriptase (hTERT), thereby resulting in the inhibition of CC.^627^ IRF4 low expression is associated with a significantly poorer OS in CC patients.^628^ Similarly, it has been reported that IRF6 expression was significantly downregulated in CC specimens and cell lines, and miR-587 could repress IRF6 protein expression to abrogate the antineoplastic activity of IRF6 in CC cells and promote HeLa tumor growth.^629^ These results indicate that the loss of IRF1, IRF4, and IRF6 expression facilitates CC development, and IRF1 pathway activation may be beneficial for enhancing the efficacy of CC chemotherapeutic drugs.

#### Endometrial cancer (EC)

Although Kuroboshi et al. reported that IRF1 expression was decreased in human endometrioid adenocarcinoma compared with normal endometrium and postmenopausal endometrium,^630^ Gao et al. revealed that enhanced IRF1 protein stability may upregulate PD-L1 in ECs and promote tumor immune escape.^631^ Zeng et al. presented that IL-6 enhanced reactive oxygen species (ROS) generation and induced mtDNA leakage in EC cells, which further activated the cGAS/STING/TBK1/IRF3 pathway, subsequently PD-L1 production, leading to tumor immune escape.^632^ The adenosine deaminase family acting on RNA1 (ADAR1) knockdown induced the expression of IRF7 and other proteins, which in turn resulted in apoptosis in EC cells.^633^ Therefore, IRF1 and IRF3 probably promote EC cell’s immune escape via PD-L1 production, while IRF7 may inhibit EC growth via inducing apoptosis, which is needed for further investigation.

#### Melanoma

NF-κB/IRF1 axis activation, in association with cDC1s, was linked to better clinical outcomes to improve cancer immunotherapy in melanoma patients.^634^ A bioinformatic analysis revealed that IRF1 expression might act as a biomarker indicating CD8^+^ T cell infiltration in melanoma.^635^ Compared to healthy controls, patients with metastatic melanoma had a significantly lower level of IRF1 signaling molecules in their peripheral blood lymphocytes.^636^ On the other hand, Yokoyama et al. revealed that SOX10 repressed IRF1 transcription via directly inducing IRF4, which then impaired PD-L1 expression in melanoma cells.^637^ Under ultraviolet radiation treatment, IRF3 formed a transcriptional complex with NF-κB/p65 to promote PD-L1 transcription and reduced CD8^+^ T-cell-mediated cytotoxicity, thereby facilitating immune evasion of oncogenic melanocytes and melanoma development.^638^ However, Type 2 transglutaminase negatively regulated IRF3 activation, resulting in reduced IFN-β expression and inducing immune evasion of melanoma cells.^639^ Moreover, Musick et al. found that IRF3 overexpression reduced tumor growth in a melanoma mouse model.^640^ Humblin et al. identified that IRF8 was essential for IL-9-producing Th9 cells anti-tumor effects in mouse melanoma models.^641^ Wang et al. illustrated that the IRF9/STAT2 signaling activation induced adaptive resistance to BRAF inhibitor therapy in melanoma by inhibiting gasdermin E (GSDME)-dependent pyroptosis in melanoma cells and in a xenograft tumor model.^642^ Moreover, the loss of stromal antigen 2 activated IRF9, which in turn enhanced IFN-I signaling and PD-L1 expression in melanoma.^643^

#### Osteosarcoma

Ectopic expression of IRF1 induced caspase-7 and Bcl-2 downregulation to activate apoptosis in cell lines of Ewing’s sarcoma.^644^ IRF1 activated miR-134 to inhibit osteosarcoma cell proliferation, invasion, and migration both in vitro and in vivo.^645^ Moreover, IRF1 positively regulated growth arrest specific 5 (GAS5) expression, causing upregulation of downstream tumor suppressors in osteosarcoma cell lines.^646^ Suppression of IRF1 expression by miR-4295 overexpression promoted osteosarcoma cell proliferation, migration, and invasion.^647^ Similarly, it was reported that the inhibition of IRF2 by miR-18a-5p promotes osteosarcoma cell invasion and migration.^648^ STING/IRF3/ IFN-β pathway activation by a sodium-glucose cotransporter 2 (SGLT2) inhibitor significantly inhibited osteosarcoma growth in vivo.^649^ Higher IRF7 expression tended to a better prognosis in osteosarcoma patients, and IRF7 inhibited PKM2 expression to interfere with the Warburg effect in osteosarcoma cells.^650,651^

#### Gliomas

IRF1 enhanced *LGALS-9* transcription to encode galectin 9 (Gal-9) protein and then drive macrophage M2 polarization. In turn, these macrophages secreted vascular endothelial growth factor A (VEGFA) to promote angiogenesis and glioma growth.^652^ Overexpression of IRF2 protected glioma cells from ferroptosis and enhanced their invasive and migratory abilities in vitro.^653^ On the other hand, IRF3 was reported to inhibit glioma proliferation, migration, and invasion.^654–656^ Yang et al. presented that a therapeutic nanosystem (SPP-ARV-825), targeting the bromodomain and extraterminal-containing protein family 4 (BRD4)-degrading proteolytic chimera, could inhibit IRF4 promoter transcription and STAT6, STAT3, and AKT phosphorylation. This inhibition attenuated cell proliferation, induced cell apoptosis, and suppressed M2 macrophage polarization, leading to an antitumor effect in the glioma xenograft model.^657^ Li et al. showed that the knockdown of Gal-9 activated the TLR7/IRF5 pathway, which facilitated macrophages M1 polarization and enhanced CD8^+^ T cells anti-tumor effect in glioblastoma.^658^ IRF6 inhibited PKM2 and GLUT1 transcription to impair glycolysis and cell proliferation, and induce apoptosis in glioma cells. Furthermore, IRF6 overexpression reduced glioma xenograft tumor growth and prolonged nude mice survival.^263^ In addition, activation of the TLR7/MyD88/IRF5/IRF7 pathway induced IFN-β secretion, which then stimulated NK cells to eradicate glioma cells.^659^

### IRFs and inflammatory and autoimmune diseases

#### Inflammatory bowel disease (IBD)

Tang et al. found that IRF1 was upregulated in human IBD and dextran sulfate sodium (DSS)-induced mice colitis.^660^ They also identified that TNF-α-mediated IRF1 activation suppressed osteopontin expression to inhibit p-AKT, p-P38, and p-ERK activities, and aggravate apoptosis and intestinal epithelial cell injury.^660^ IRF1 deficiency could restrict TNF-α-induced intestinal epithelial cells shedding to maintain intestinal barrier integrity.^661^ Moreover, downregulation of IRF1 expression by miR-24-3p was found to promote M2 polarization and subsequently reduced hyperinflammation-induced damage in the murine colon.^662^ Wang et al. showed that increased activation of IRF3 and IRF7 facilitated inflammatory chemokines expression and promoted excessive intestinal inflammation in LPS-responsive beige-like anchor-deficient mice.^663^ However, Chiriac et al. reported that both *Irf3*^-/-^ and *Irf3*^-/-^*Irf7*^-/-^ mice developed more severe colitis after DSS administration compared to control mice.^664^ Buchele et al. illustrated that IRF4 drove intestinal inflammation via both T cell-intrinsic and T cell-extrinsic mechanisms,^665^ while other studies reported that increased IRF4 expression and suppressed IRF5 phosphorylation ameliorated colitis through inducing M2 macrophage polarization.^666,667^ Yan et al. demonstrated that IRF5 in CD4^+^ T cells accelerated experimental colitis with increased chemokine migration, Th1/Th17 cytokines, and decreased Th2-associated anti-inflammatory cytokines in vivo.^668^ IRF5-NF-κB p65 complex formation disruption or impairment of endogenous IRF5 activation could prevent intestinal inflammation in DSS-mediated colitis.^669,670^ Moreover, inhibition of IRF5 by thalidomide or miR-144/451 could block M1 macrophage polarization or DC activation, which further attenuated colitis in DSS-induced models.^671,672^ Zhang et al. found that the adoptive transfer of CD4^+^T cells with IRF8 deficiency into Rag1^-/-^recipients enhanced colitis development, which was associated with increased gut Tfh-related gene expression, and IRF8 represses Tfh differentiation by suppressing IRF4 transcription and transactivation to prevent IBD development.^673^ However, Veiga et al. reported that IRF8 silencing in leukocytes presented promising anti-inflammatory properties in a murine colitis model.^674^

#### Asthma

Polymorphisms of IRF1 were reported to increase childhood allergic asthma risk, which was associated with increased pro-inflammatory gene regulation.^675^ IRF2 was identified as one of the key candidate genes to regulate genetic susceptibility to asthma in the Indian population.^676^ A study found that intracellular STING/TBK1/IRF3/7 signaling pathway activation by cGAMP exacerbated allergic inflammation and asthma.^677^ Moreover, IRF3 was shown to be essential for house dust mite (HDM)-induced airway allergy, and in *Irf3*^-/-^ mice, HDM-mediated airway allergy were strongly attenuated when compared with those in wild-type mice.^678^ He et al. demonstrated that IRF7 was overexpressed in ILC2s from asthma patients compared to those from healthy controls. Furthermore, IRF7 deficiency impaired the expansion and function of lung ILC2s in multiple models of allergic asthma, leading to the remission of allergic airway inflammation.^679^ Studies uncovered that the activation and upregulation of IRF4 promoted Th2 cell response, M2 macrophage activation, and Th9 cell development, exacerbating allergen-induced lung allergic inflammation. Additionally, the inhibition of IRF4-IL-33 axis activation or IRF4 expression could attenuate allergic inflammation in asthma mouse models.^680–685^ Orissa et al. reported that IRF5 was markedly overexpressed in bronchoalveolar lavage cells of severe asthmatics compared to milder asthmatics or controls. Additionally, IRF5 drove a Th1 cell response and airway hyperreactivity in severe asthma mice.^686^

#### Psoriasis and atopic dermatitis

Based on the in silico analysis, IRF1 was reported to recognize psoriasis response elements and was a psoriasis-activated transcription factor.^687^ Kuai et al. revealed that IRF1 was highly expressed in human psoriasis specimens and that IRF1 inhibition alleviated psoriasis-like inflammation in vitro.^688^ Moreover, TNF-α inhibitors impeded STAT1- and IRF-1-independent pathways, interrupting M1 polarization and further preventing psoriasis progress.^689^ Studies have identified IRF2 as one of the potential susceptibility genes for psoriasis.^690,691^ Mice with IRF2 deficiency displayed psoriasis-like skin inflammation.^692^ In addition, Kawaguchi et al. illustrated that *Irf2*^+/-^ mice showed more severe imiquimod (IMQ)-induced skin inflammation, with higher levels of TNF-α, IL-12/23p40, IL-17A, and IL-22, compared to normal mice.^693^ Moreover, IRF2 haploinsufficiency created heightened biological responses to IFN-α, leading to enhanced angiogenesis and psoriasis-like inflammation within the skin.^693^ Li et al. presented that the STING/IRF3 pathway was activated in palmitic acid and IMQ induced human immortalized keratinocytes (HaCaT) cells.^694^ Furthermore, STING and p-IRF3 expression levels were significantly increased in patients skin with psoriasis and diabetes, as well as in diabetic mice skin with psoriasis, indicating that the STING/IRF3 pathway might regulate inflammatory response in psoriasis with diabetes mellitus.^694^ Ni et al. illustrated that IRF4 was significantly increased in keratinocytes and inflammatory cells in psoriasis vulgaris lesions compared to normal controls.^695^ Cai et al. reported that IRF4 activation promoted dermal γδT cell IL-17 production,^696^ which indicates that IRF4 may facilitate skin inflammation in psoriasis. Additionally, Nakao et al. uncovered that IRF5 deficiency caused IRF4 upregulation in DCs, followed by IL-23 augment, inducing Th17 response amplification and exacerbating IMQ-induced psoriasis-like skin inflammation.^697^ The RNA sequencing revealed overexpressed IRF7 in patients with psoriatic skin.^698,699^ Zdhhc2 was required by pDCs to induce IRF7 phosphorylation and IFN-α production in psoriasis-like inflamed murine skin.^700^ Besides, TBK1 activation and IRF7 nuclear migration facilitate IMQ-induced acute psoriasis-like inflammation.^701^ Moreover, the bromodomain and extraterminal domain inhibitor NHWD-870 could inhibit IRF7 and p-IRF7 expression to ameliorate IMQ-induced psoriasis-like inflammation.^699^ The microarray data analysis revealed that IRF8 was an important hub gene in psoriasis complicated with atherosclerosis,^702^ and the RNA sequencing analysis also revealed that IRF9 was a core transcriptional regulator associated with inflammation in psoriasis.^703^ In cases of atopic dermatitis, Gao et al. revealed that IRF2 variants were associated with atopic dermatitis and eczema herpeticum.^704^ A comprehensive bioinformatics analysis reported that IRF7 was upregulated in atopic dermatitis patients and identified it as a hub gene for atopic dermatitis.^705^

#### Systemic lupus erythematosus (SLE) and related complications

Studies have uncovered that overexpressed IRF1 could produce a pattern of hyperacetylation at H4 lysine residues and induce the expression of target genes in vitro, a finding also shown in SLE patients.^706–709^ Moreover, IRF1 overexpression in monocytes from SLE patients enhanced inflammasome activation.^710^ Chen et al. reported that HDAC1 inhibited miR-124 and then promoted IRF1 expression to potentiate CD4^+^ T cell activation in SLE.^711^ In lupus nephritis (LN) patients, an inverse correlation between IRF1 and miR-130b levels was observed in renal samples. Overexpression of miR-130b in vivo suppressed IRF1 expression and consequently ameliorated IFN-α-accelerated LN.^712^ A joint analysis of GWAS and replication data found the missense variant in IRF3 was associated with LN patients, and identified IRF3 as a novel locus for SLE.^713^ Xu et al. presented that circELK4 sponged miR-27b-3p, which facilitated STING/IRF3/IFN-I signaling activation and promoted inflammation, cell apoptosis, and renal injury in LN.^714^ Phosphorylation of IRF3/IRF7 by TonEBP in macrophages facilitated autoimmune responses in SLE/LN pathogenesis.^715^ Zheng et al. found that serine/threonine kinase AKT2 interacted with IRF3 and attenuated IRF3 nuclear translocation to reduce IFN-I production, which consequently prevented SLE development.^716^ Additionally, Faridi et al. revealed that CD11b activation with the agonist LA1 reduced the phosphorylation of AKT and its substrate FOXO3a to suppress IRF3/7-mediated gene expression, thereby suppressing TLR and IFN-I signaling-associated inflammation and autoimmunity in SLE.^717^ Besides, studies showed that IRF7 was an important transcript factor in the LN process, and the lupus risk allele in IRF7 was associated with significant IRF7 hypomethylation.^718–720^ IRF7 was required for autoantibody production in murine lupus development.^721^ Chandrasekaran et al. illustrated that IRF4 was required for the expansion and function of effector Treg cells to limit murine lupus-like disease development.^722^ However, Lech et al. also demonstrated that IRF4 promoted LN development in mice.^723^ Genetic variants within and around IRF5 were robustly associated with SLE risk.^724–728^ IRF5 was an early regulator of human B cell activation, and monoallelic IRF5 deficiency in B cells prevented murine lupus.^729,730^ Moreover, recent studies revealed that both genetic loss of *Irf5* and chemical inhibition ameliorated disease in murine lupus models.^731,732^ The IRF8 locus was also associated with an increased SLE risk.^733^ IRF8 was critical for the differentiation of MDSCs, which further impaired Treg differentiation and promoted Th17-cell polarization in SLE development.^297,734^ Studies also showed that IRF9 mRNA levels were increased in SLE monocytes, which was positively associated with both SLE activity and ISGs activity.^735,736^

#### Multiple sclerosis (MS)

Annibali et al. found IRF1 was downregulated in B cells from MS patients, and IRF1/CXCL10 axis downregulation might promote a pro-survival status of B cells in MS.^737^ On the other hand, Kortam et al. revealed serum IRF3 levels were upregulated in MS patients.^738^ Estrogen receptor alpha in DCs degraded TRAF3 via ubiquitination, reducing IRF3 nuclear translocation and transcription of membrane lymphotoxin and IFN-β components, which consequently alleviated disease severity in MS mice.^739^ Studies illustrated that IRF4 facilitated Th17 cells differentiation in relapsing-remitting MS,^740^ and miR-30a reduced IRF4 expression to inhibit Th17 differentiation and prevent the development of autoimmune encephalomyelitis (EAE) in mice, an animal model of MS.^741^ In addition, studies reported an IRF5 variant was associated with primary progressive MS, and variation near IRF6 was associated with IFN-β-mediated liver injury in MS.^742,743^ Studies also revealed that IRF8 was a risk gene for both EAE in mice and MS patients, and IRF8 SNP was associated with mitogen-activated protein kinase kinase 1 (MP2K1) phosphorylation levels, which were overactive in MS.^744–746^ IRF8 enhanced αvβ8 integrin expression in APCs and activated TGF-β signaling, resulting in Th17 cell differentiation and exacerbated neuroinflammation.^747^

#### Sjögren’s syndrome (SS) and rheumatoid arthritis (RA)

**Fig. 9:**
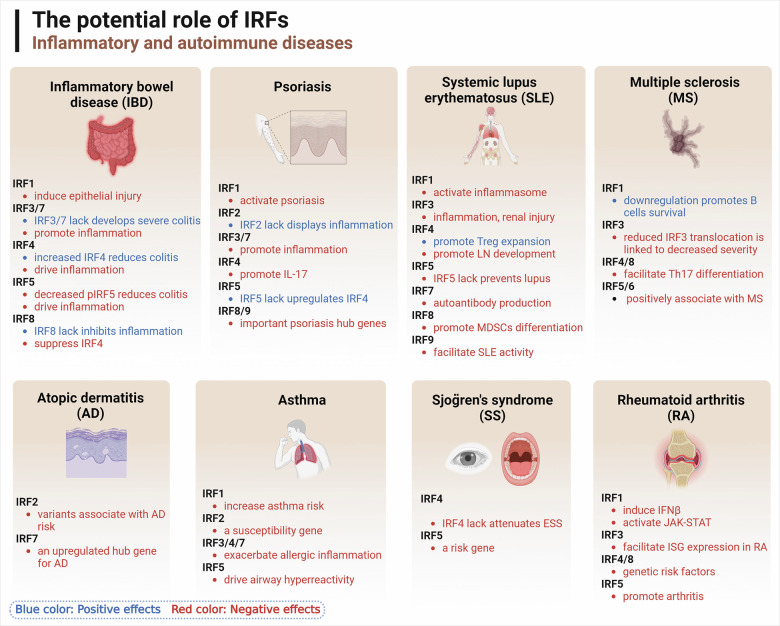
The potential role of IRFs in inflammatory and autoimmune diseases. The reported IRF family members show protective roles, negative roles, or dual roles in inflammatory bowel disease, psoriasis, systemic lupus erythematosus, and multiple sclerosis. On the other hand, the reported IRF family members almost play negative functions on atopic dermatitis, asthma, Sjögren’s syndrome and rheumatoid arthritis

Wei et al. found that IRF4 was upregulated in murine PMN-MDSCs during experimental SS (ESS) progression in mice and IRF4 deficiency facilitated aryl hydrocarbon receptor (AhR)-induced PMN-MDSC responses to attenuate ESS.^748^ Furthermore, Xiao et al. presented that artesunate suppressed IRF4-mediated glycolysis and increased proteasomal degradation of IRF4 to inhibit Th17 response and ameliorate ESS in mice.^749^ IRF5 was a risk gene in SS.^750,751^ In RA, IRF1 was reported to induce IFN-β expression, which further activated the JAK/STAT pathway.^752^ IRF3 was reported to be strongly associated with ISG expression in RA.^753^ IRF4, IRF5, and IRF8 were identified as genetic risk factors for RA.^754–758^ Moreover, Duffau et al. revealed that IRF5 promoted inflammatory arthritis in an RA mouse model.^759^ The Fig. 9 shows the potential role of IRFs in diverse inflammatory and autoimmune diseases.

### IRFs and metabolic diseases

#### Nonalcoholic fatty liver disease (NAFLD)

NAFLD includes all kinds of fatty liver disease, from isolated hepatic steatosis or NAFL to nonalcoholic steatohepatitis (NASH) and NASH cirrhosis.^760^ Patel et al. revealed that hepatic IRF3 directly targets protein phosphatase 2 scaffold subunit Abeta (Ppp2r1b) to inhibit glucose production, and it was activated in obese humans with NAFLD.^761^ Qiao et al. revealed that STING and IRF3 levels were increased in the livers of high-fat diet fed NAFLD-like mice models.^762^ The STING/IRF3 pathway activation promoted hepatocyte injury, dysfunction, and liver fibrosis through inducing inflammation and apoptosis, and impeding glucose and lipid metabolism.^762,763^ Besides, disruption of IRF3 activation by hepatocyte nuclear factor 1 A ameliorated the innate immune response and NAFLD/NASH in vitro.^764^ Tong et al. demonstrated that IRF6 acted as a protective factor against liver steatosis in hepatocytes, and that hepatic IRF6 suppressed peroxisome proliferator-activated receptor γ to alleviate liver steatosis and metabolic problems in NAFLD transgenic mice.^765^

#### A*lcoholic liver disease (ALD)*

The IRF1-induced caspase-1 and NADPH oxidase 2 (NOX2)-mediated ROS pathway could induce a gastrin-releasing peptide receptor pro-inflammatory and oxidative stress response in alcohol-associated liver injury (ALI).^766^ The degradation of IRF1 and the removal of damaged mitochondria by murine macrophage autophagy prevented ALD in mice.^767^ Patients with alcoholic hepatitis showed elevated p-IRF3 and IRF3-mediated signals in livers, and IRF3 enhanced immune cells apoptotic death, which then resulted in hepatocellular injury in ALD mice.^267^ Moreover, IRF3, activated by endoplasmic reticulum stress or ethanol, also contributed to hepatocyte apoptosis and inflammatory response in early ALD.^768^ cGAS/IRF3 pathway expression was positively correlated with ALD severity, and hepatic gap junctions propagated cGAS-mediated IRF3 activation to enhance alcohol liver injury in ALD models.^769,770^

#### Atherosclerosis (AS)

IRF1 was overexpressed in both human and mouse AS lesions,^771^ and it increased CCL19 expression, facilitating vascular smooth muscle cells (VSMCs) proliferation and migration in AS.^772^ In addition, IRF1 facilitated non-canonical NF-κB-NLRP3-mediated endothelial pyroptosis and AS progression.^773^ Inhibition of IRF1 suppressed modified lipoprotein uptake, promoted cholesterol efflux, and altered gene expressionrelated to lipid metabolism to prevent AS.^771^ Liu et al. demonstrated that *Irf3*^-/-^*Apoe*^-/-^mice presented significantly decreased AS lesions due to suppressed VCAM-1 and intercellular adhesion molecule 1 (ICAM-1) expression, which attenuated macrophage infiltration.^774^ IRF5 acted as a detrimental factor in AS through maintaining pro-inflammatory macrophages, restricting necrotic core expansion o by impairing efferocytosis, and inducing rupture-prone atherosclerotic plaques formation.^775,776^ IRF5-deficient macrophages facilitated a stable plaque phenotype generation to combat AS in mice.^777^ IRF7 activation enhanced AS progress, and repression of IRF7-dependent TLR9 responses in macrophage induced decreased proatherogenic CXCL10 production.^778^ Interruption of the RAGE/IRF7 pathway also triggered a switch from a pro- to an anti-inflammatory environment and accelerated AS regression.^779^ Deficiency of IRF8 in hematopoietic cells with a CML-like phenotype was presented to promote AS progress in mice.^780^ However, antoher study demonstrated that DCs-specific IRF8 deletion significantly reduced aortic T-cell accumulation and adaptive immune responses, resulting in the inhibition of AS development, especially in the aortic sinus.^781^

#### Acute coronary syndrome (ACS) and myocardial ischemia reperfusion (MIR)

Guo et al. suggested that IRF1 might induce Th1 cell differentiation and thus contribute to ACS pathogenesis in vitro.^782^ Furthermore, in ACS patients, IRF1 was found to be overexpressed in macrophages, facilitating macrophage pyroptosis and the downstream inflammatory response in AS and ACS.^783,784^ The knockdown of cardiac-specific IRF1 significantly reduced infarct size, improved cardiac function, and inhibited myocardial apoptosis after MIR injury, while the overexpression of cardiac-specific IRF1 obviously enhanced MIR injury in mice.^785^ IRF2-driven GSDMD-dependent pyroptosis and then contributed to MI in both in vivo and *vitro* models.^786^ Activated IRF3/IFN axis in MI cardiac macrophages improved survival.^787^ IRF3-dependent signaling interruption led to decreased inflammatory cell infiltration, cytokines, chemokines in the heart, and recovered cardiac function.^787,788^ It was reported that the TLR7/MyD88/IRF5 signaling pathway activation aggravated MIR injury in mice.^789^ The inhibition of the Dectin1/-Syk-IRF5 pathway interrupted M1 macrophage polarization and inflammation in both in vivo and in vitro coronary microvascular dysfunction models.^790^ In addition, reduced IRF5 expression accelerated both cutaneous and infarct healing and alleviated post-MI heart failure development after coronary ligation.^791^ The sequencing indicated that *IRF5* gene polymorphisms might be associated with ACS susceptibility in the Chinese population.^792^ IRF9 was upregulated in ischemic heart tissue after MIR injury, and IRF9 ablation prevented MIR-mediated cardiomyocyte inflammation, death, and heart dysfunction.^793^

#### Obesity and diabetes

**Fig. 10:**
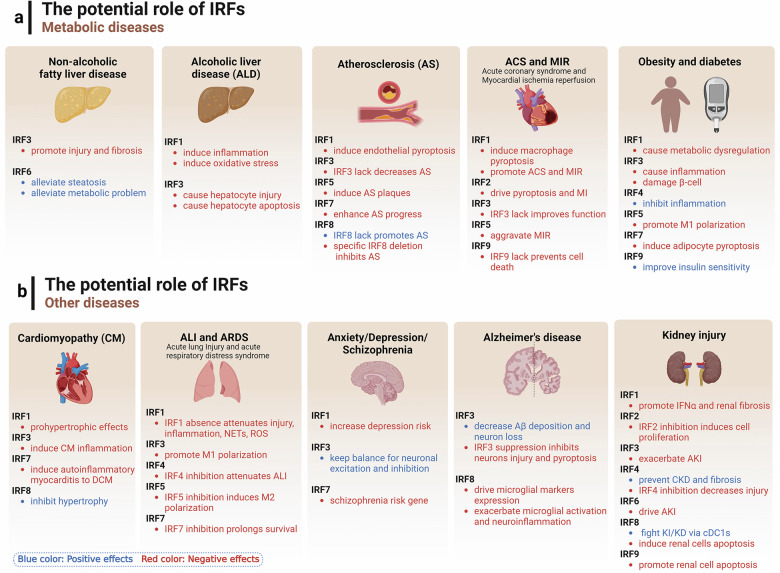
The potential role of IRFs in metabolic and other diseases. **a** The roles of IRFs in metabolic diseases. **b** The roles of IRFs in other diseases. In brief, IRF1, IRF2, IRF5, and IRF7 present negative roles in these diseases. IRF3, IRF4, and IRF8 play protective roles, negative roles, or dual roles in these diseases. IRF6 is protective in non-alcoholic fatty liver disease, while a negative factor in kidney injury. IRF9 plays negative role in acute coronary syndrome and myocardial ischemia reperfusion, and kidney injury, while IRF9 plays protective role in obesity and diabetes

Friesen et al. demonstrated that IRF1 expression in adipocytes contributed to the upregulating of obesity-related inflammatory processes and metabolic dysregulation both in vitro and in vivo.^794^ IRF3 expression was increased in the adipocytes of obese mice and humans. TLR3/4-IRF3 activation induced insulin resistance in murine adipocytes, while IRF3 knockdown prevented insulin resistance. This indicates that IRF3 facilitates adipose inflammation and insulin resistance and supresses browning.^795^ In addition, IRF3 is a strong repressor of adipose thermogenesis via ISG15-mediated reprogramming of glycolysis.^796^ IRF3 directly transcriptional regulates glucose homeostasis through induction of Ppp2r1b, and subsequent suppression of glucose production.^761^ Moreover, STING/IRF3 axis triggered endothelial inflammation in response to free fatty acid-mediated mitochondrial damage in diet-induced obesity.^797,798^ Furthermore, STING/IRF3 pathway activation triggered pancreatic β cells inflammation and apoptosis, causing β-cell damage and dysfunction, while STING or IRF3 silencing ameliorated inflammation and apoptosis, and reversed impaired insulin synthesis in type 2 diabetes.^799^ Eguchi et al. reported that mice with a myeloid cell-specific IRF4 knockout developed serious insulin resistance on a high-fat diet. Furthermore, Irf4^-/-^ adipose tissue macrophages enhanced M1 polarization,^800^ indicating that IRF4 negatively regulates inflammation in diet-induced obesity. Cavallari et al. also showed IRF4 was responsible for NOD2-induced insulin sensitizing and anti-inflammatory effects during obesity and endotoxemia.^801^ Studies have identified that IRF5 gene expression is positively associated with adipose inflammatory signatures in both obesity and diabetic obese patients.^802,803^ IRF5 facilitated M1 macrophage polarization and regulated mitochondrial architecture remodeling in obesity.^101,804^ Moreover, *Irf5* deficiency in macrophages promoted beneficial adipose tissue expansion and insulin sensitivity during obesity in vivo.^805^ Studies demonstrated that suppressing IRF7 expression restored mtRNA-induced mitobiogenesis and thermogenesis, improved glucose and lipid homeostasis and insulin sensitivity, and finally mitigated obesity.^806,807^ Additionally, GSDMD interacted with IRF7 and subsequently formed a complex to promote adipocyte pyroptosis.^808^ IRF7-STAT2 cascade activation facilitated IFN-α signaling expression in islets and amplified the process of type 1 diabetes.^809^ Wang et al. reported that IRF9 was more downregulated in the livers of obese mice than in controls. Hepatic IRF9 overexpression in obese mice significantly attenuated hepatic steatosis and inflammation, and improved hepatic insulin sensitivity.^810^ The Fig. 10 presents the potential role of IRFs in diverse metabolic diseases.

### IRFs and other diseases

There are several studies uncovered the possible relationships between IRFs and other kinds of human diseases involved in the heart, lungs, kidneys, and mental and nervous systems (Fig. 10).

#### Cardiomyopathy

IRF1 elicited cardiac prohypertrophic effects by directly activating iNOS.^811^ Studies illustrated that the activated cGAS-STING pathway and downstream targets like IRF3 overexpression induced cardiomyocyte inflammation, apoptosis, and pyroptosis in diabetic cardiomyopathy (DCM) mouse model.^812,813^ Xie et al. showed that IRF3 suppression by ZNF593-AS ameliorated cardiac cell death and inflammation in DCM.^814^ Additionally, STING/IRF3 axis activation could activate NLRP3 and exert apoptosis, pyroptosis, and inflammation in sepsis and sepsis-induced cardiomyopathy (SIC) models.^815^ Gonzalez et al. identified IRF7 as the main mediator of autoinflammatory responses caused by ADAR1 absence in cardiomyocytes, resulting in late-onset autoinflammatory myocarditis progressing into DCM.^816^ IRF8 was found to inhibit calcineurin signaling and thus inhibit the hypertrophic response.^48^

#### Acute Lung Injury (ALI) and Acute respiratory distress syndrome (ARDS)

Wu et al. uncovered that IRF1 facilitated caspase1-mediated pyroptosis in AMs and induced downstream inflammatory cytokine release. IRF1 knockout mice significantly abrogated pyroptosis in AMs and attenuated LPS-induced lung injury and systemic inflammation.^817^ Moreover, IRF1 deficiency strongly alleviated neutrophil extracellular traps (NETs) generation and ROS production in neutrophils from bronchoalveolar lavage fluid.^818^ Chen et al. showed that endothelial-specific Irf1 knockout in ALI mice presented lung endothelium regeneration. Irf1 transactivation by LPS-induced Stat1Ser727 phosphorylation upregulated leukemia inhibitory factor (Lif), indicating that p-Stat1/Ser727/Irf1/Lif axis inhibited lung endothelial cell regeneration post-LPS injury, and IRF1 or LIF inhibition might be a promising option for improving endothelial cell regeneration and clinical outcomes in ARDS patients.^819^ Wang et al. presented that the STING/IRF3 pathway activation contributed to paraquat-induced ALI.^820^ In addition, the TBK1/IRF3 and other related pathways activation by the STING agonist diABZI amplified the inflammatory loop in ARDS.^821^ Zhang et al. found that *IFIH1* was strongly connected with ARDS, and IFIH1 could activate IRF3 to promote M1 polarization in vitro.^822^ Anisodamine was shown to inhibit G9a-mediated methylation and IRF4 expression, promoting macrophage M2 polarization and inhibiting M1 polarization, which then attenuated LPS-induced ALI and pulmonary edema.^823^ IRF5 inhibition induced M2 polarization to improve ALI/ARDS in vitro.^824^ A bioinformatics analysis identified that IRF7 might regulate critical processes in ALI pathogenesis.^825^ Furthermore, reduced IRF7 levels or activity in the lungs of mice led to decreased IFNα mRNA levels, reduced neutrophil infiltration in the lungs, and prolonged survival of IAV-induced ALI mice.^826^

#### Kidney injury and kidney-related diseases

IRF1 was found to cause ROS-mediated IFN-α production in murine ischemic acute kidney injury (AKI) and was an important early pro-inflammatory signal during ischemic AKI in vitro and in vivo.^827,828^ Yan et al. illustrated that lncRNA NEAT1 inhibited miR-130a-3p to upregulate IRF1 signaling and activate TLR4/NF-κB pathways, facilitating oxidative damage during calcium oxalate (CaOx) crystal deposition.^829^ Moreover, studies uncovered that inhibition of IRF1 with HIF-1α or TLR4 expression by AhR or sulforaphane resulted in M1 polarization inhibition and M2 polarization progress, and finally alleviated renal CaOx crystal deposition and nephrocalcinosis-induced kidney inflammation and injury in vitro and in vivo.^830–832^ IRF1 was also found to be overexpressed in fibrotic kidney of chronic kidney disease (CKD) patients and was identified as a driver for fibrosis, which repressed Klotho expression to promote renal fibrosis in vitro and in vivo.^833,834^ A GWAS identified SNPs near *IRF2* were associated with AKI,^835^ while Renken et al. reported that there was no association between genetic loci near IRF2 and AKI in the critically Ill.^836^ Zhang et al. demonstrated that IRF2 was increased in the serum of sepsis-mediated AKI patients and LPS-induced HK-2 cells. IRF2 downregulation induced cell proliferation and inhibited cell death, and IRF2 knockdown inhibited LPS-treated HK-2 cell pyroptosis by upregulating the expression of caspase-4, caspase-11, and GSDMD. In the caecal ligation and puncture (CLP)-induced *Irf2*^-/-^ animal models, the survival rate was increased, and pathological features and scores were alleviated.^837^ Similarly, He et al. showed that IRF2 knockdown played anti-inflammatory and antioxidant stress functions to further decrease LPS-induced renal tissue injury in vivo and in vitro.^838^ IRF3 activation triggered the Hippo pathway and then impeded proliferation and repair in surviving renal tubular epithelial cells, and exacerbated LPS-induced AKI progress in vitro cell model.^839^ Overexpressed TRIM3 inhibited the IRF3 pathway and NLRP3 inflammasome activation to prevent LPS-induced AKI in rat models.^840^ Moreover, STING/TBK1/IRF3/NF-κB signaling activation in dsDNA-induced Aim2-deficient macrophages aggravated inflammatory phenotypes in a rhabdomyolysis-induced AKI mouse model.^841^ A genome-wide meta-analyses showed that IRF4 risk loci participated in membranous nephropathy and IgA nephropathy (IgAN) pathogenesis.^842,843^ IRF4 was overexpressed and was a hub gene in drug-induced AKI cell and mouse models, as well as in human specimens based on integrated transcriptomic analysis.^844^ Irf4 deletion in myeloid cells inhibited AKT-related monocyte recruitment to the injured kidney, and decreased activation and subsequent profibrotic M2 polarization to protect against tubulointerstitial fibrosis development after severe ischemia-reperfusion injury (IRI) in mice.^845^ Similarly, other studies also found that inhibition of IRF4 disrupted macrophage to myofibroblast differentiation in the kidneys under a folic acid (FA)-induced AKI-CKD transition mouse model, and protected mice against kidney inflammation and fibrosis in deoxycorticosterone acetate/salt hypertension.^846,847^ However, Lorenz et al. showed that IRF4 prevented CKD and kidney fibrosis following IRI, and Irf4^–/–^ mice presented chronic intrarenal inflammation, tubular epithelial cell loss, and renal fibrosis after IRI.^847,848^ Liu et al. reported that ENSMUST_147219 sponged miR-221-5p to upregulate IRF6 expression to drive apoptosis and promote ischemic AKI development.^849^ IRF8 was a risk locus that caused abnormal IgA levels and a high polygenic score for IgAN was associated with an earlier onset of kidney failure.^850^ IRF8 upregulated renal tubular cell apoptosis in cisplatin-induced AKI, and that hypermethylation in the Irf8 promoter region, which repressed Irf8, could protect against cisplatin-induced AKI mice.^851^ However, Li et al. identified that IRF8 was indispensable for kidney cDC1s development, and that IRF8-mediated cDC1s mildly protect mice against post-ischemic AKI/AKD.^852^ The study by Liu et al. showed that IRF9 downregulated SIRT1 expression and increased acetylated p53 to promote rat renal cell apoptosis in hyperlipidemia acute pancreatitis associated with KI.^853^

#### Mental and nervous system diseases

IRF1 was associated with an increased risk of the development of depression.^854^ IRF3 was reported to be critical in maintaining the balance between neuronal excitation and inhibition, and a lack of IRF3-induced anxiety/depression-like behaviors in mice.^855^ IRF3 and IRF7 were identified as risk candidate genes for schizophrenia.^856,857^ Xu et al. uncovered that cGAMP/STING/IRF3 pathway stimulation induced triggering receptor expressed on myeloid cells 2 (TREM2) expression, which further decreased amyloid-β (Aβ) deposition and neuron loss to improve Alzheimer’s disease (AD) pathomorphology and cognitive impairment.^858^ However, Guo et al. showed that IRF3 expression and phosphorylation were significantly elevated during the development of AD, and the silencing of ZBP1 could suppress IRF3 to inhibit cell injury and pyroptosis of neurons, thereby improving cognitive function in rats with AD.^859^ Additionally, silencing IRF3 alleviated chronic neuropathic pain after chronic constriction injury by inhibiting NF-κB signaling activation in rats.^860^ Studies showed that IRF8 drove the expression of microglial markers linked to AD, and IRF8 overexpression exacerbated microglial activation and neuroinflammation in AD.^861,862^

## Concluding remarks and future perspectives

This review provides a thorough examination of the structure, post-transcriptional modification sites, functional roles, and signaling pathways involving nine IRF family members across diverse cells, tissues, and human diseases. IRF family members exhibit multifaceted functions with certain activities being mutually synergistic or antagonistic. Additionally, IRF family members influence various signaling pathways differently across distinct cell types. Moreover, IRFs play complex and varied roles in immune cells, stem cells, tumor cells, and in the context of inflammatory and autoimmune diseases by engaging in numerous signaling pathways, with their regulation varying based on cell type, cellular environment, and interactions between IRF members. In immune cells, IRF family members are pivotal in the regulation of immune responses, primarily through their participation in the IFN signaling pathway. During viral infections, virus recognition receptors activate IRF3 and IRF7, which initiates IFN-I expression and the antiviral immune response. IRF4 is particularly critical for Th cell differentiation, while IRF8 plays a critical role in the maturation of bone marrow cells. In some cases, IRF1 can induce apoptosis and inhibit tumor cell growth by promoting the expression of apoptosis-associated genes, while IRF2 can promote cell survival and tumor proliferation by inhibiting the activity of IRF1. IRF5 is important in orchestrating the inflammatory response and in triggering autoimmune diseases via the activation of pro-inflammatory cytokines like IL-6 and TNF-α. In concert with STAT1 and STAT2, IRF9 forms the ISGF3 complex, which is involved in the IFN signaling pathway, regulating gene expression essential for antiviral defense and the control of autoimmune diseases.

In summary, IRF proteins serve critical functions in both human health and disease, attributed to their extensive engagement in a variety of physiological and pathological mechanisms. Research indicates that IRF family members are expressed aberrantly in an array of diseases, notably infections, inflammatory conditions, and a spectrum of cancers, affecting numerous systems including but not limited to the cardiovascular, pulmonary, urinary, reproductive, and integumentary systems. Depending on the cell types and the surrounding environment, IRF proteins can exert markedly different regulatory effects, rendering their roles intricate, and they may as double-edged swords in human health. For example, in infectious diseases, nearly all IRF members display protective functions that combat pathogens. However, in certain scenarios, activation of IRFs can trigger an inflammatory response, leading to damage within the body. Similarly, in the context of cancer, certain IRF members function as tumor suppressors, while others may behave as oncoproteins. Besides, there are IRF members that not only enhance antitumor responses but also aid in cancer immune evasion. The reasons for these discrepancies remain elusive and need further in-depth research.

Moreover, IRF proteins may function as biomarkers and therapeutic targets for diverse human diseases. Mouse models with knockout of IRF family genes exhibit a diverse array of phenotypes affecting immune system development and function, antiviral and anti-tumor immune responses, as well as cell differentiation and development (Table 1). Investigating these phenotypes provides valuable insights for identifying potential therapeutic targets for various diseases. Although therapeutic strategies on IRFs are mainly limited to indirect modulation, several direct inhibition and activated strategies have been reported in recent years (Table 2), which hold immunotherapeutic potential in the treatment of infections, inflammatory conditions, and tumors. More clinical researches are needed to explore their therapeutic potential. Therapies that augment IRF activity may be advantageous in combating a wide spectrum of pathogens; however, they could also pose significant risks to the host if uncontrolled immune activation leads to autoimmune-like disease. Considering the diverse roles played by IRF family members, unraveling the intricate mechanisms that underpin both their protective and deleterious effects is anticipated to yield valuable insights into the biology of microbial infections and host defense, autoimmune and inflammatory diseases, as well as cancers.

**Table 1:** Phenotype of Mouse genetic models

IRFs	Mouse genetic models	Phenotype	Reference
IRF1	IRF1^−/−^	Significantly attenuates high phosphate-induced alterations; less resistant than normal mice to EMCV infection, and knockout of IRF1 markedly alleviated cecal ligation and puncture -induced lung injury and M1-polarized infiltration	^189,863,864^
	IRF1 silencing	Alleviated bevacizumab -induced cardiomyocyte injury by regulating the VEGFA/14-3-3γ axis	^865^
	IRF-1^–/–^	Can be transformed by expression of an activated c-Ha-ras oncogene	^866^
	IRF1^–/–^	Bone marrow cells exhibit an increased number of immature granulocytic precursors	^867^
	IRF1 deficiency	TCRαβ^+^CD4^-^CD8^+^ T cells were significantly reduced, and thymus cell development was defective. Th1 cell differentiation deficiency	^230,232,868,869^
IRF2	IRF-2-deficient mice	Fail to control virus replication and recruit immune infiltrates into the brain. Mice spontaneously develop inflammatory skin disease as they age, and die within weeks from LCMV infection	^230,870,871^
	IRF2^–/–^	Severely compromised development of natural killer and Th1 cells; exhibited a marked and selective defect in splenic CD4^+^CD11b^+^DCs	^159,872^
IRF3	IRF3 knockout	Develop obesity, insulin resistance, glucose intolerance, and eventually type 2 diabetes with aging	^873^
	Mutant IRF3	A mutant IRF3 defective in both the transcriptional and the apoptotic activities was active in RIKA and inhibited virus replication	^327^
	IRF3^–/–^	Sendai virus infection caused enhanced inflammation in the lungs	^327^
	IRF3 deficiency	Exhibited lethal defects in the inflammatory and recovery phases of the colitis, accompanied by marked defects in the gene induction for thymic stromal lymphopoietin; TMEV-specific memory T cells expressing granzyme B (GrB) were significantly lacking	^874,875^
IRF4	Loss of IRF4	Not conducive to the expansion and differentiation of virus-specific NK cells	^876^
	IRF4 deficiency	Could not clear L. monocytogenes infection and generated decreased numbers of L. monocytogenes-specific CD8^+^ T cells with impaired effector phenotype and function; display increased tubular cell loss and defective clearance of infiltrating macrophages, and showed defective development of alternatively activated macrophages	^848,877^
IRF5	Conditional KO IRF5	Suppresses SLE progression	^731^
	IRF5^–/–^	Show higher susceptibility to viral infection, develop an age-related splenomegaly, and Splenic B cells also exhibited a decreased level of plasma cells	^878^
IRF6	IRF6 deficiency	Abnormal skin, limb and craniofacial development	^278^
	Simultaneously carrying a heterozygous deletion of p63 and the Irf6 knockin mutation R84C	Displayed ectodermal abnormalities that led to cleft palate	^879^
	Homozygous missense mutation in irf6	Hyperproliferative epidermis that fails to undergo terminal differentiation, resulting in soft tissue fusions	^279^
IRF7	IRF7 knockout	Demonstrated attenuated dermal fibrosis and inflammation compared with wild-type mice in response to bleomycin	^880^
	IRF7^–/–^	AML-IRF7^–/–^mice exhibited accelerated disease progression with intracerebral invasion of AML cells	^551^
	IRF7 deficiency	Caused significant elevation of granulocytic myeloid-derived suppressor cells, thus enhanced tumor growth and metastasis in mice	^214^
IRF8	Null mutation in IRF8/ Irf8^-/-^	Deficiency in the ability of myeloid progenitor cells to mature into macrophage lineage, and eventually to the leukemic phenotype; develop a CML-like disease	^174,881^
	Heterozygous and homozygous Irf8 knock-in	Mice demonstrated significantly increased osteoclast formation and resorption activity in vivo and in vitro	^203,882^
IRF9	Whole-body IRF9 knockout	More obese and had aggravated insulin resistance, hepatic steatosis, and inflammation after chronic high-fat diet feeding	^810^
	IRF9 overexpressing	Were subjected to warm I/R of the liver	^883^
	IRF9 deficiency	Markedly reduced the necrotic area, serum alanine amino transferase/aspartate amino transferase, immune cell infiltration, inflammatory cytokine levels, and hepatocyte apoptosis after liver I/R	^883^

**Table 2:** Direct target for IRF family

Name	Structure	Function	Mechanism	Clinical stage	Conditions	Trial registration
Geldanamycin (GA)^884^		IRF3 inhibitor	Inhibits heat-shock protein 90 (Hsp90) of the IRF3 phosphorylation chaperone	NA	NA	NA
KIN1148^885,886^		IRF3 agonist	Induces dose-dependent IRF3 nuclear translocation and specific activation of IRF3-responsive promoters and functions as an influenza vaccine adjuvant	NA	influenza A virus (IAV) H1N1 infection	NA
ION251^587^	NA	selective IRF4 inhibitor	IRF4 antisense oligonucleotides	Phase I	malignant myeloma (MM)	ClinicalTrials.gov NCT04398485
Compound C5^887^	NA	selective IRF5 inhibitor	Reduces IRF5 nuclear translocation	NA	systemic lupus erythematosus(SLE)	NA
YE6144^731^		selective IRF5 inhibitor	Inhibits the phosphorylation of IRF5	NA	SLE	NA
porcine fusion protein IRF7/3(5D)^888^	NA	a chimeric construct of porcine IRF7 and IRF3	A biotherapeutic and enhancer of IFN activity against foot-and-mouth disease virus (FMDV)	NA	foot-and-mouth disease virus	NA
